# Upregulation of WDR6 drives hepatic de novo lipogenesis in insulin resistance in mice

**DOI:** 10.1038/s42255-023-00896-7

**Published:** 2023-09-21

**Authors:** Zhenyu Yao, Ying Gong, Wenbin Chen, Shanshan Shao, Yongfeng Song, Honglin Guo, Qihang Li, Sijin Liu, Ximing Wang, Zhenhai Zhang, Qian Wang, Yunyun Xu, Yingjie Wu, Qiang Wan, Xinya Zhao, Qiuhui Xuan, Dawei Wang, Xiaoyan Lin, Jiawen Xu, Jun Liu, Christopher G. Proud, Xuemin Wang, Rui Yang, Lili Fu, Shaona Niu, Junjie Kong, Ling Gao, Tao Bo, Jiajun Zhao

**Affiliations:** 1 Department of Endocrinology, Shandong Provincial Hospital Affiliated to Shandong First Medical University, Jinan, China; 2 Shandong Clinical Research Center of Diabetes and Metabolic Diseases, Jinan, China; 3 Shandong Key Laboratory of Endocrinology and Lipid Metabolism, Jinan, China; 4 Shandong Prevention and Control Engineering Laboratory of Endocrine and Metabolic Diseases, Jinan, China; 5 Central Laboratory, Shandong Provincial Hospital Affiliated to Shandong First Medical University, Jinan, China; 6 Department of Pathology, Shandong Provincial Hospital Affiliated to Shandong First Medical University, Jinan, China; 7 Medical Science and Technology Innovation Center, Shandong First Medical University & Shandong Academy of Medical Sciences, Jinan, China; 8 Department of Radiology, Shandong Provincial Hospital Affiliated to Shandong First Medical University, Jinan, China; 9 Department of Hepatobiliary Surgery, Shandong Provincial Hospital, Shandong University, Jinan, China; 10 Department of Ultrasound, Shandong Provincial Hospital Affiliated to Shandong First Medical University, Jinan, China; 11 Shandong Provincial Hospital, School of Laboratory Animal & Shandong Laboratory Animal Center, Science and Technology Innovation Center, Shandong First Medical University & Shandong Academy of Medical Sciences, Jinan, China; 12 Institute of Genome Engineered Animal Models, Shandong Provincial Hospital Affiliated to Shandong First Medical University, Jinan, China; 13 Center of Cell Metabolism and Disease, Jinan Central Hospital, Shandong First Medical University, Jinan, China; 14 Department of Liver Transplantation and Hepatobiliary Surgery, Shandong Provincial Hospital Affiliated to Shandong First Medical University, Jinan, China; 15 Lifelong Health, South Australian Health & Medical Research Institute, North Terrace, Adelaide, South Australia Australia

**Keywords:** Endocrine system and metabolic diseases, Endocrine system and metabolic diseases, Metabolism

## Abstract

Under normal conditions, insulin promotes hepatic de novo lipogenesis (DNL). However, during insulin resistance (IR), when insulin signalling is blunted and accompanied by hyperinsulinaemia, the promotion of hepatic DNL continues unabated and hepatic steatosis increases. Here, we show that WD40 repeat-containing protein 6 (WDR6) promotes hepatic DNL during IR. Mechanistically, WDR6 interacts with the beta-type catalytic subunit of serine/threonine-protein phosphatase 1 (PPP1CB) to facilitate PPP1CB dephosphorylation at Thr316, which subsequently enhances fatty acid synthases transcription through DNA-dependent protein kinase and upstream stimulatory factor 1. Using molecular dynamics simulation analysis, we find a small natural compound, XLIX, that inhibits the interaction of WDR6 with PPP1CB, thus reducing DNL in IR states. Together, these results reveal WDR6 as a promising target for the treatment of hepatic steatosis.

## Abstract (web summary)

Yao and Gong et al. identify WD40 repeat-containing protein 6 (WDR6) to be upregulated in the liver of insulin-resistant mice. WDR6 contributes to promoting hepatic de novo lipogenesis during insulin resistance by upregulation of fatty acid synthase, and the authors identify a small molecule to inhibit this effect of WDR6 and reduce hepatic steatosis.

## Main

Hepatic steatosis is one of the most common chronic liver disorders and is often associated with obesity and IR^1,2^. The global prevalence and incidence of hepatic steatosis have been increasing over the last few decades, especially in China and among Western countries^3,4^.

The pathogenesis of hepatic steatosis is complicated and not fully elucidated. IR is considered a main pathogenic factor in its development^5,6^. Under physiological conditions, insulin inhibits liver gluconeogenesis and promotes DNL to store calories. During IR, insulin fails to suppress hepatic glucose production. However, a positive correlation also exists between insulin levels and liver lipid deposition^7^. Horst et al. found that in individuals with IR, hepatic insulin signalling exhibited a proximal block from the level of the insulin receptor (INSR) through both glucose and lipogenesis pathways^8^. Hepatic steatosis is regarded as a secondary effect of systemic IR; for example, the lipid content of adipose tissue (AT) exceeds its lipid storage capacity^9^. In AT, IR leads to a failure of insulin to suppress lipolysis^10^, thus accelerating ectopic lipid deposition in the liver^11^. Moreover, IR-induced AT disorders can lead to a lack of adipokines and subsequently to dysfunctional liver lipid metabolism^12,13^. However, another viewpoint is that during IR, while the arm of insulin inhibiting gluconeogenesis is blunted, the arm of promoting DNL is retained^14,15^, implying that other signalling of lipid metabolism may exist. Smith et al. observed human hepatic DNL showed a negative correlation with hepatic and whole-body insulin sensitivity, but positively correlated with plasma glucose/insulin concentrations^16^. Moreover, it has been reported that in preclinical IR mouse models, such as *ob/ob* or in diet-induced obesity models, liver-specific deletion of INSR is sufficient to lower the expression of sterol regulatory element-binding protein 1c (SREBP1c), a key transcription factor involved in the regulation of hepatic DNL^7^. These results indicate that liver insulin signalling is indispensable for excess hepatic DNL during IR^11^. Overall, it is well accepted that during IR there are still unrestrained increases in hepatic DNL^16,17^, but the mechanism by which hepatic steatosis increases remains incompletely understood.

The human WDR6 protein is WD40 repeat (WDR)-containing protein. The WD40 repeat is a conserved protein domain consisting of 40–60 amino acid residues. Its fundamental function is to coordinate the assembly of multiple protein complexes. Proteins containing such repeats are widely distributed in various tissues^18,19^. We explored its role along these lines here and found that WDR6 is upregulated during IR induced by feeding mice a high-fat diet (HFD) and subsequently promotes hepatic DNL by upregulating fatty acid (FA) synthase (FASN), a key metabolic enzyme involved in hepatic DNL. We also identified a small molecule that could block the relevant effects of WDR6 and thus reduce hepatic steatosis during IR. Together, these results point to a role for WDR6 in elevated DNL under IR states and suggest a potential therapeutic avenue to reduce fatty liver disease.

## Result

### *WDR6* is upregulated in response to insulin during insulin resistance

**Fig. 1:**
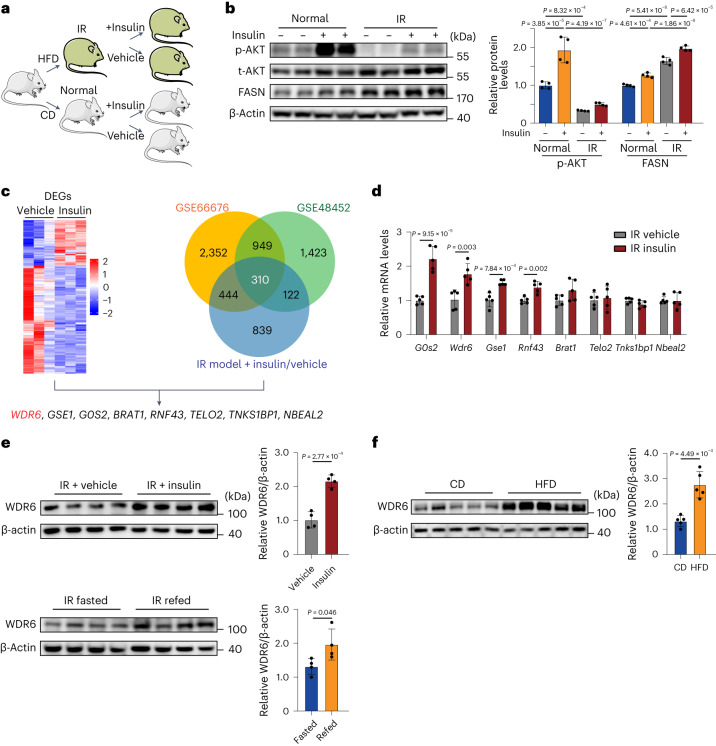
*WDR6* is upregulated in response to insulin in insulin-resistant states. **a**, Schematic illustrating the groups and procedures of the IR model. Mouse icons produced using Servier Medical Art by Servier (https://smart.servier.com/) under a Creative Commons Attribution 3.0 Unported Licence (https://creativecommons.org/licenses/by/3.0/). **b**, Western blots of phosphorylation of AKT at Ser473, a ‘classic’ downstream molecule of insulin action and of FASN levels in liver in the indicated groups. *n* = 4 biologically independent mice per group. β-actin served as a loading control. **c**, The strategy for identification of candidate genes for insulin-induced lipogenesis in liver. Transcriptomic analysis of DEGs in liver of insulin-treated and vehicle-treated groups during IR. Left: *n* = 3 biologically independent mice per group. Right: Venn diagram showing the common set of 310 genes identified at the overlap of two public datasets and our transcriptome data. **d**, RT–PCR analysis of the response of the candidate genes to insulin stimulation in liver during IR states. Expression levels were normalized to *Actb* mRNA levels. *n* = 5 biologically independent mice per group. **e**, Western blots of hepatic WDR6 in insulin-treated and vehicle-treated IR mice and fasted/refed IR mice. *n* = 4 biologically independent mice per group. **f**, Western blots of hepatic WDR6 after 28 weeks in the HFD-induced NAFLD mouse model and control mice. *n* = 5 biologically independent mice per group. Experiments in **b**–**f** were performed using male mice. Data in **b** are presented as the mean ± s.d., determined by two-way analysis of variance (ANOVA) and Tukey’s multiple-comparisons test. Data in **d**–**f** are presented as the mean ± s.d., determined by unpaired two-sided Student’s *t*-test. Source data

We created an IR mouse model by feeding animals a HFD for 6 weeks^20^. Insulin injection was then performed (Fig. 1a). The phosphorylation of AKT, a key readout of the insulin signalling pathway^21^, showed decreased response to insulin, indicating resistance to insulin (Fig. 1b). However, FASN continued to show the same response to insulin in HFD mice (that is, elevation). The results indicate that insulin can still drive expression of this lipogenic gene during IR.

To identify genes that still responded to insulin in the IR state, we performed liver transcriptome analysis in the insulin-injected and vehicle-injected IR mice. In total, 263 differentially expressed genes (DEGs) were identified (Fig. 1c and Supplementary Data Fig. 1). To narrow down the number of candidates of interest, we took advantage of the public gene expression profile datasets, GSE66676 and GSE48452, which are microarray databases of adults and adolescents with non-alcoholic fatty liver disease (NAFLD)/non-alcoholic steatohepatitis (NASH), respectively. A total of 310 genes were found to intersect in the three databases and showed positive association with *FASN* levels (Fig. 1c). Among these genes, we focus on those that could respond to insulin during IR, according to DEGs from our transcriptome analysis. Finally, 8 candidate genes were selected (Fig. 1c). Quantitative PCR with reverse transcription (RT–qPCR) was performed in IR mice with insulin injection (Fig. 1d), together with a literature survey to identify genes with links to insulin signalling and/or metabolism. It reported that WDR6, one of the 8 candidate genes, increases in mouse hypothalamus-derived GT1-7 cells treated with either insulin-like growth factor-1 (IGF-1) or insulin^22^. Moreover, in HeLa cells, WDR6 can interact with LKB1, a key Ser/Thr kinase that regulates energy metabolism by activating AMPK^23,24^. These reports suggested WDR6 might respond to insulin and participate in metabolism. Finally, we selected *Wdr6* for further study.

**Extended Data Fig. 1:**
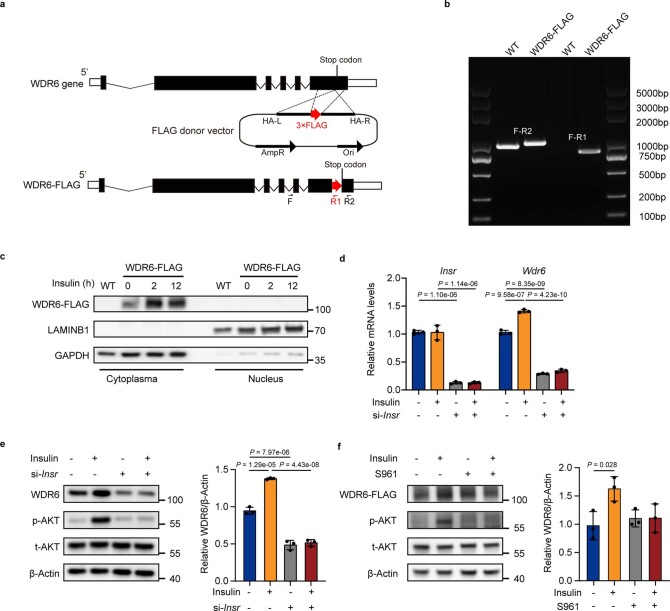
WDR6 responds to insulin stimulation via insulin receptor, related to Fig. 1. **a**, A schematic diagram illustrating the strategy for constructing genetically stable HepG2 cells with endogenous expression of WDR6-FLAG fusion protein. **b**, PCR identification of cells in (**a**). **c**, The effects of insulin stimulation on WDR6-FLAG protein levels and the distribution of endogenous WDR6-FLAG protein were detected by western blots. LAMINB1 and GAPDH served as a loading control for nucleus and cytoplasmic fractions, respectively. **d**, qPCR analysis of *Wdr6* mRNA levels in siRNA-mediated *Insr* knockdown cells with or without insulin stimulation. n = 3 independent experiments. *Actb* serves as a normalization control. **e**, Western blots of WDR6 protein level in cells described in (**d**). n = 3 independent experiments. β-Actin serves as a loading control. **f**, Western blots of WDR6-FLAG protein levels and AKT phosphorylation levels in WDR6-FLAG cells treated with S961 (the INSR inhibitor, 10 nM) for 4 h prior to treatment with insulin (100 nM, 2 h). n = 3 independent experiments. β-Actin served as a loading control. Data in **(d-f)** is presented as mean ± SD, determined by two-way ANOVA and Tukey’s multiple-comparisons test. * *P* < 0.05, ** *P* < 0.01. Source data

This finding was further validated by directly measuring WDR6 protein level in livers of IR mice with insulin fluctuation. The level of WDR6 increased after insulin injection or 6 h refeeding, which corresponded to conditions with increased exogenous or endogenous insulin levels, respectively (Fig. 1e). Additionally, we found hepatic WDR6 expression increased in NAFLD mice, which supported the hypothesis that WDR6 is involved in liver lipid metabolism (Fig. 1f). Next, the response of WDR6 to insulin in cultured cells was checked. We constructed a genetically stable HepG2 cell line with endogenous expression of a WDR6–FLAG fusion protein (in regulation of endogenous WDR6 promoter; WDR6–FLAG cells; Extended Data Fig. 1a,b). The cells were therefore treated with insulin to illustrate not only WDR6’s response to insulin, but also the cellular localization of WDR6. The result showed that WDR6 expression is strongly upregulated after insulin treatment and that it localized mainly to the cytoplasm (Extended Data Fig. 1c). We then performed insulin stimulation in cells with siRNA-mediated knockdown of *Insr*, or cells treated with S961, a specific antagonist of the INSR^25^. We found that insulin raised the expression of WDR6, and this effect was blunted when cells were treated with either *Insr* siRNA or S961 (Extended Data Fig. 1d–f). These results, in vivo and in vitro, demonstrate that in IR, hepatic *Wdr6* responds specifically to insulin via INSR signalling.

### Depletion of *Wdr6* protects against hepatic steatosis in insulin resistance

**Fig. 2:**
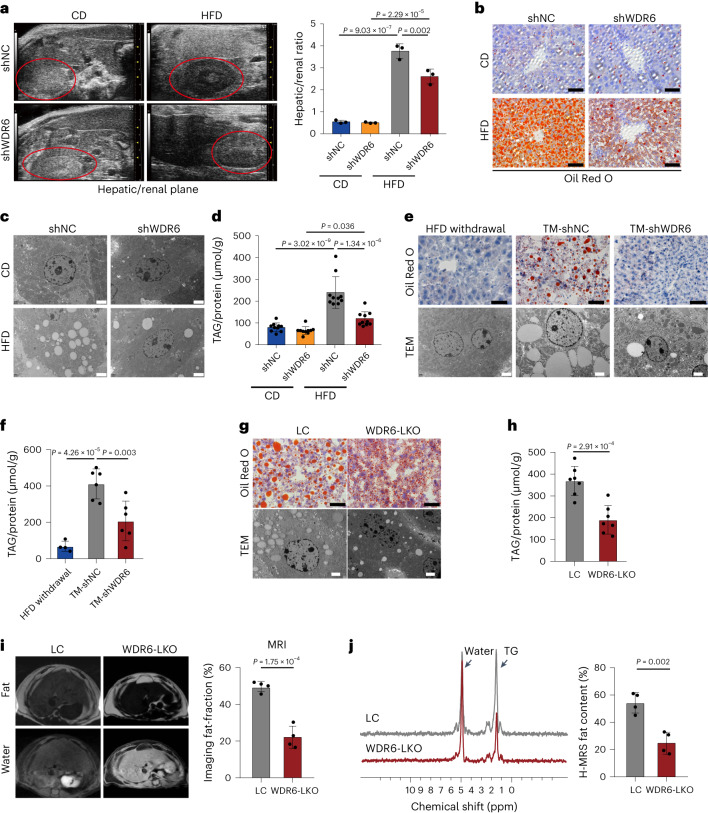
Liver-specific intervention of *Wdr6* strongly protects against excessive liver triacylglycerol deposition during high-fat-diet-induced insulin resistance. **a**, Liver B-mode ultrasound imaging of hepatic/renal plane (area circled in red represents the kidney) of AAV-shWDR6 and the corresponding controls. AAV-shNC mice after CD or HFD feeding for 8 weeks. *n* = 3 biologically independent mice per group. **b**, Representative ORO staining of liver sections in the indicated group in **a**. Scale bars, 50 μm. **c**, Representative TEM analysis of liver tissues in the indicated group. Scale bars, 2 μm. **d**, Liver TAG levels in the indicated group from **a**; the levels were normalized to the protein content of the same sample, *n* = 10 biologically independent mice per group. **e**, Representative ORO staining and TEM imaging were performed in liver tissues of the TM and the corresponding control. Scale bars (black), 50 μm; Scale bars (white), 2 μm. **f**, Liver TAG levels of the mice described in **e**, which were normalized to the protein content of the same sample. *n* = 4 biologically independent mice in the HFD withdrawal group and *n* = 6 biologically independent mice in the other group. **g**, Representative ORO staining and TEM imaging of liver tissues in WDR6-LKO and the corresponding LC mice. Scale bars (black), 50 μm; scale bars (white), 2 μm. **h**, Liver TAG level of WDR6-LKO and LC mice; the amount was normalized to the protein content of the same sample. *n* = 7 biologically independent mice per group. **i**, MRI analysis of fat and water suppressed images (left) and calculated fat fraction (right) in livers of WDR6-LKO mice and LC mice after HFD feeding for 16 weeks. *n* = 4 biologically independent mice per group. **j**, ^1^H magnetic resonance spectroscopy spectrum (^1^H-MRS) characteristics of liver in WDR6-LKO and LC mice. *n* = 4 biologically independent mice per group. Experiments in **a**–**j** were performed using male mice. Data in **a**, **d** and **f** are presented as the mean ± s.d., determined by two-way ANOVA and Tukey’s multiple-comparisons test. Data in **h**–**j** are presented as the mean ± s.d., determined by unpaired two-sided Student’s *t*-test. Source data

**Extended Data Fig. 2:**
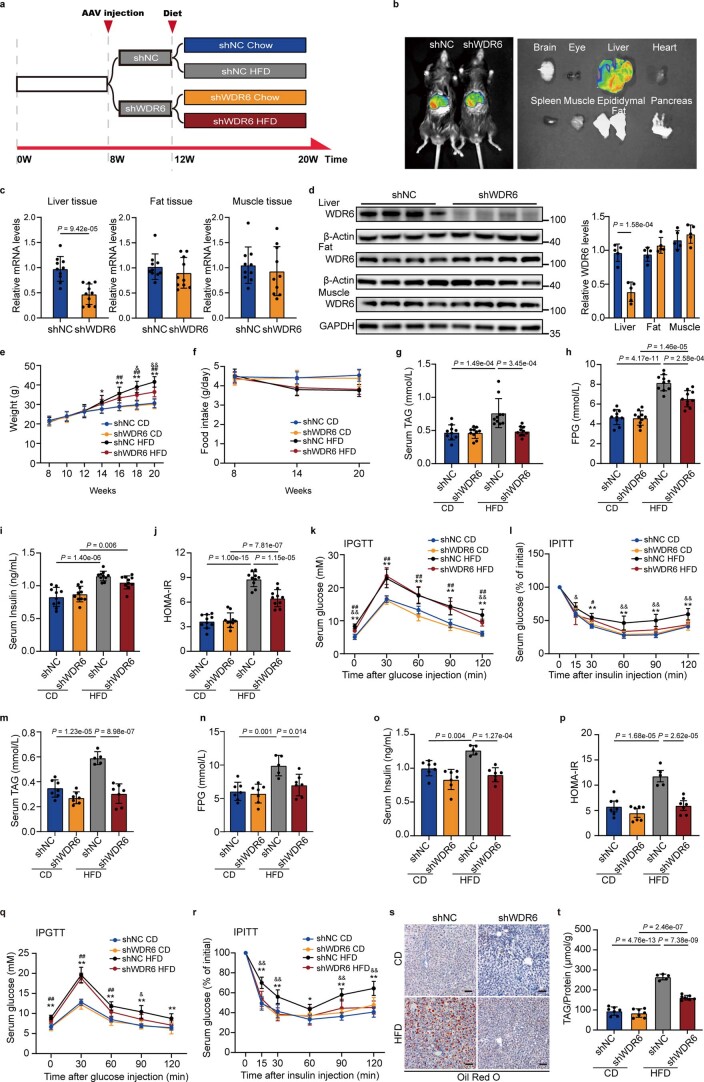
Liver-specific knockdown of *Wdr6* prevents HFD-induced IR, related to Fig. 2. **a**, Schematic diagram illustrating the experimental design for testing the effects of IR on *Wdr6* knockdown mice. 8-week-old C57BL/6J mice were injected with AAV2/8-TBG-shWDR6 (shWDR6 group) or AAV2/8-TBG-shNC (shNC group) via the tail vein. Four weeks after injection, each group was randomly sub-divided into CD or HFD group for additional 8-week feeding. **b**, Four weeks after AAV2/8-TBG-shNC or AAV2/8-TBG-shWDR6 injection, representative GFP expression was monitored by imaging *in vivo* and *in vitro*. **c**, RT-PCR analysis of *Wdr6* mRNA levels in liver, fat and muscle tissue. The expression of *Wdr6* was normalized to *Acta1* or *Actb* mRNA levels. **d**, Western blots of WDR6 protein levels in liver, fat and muscle tissue. β-Actin or GAPDH served as a loading control. **e-l**, Change curves of body weight, food intake, Serum TAG, FPG, insulin levels, HOMA-IR index, IPGTT and IPITT of male mice described in (**a**). **m-r** (♀), Serum TAG, FPG, insulin levels, HOMA-IR index, IPGTT and IPITT of female mice described in (**a**). **s** (♀), Representative ORO staining of female mice liver sections in the indicated group in (**a**). Scale bars, 50 μm. **t** (♀), Liver TAG levels of female mice described in **(a)**. For (**c**), (**g**-**l**), n = 10 biologically independent mice per group. (**d**), n = 5 biologically independent mice per group. (**e**-**f**), n = 8 biologically independent mice per group. (**m**-**r**), (**t**), n = 5 biologically independent mice in shNC HFD group, n = 7 biologically independent mice in other groups. Data in **(c), (d)** are presented as mean ± SD, determined by unpaired two-sided Student’s *t*-test, data in (**e-r**) and (**t**) as mean ± SD, determined by two-way ANOVA and Tukey’s multiple-comparisons test. In (**k**), (**l**), (**q**), (**r**), * indicates statistically significance between shNC HFD *vs*. shNC CD group, ^#^ indicates statistically significance between shWDR6 HFD *vs*. shWDR6 CD group, ^$^ indicates statistically significance between shWDR6 CD *vs*. shNC CD group. ^&^ indicates statistically significance between shWDR6 HFD *vs*. shNC HFD group. *, ^#^, ^$^, ^&^, *P* < 0.05; **, ^##^, ^$$^, ^&&^, *P* < 0.01. Source data

To test whether WDR6 contributes to IR-induced liver metabolic disorders, mice with liver selective *Wdr6* knockdown were engineered by adeno-associated virus (AAV)-containing *Wdr6* shRNA (HBAAV2/8-TBG-micro-mWdr6; shWDR6 mice). Control mice were injected with a control shRNA (shNC mice). Four weeks after injection, mice were fed a HFD for further 8 weeks (Extended Data Fig. 2a). We found that the liver tissue was indeed the main target of the AAV, as indicated by GFP labelling (Extended Data Fig. 2b). The *Wdr6* expression profile was also tested and liver *Wdr6* was effectively targeted (Extended Data Fig. 2c,d). Given food intake was held constant, the body weight curves were slightly decreased in shWDR6 than shNC mice (Extended Data Fig. 2e,f). Interestingly, after HFD feeding, the male shWDR6 mice protected from metabolic anomalies, including attenuated increases in serum levels of triacylglycerol (TAG) and fasting plasma glucose (FPG; Extended Data Fig. 2g,h). Insulin sensitivity was also improved, as assessed by serum insulin levels, homeostasis model assessment of insulin resistance (HOMA-IR), intraperitoneal (i.p.) glucose tolerance tests and i.p. insulin tolerance tests (Extended Data Fig. 2i–l). The phenotypes of female shWDR6 mice were similar to those of the males (Extended Data Fig. 2m–r). In liver, compared to the shNC group, male shWDR6 mice showed ameliorated liver lipid deposition, according to ultrasound analysis, Oil Red O (ORO) staining, transmission electron microscopy (TEM) and TAG contents (Fig. 2a–d). Similar phenotypes were also observed in female shWDR6 mice (Extended Data Fig. 2s,t). These data suggest that WDR6 is involved in liver lipid metabolism.

**Extended Data Fig. 3:**
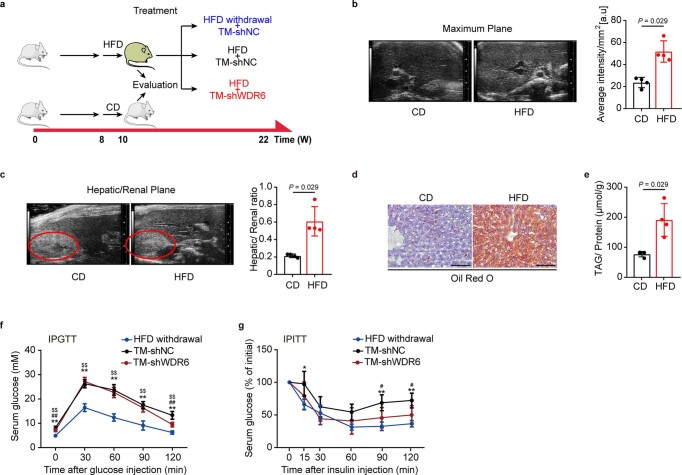
Liver-specific intervention of *Wdr6* can ameliorate hepatic steatosis and IR, related to Fig. 2. **a**, Schematic diagram of the experimental strategy to estimate the therapeutic effect of *Wdr6* knockdown on hepatic steatosis and insulin sensitivity. 8-week-old C57BL/6J mice were fed HFD/CD for 2 weeks. In HFD group, mice were separated into 3 subgroups: one group was injected with AAV2/8-TBG-shWDR6 (TM-shWDR6 group (red), the other two groups were injected with AAV2/8-TBG-shNC (HFD withdrawal group was switched to normal diet (blue), TM-shNC group were still fed HFD (black). Mouse icons produced using Servier Medical Art by Servier (https://smart.servier.com/) under a Creative Commons Attribution 3.0 Unported License (https://creativecommons.org/licenses/by/3.0/). **b**, Liver B-Mode ultrasound imaging of the maximum plane of the mice after CD or HFD consumption for 2 weeks. **c**, Liver B-Mode ultrasound imaging of the hepatic/renal plane of the mice after CD or HFD consumption for 2 weeks. The area circled in red represents the kidney. **d**, Representative ORO staining of liver sections of the mice after CD or HFD consumption for 2 weeks. Scale bars, 50 μm. **e**, Liver TAG levels of the mice after CD or HFD feeding for 2 weeks, the amount being normalized to the protein content of the same sample. **f**, **g**, IPGTT and IPITT of mice described in **(a)**. For (**b**-**e**), n = 4 biologically independent mice per group. (**f**, **g**), n = 4 biologically independent mice in HFD withdrawal group. n = 6 biologically independent mice in other groups. Data in **(b), (c)** and **(e)** are presented as mean ± SD, determined by unpaired two-sided Mann-Whitney test, data in (**f**), (**g**) are presented as mean ± SD, determined by two-way ANOVA and Tukey’s multiple-comparisons test. * indicates statistically significance between TM-shNC *vs*. HFD withdrawal group, ^#^ indicates statistically significance between TM-shWDR6 *vs*. TM-shNC group, ^$^ indicates statistically significance between TM-shWDR6 *vs*. HFD withdrawal group. *, ^#^, ^$^, *P* < 0.05, **, ^##^, ^$$^, *P* < 0.01. Source data

To make clear whether targeting liver WDR6 could ameliorate the adverse effects of a HFD, a therapeutic model (TM) was developed by injecting AAV-shWDR6 into 10-week-old mice that had already been fed a 2-week HFD (as AAV-mediated expression takes ~3–5 weeks to work, the injections began 2 weeks after HFD initiation), to mimic the therapeutic effect of targeting WDR6 (TM-shWDR6 group). The control mice (2-week HFD with AAV-shNC injection) were subdivided into two subgroups, one kept on a HFD regimen after AAV-shNC injection (TM-shNC group), while the other group was switched to chow diet (CD; HFD withdrawal group; Extended Data Fig. 3a). Compared with the TM-shNC group, HFD-induced IR and hepatic lipid deposition were alleviated in the TM-shWDR6 group (Fig. 2e,f and Extended Data Fig. 3b–g).

**Extended Data Fig. 4:**
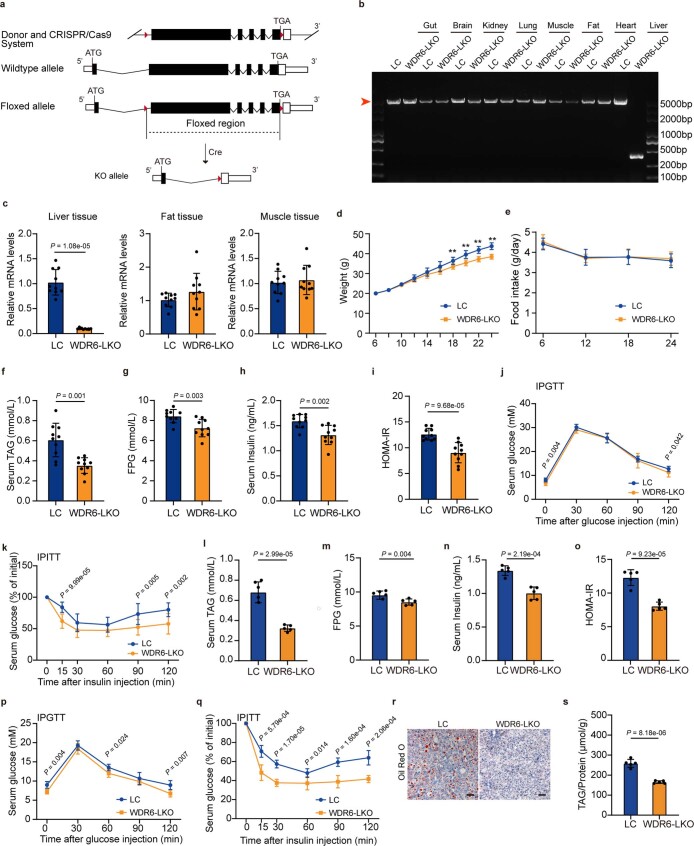
Liver-specific knockout of *Wdr6* prevents HFD-induced IR, related to Fig. 2. 8-week-old WDR6-LKO and LC mice were fed HFD for 16 weeks. **a**, Schematic diagram illustrating the building strategy of WDR6-LKO mice. **b**, PCR analysis revealing *Wdr6* fragments generated from the different tissues of WDR6-LKO mice, red arrow indicates the PCR fragment of wildtype *Wdr6* allele. **c**, RT-PCR analysis of *Wdr6* mRNA levels in liver, fat and muscle tissue. The expression of *Wdr6* was normalized to *Acta1* or *Actb* mRNA levels. n = 10 biologically independent mice per group. **d-e**, Change curves of body weight, food intake. n = 6 biologically independent mice per group. **f-k**, Serum TAG, FPG, insulin levels and HOMA-IR index and IPGTT and IPITT of male WDR6-LKO mice and control, n = 10 biologically independent mice per group. **l-o** (♀), Serum TAG, FPG, insulin levels and HOMA-IR index of female WDR6-LKO mice and control, n = 5 biologically independent mice per group. **p**,**q** (♀), IPGTT and IPITT of female WDR6-LKO mice and control, n = 6 biologically independent mice in LC group, n = 5 biologically independent mice in WDR6-LKO group. **r** (♀), Representative ORO staining of female mice liver sections of female WDR6-LKO mice and control. Scale bars, 50 μm. **s** (♀), Liver TAG level of female WDR6-LKO mice and control. n = 5 biologically independent mice per group. Data in **(c)** and **(f)** are presented as mean ± SD, determined by unpaired two-sided Mann-Whitney test. Data in **(d-e), (g-q)** and **(s)** are presented as mean ± SD, determined by two-sided Student’s *t*-test. * *P* < 0.05, ** *P* < 0.01. Source data

Liver-specific knockout *Wdr6* (WDR6-LKO) mice were also created through CRISPR–Cas9 technology (Extended Data Fig. 4a–c). Given food intake was held constant, the body weight curves showed a slightly decreased body weight in WDR6-LKO than wild-type (WT) littermate control (LC) mice (Extended Data Fig. 4d,e). Compared to LC, male WDR6-LKO mice showed decreased serum TAG and FPG levels (Extended Data Fig. 4f,g), and insulin sensitivity was also improved (Extended Data Fig. 4h–k). In the liver, TAG deposition decreased (Fig. 2g,h), and a lower degree of fatty liver was also observed by magnetic resonance imaging (MRI; Fig. 2i,j). Similar phenotypes also appeared in female WDR6-LKO mice (Extended Data Fig. 4l–s). These results demonstrate that targeting *Wdr6* in the liver can reduce HFD-associated liver lipid deposition.

**Extended Data Fig. 5:**
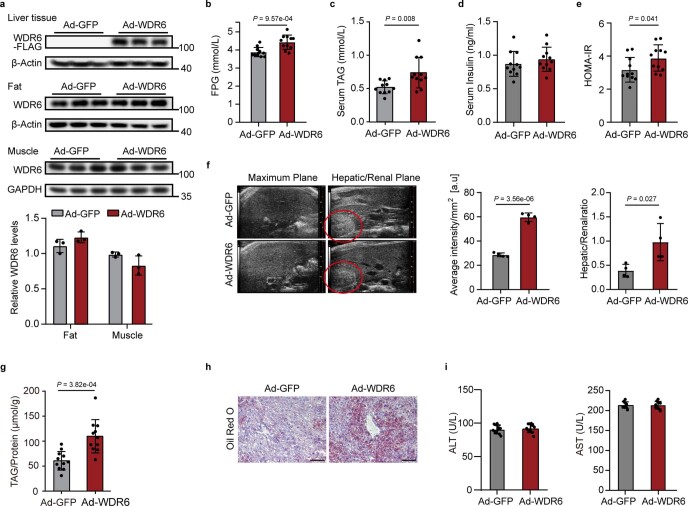
Over-expression of *Wdr6* increases liver TAG contents, related to Fig. 2. 8-week-old male C57BL/6J mice were injected Ad-WDR6 or Ad-GFP three times with 5-day intervals via tail vein. **a**, Western blots of WDR6 protein levels of liver, fat and muscle tissue. β-Actin or GAPDH served as a loading control. **b-e**, Serum FPG levels, TAG levels, serum insulin levels and HOMA-IR index. **f**, Liver B-Mode ultrasound imaging (left, the area circled in red represents the kidney), calculated average intensity (middle) and hepatic/ renal ratio (right). **g**, TAG contents in liver tissue, the concentration was normalized to the protein content of the same sample. **h**, Representative ORO staining of liver sections. Scale bars, 100 μm. **i**, Serum ALT and AST levels of each group. For (**a**), n = 3 biologically independent mice per group. (**b**-**e**), (**g**), (**i**), n = 11 biologically independent mice per group. (**f**), n = 4 biologically independent mice per group. Data in **(a-b), (e-g)** and **(i)** are presented as mean ± SD, determined by two-sided Student’s *t*-test. Data in **(c)** are presented as mean ± SD, determined by unpaired two-sided Mann-Whitney test. * *P* < 0.05, ** *P* < 0.01. Source data

To test the role of WDR6 from a gain-of-function point of view, adenovirus-mediated *Wdr6*-overexpressing mice (Ad-WDR6) were generated (Extended Data Fig. 5a). Compared with the WT controls (Ad-GFP), Ad-WDR6 mice showed increased serum FPG/TAG levels and worsened HOMA-IR but without a significant change in insulin levels (Extended Data Fig. 5b–e). Higher levels of liver TAG and lipid deposition were also observed (Extended Data Fig. 5f–h). Liver injury was not present, as judged by hepatic enzyme levels (Extended Data Fig. 5i). These results imply that the transient increase of *Wdr6* expression promotes hepatic liver fat deposition.

Taken together, these data show that liver-specific inhibition of *Wdr6* ameliorates, or reverses, metabolic disorders during IR, especially liver lipid deposition, while *Wdr6* overexpression has the opposite effects.

### Liver WDR6 affects liver triacylglycerol deposition by regulating de novo lipogenesis

**Fig. 3:**
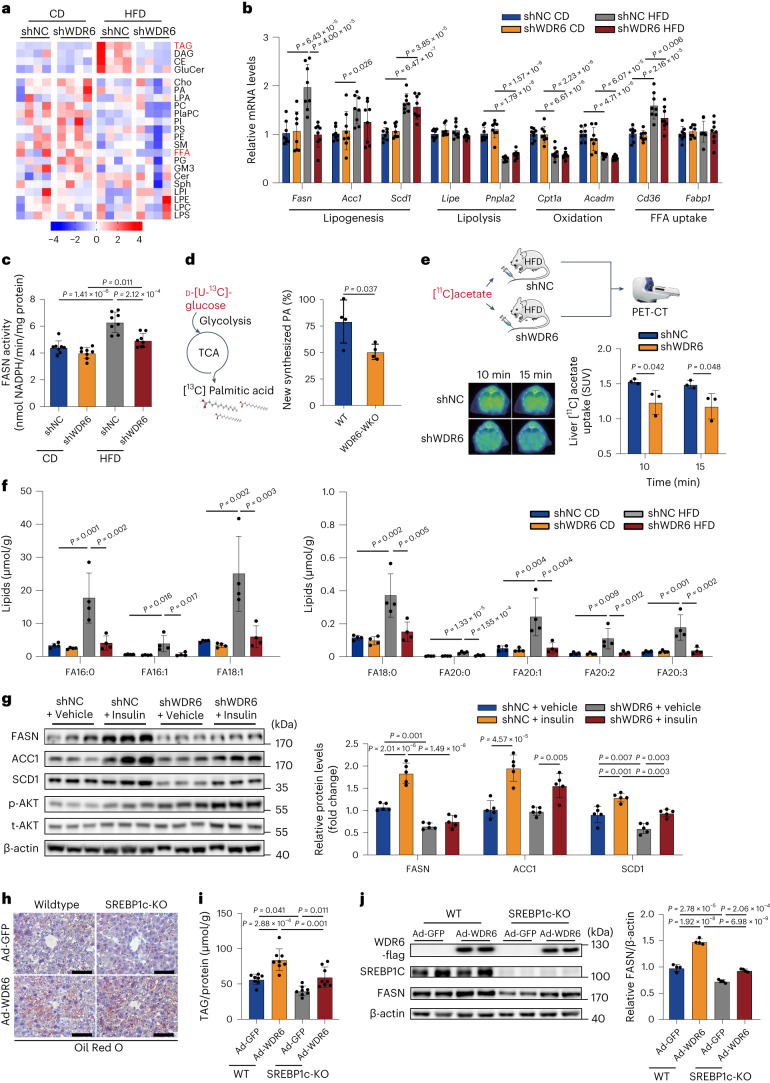
Liver WDR6 affects triacylglycerol deposition through regulation of de novo lipogenesis. **a**, Heat map of lipid species in livers of AAV-shWDR6 and AAV-shNC mice after CD or HFD feeding for 8 weeks, as determined by lipidomic analysis. *n* = 4 biologically independent mice per group. **b**, RT–PCR of representative genes of TAG metabolism in livers of the indicated group. *n* = 8 biologically independent mice per group. *Actb* served as a normalization control. **c**, Liver FASN activity for indicated groups in **a**. *n* = 8 biologically independent mice per group. **d**, Analysis of production of [^13^C]palmitic acid (converted from d-[U-^13^C]-glucose) in primary hepatocytes of WDR6-WKO and WT mice. *n* = 4 biologically independent mice per group. **e**, PET-CT of shWDR6 and shNC mice. Schematic of the [^11^C]acetate uptake experiment, which is used to assess the levels of DNL (upper), representative [^11^C]acetate-PET imaging (lower left) and calculated liver [^11^C]acetate uptake standard uptake value (SUV; lower right) were obtained. *n* = 3 biologically independent mice per group. Mouse and syringe icons produced using Servier Medical Art by Servier (https://smart.servier.com/) under a Creative Commons Attribution 3.0 Unported Licence. Image of PET-CT scanner reproduced with permission from MITRO Biotech. **f**, Lipidomic analyses of the abundance of long-chain FA species in liver of shNC and shWDR6 mice under the CD and HFD states, respectively. *n* = 4 biologically independent mice per group. **g**, Western blots of DNL proteins in livers of HFD-fed shWDR6 and shNC mice, with insulin/vehicle treatment, respectively. *n* = 5 biologically independent mice per group. β-actin serves as the loading control. **h**, 12-week-old SREBP1c-KO and control mice were injected with Ad-WDR6 or Ad-GFP 15 d before euthanasia. Representative liver ORO staining was obtained. Scale bars, 50 μm. **i**, Liver TAG levels of the mice described in **h**. *n* = 8 biologically independent mice per group. **j**, FASN protein levels in liver of the mice described in **h**. *n* = 4 biologically independent mice per group. β-actin served as the loading control. Experiments in **a**–**j** were performed using male mice. Data in **b**, **c**, **f**, **g**, **i** and **j** are presented as the mean ± s.d., determined by two-way ANOVA and Tukey’s multiple-comparisons test. Data in **d** and **e** are presented as the mean ± s.d., determined by unpaired two-sided Student’s *t*-test. Source data

**Extended Data Fig. 6:**
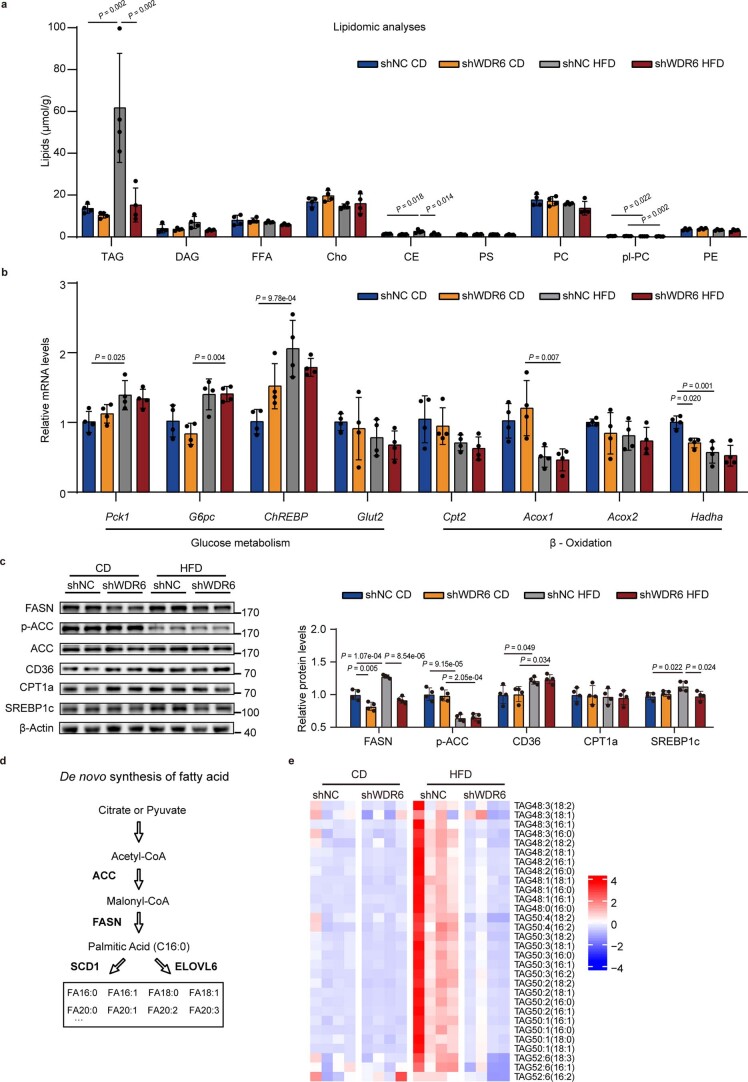
Liver WDR6 affects DNL related components, and the abundance of FFAs of TAG side chain, related to Fig. 3. **a**. Amount of lipid species in liver tissue of shNC and shWDR6 mice after feeding with CD or HFD, shown as μmol/g. **b**. qPCR of representative genes of liver glucose and TAG oxidation pathways of shNC and shWDR6 mice after feeding with the CD or HFD. *Actb* serves as a normalization control. **c**. Western blots of representative genes of liver TAG metabolism pathways of shNC and shWDR6 mice under the CD or HFD state. β-Actin serves as a loading control. **d**. A schematic diagram illustrating the *de novo* synthesis of fatty acids. **e**. Heat map of fatty acids species of TAG in liver tissues of shNC and shWDR6 mice after feeding with CD or HFD. For (**a**-**c**), (**e**) n = 4 biologically independent mice per group. Data in **(a-c)** are presented as mean ± SD, determined by two-way ANOVA and Tukey’s multiple-comparisons test. * *P* < 0.05, ** *P* < 0.01. Source data

We then performed lipidomic analyses of livers from shWDR6 and shNC mice in the CD-fed and HFD-fed groups, respectively, to gain insight to the lipid metabolic pathways that may be affected by WDR6. As shown in Fig. 3a and Extended Data Fig. 6a, the top metabolite with the greatest differences among the HFD-fed groups was TAG. We thus assessed the expression patterns of various representative genes involved in the main pathways of liver TAG metabolism, including DNL, lipolysis, free fatty acid oxidation and uptake, as well as certain representative genes of glucose metabolism, in shWDR6 and shNC mice. Among these, the DNL-related gene *Fasn* was lower in shWDR6 mice compared to shNC mice in HFD groups, both at the mRNA and protein levels. No obvious difference in transcription of glucose metabolic genes between groups was observed (Fig. 3b and Extended Data Fig. 6b,c). Moreover, in HFD groups, FASN enzymatic activity was also lower after *Wdr6* knockdown compared to control mice (Fig. 3c).

To further confirm our findings, carbon isotope labelling analysis was performed to estimate the content of newly synthesized palmitate, the product of FASN, in primary hepatocytes from *Wdr6* whole-body knockout mice (WDR6-WKO) and WT controls. ^13^C-labelled palmitate from tracker d-[uniformly labelled ^13^C (U-^13^C)]-glucose was markedly lower in WDR6-WKO hepatocytes compared to that in WT (Fig. 3d). Moreover, Liver DNL rate was estimated by using [^11^C]acetate positron emission tomography (PET)^26^, and it was reduced due to hepatic *Wdr6* knockdown (Fig. 3e). Additionally, as the side chains of TAG are composed of various kinds of FAs, depending on different biosynthetic enzyme involved (Extended Data Fig. 6d), we found the FAs with the most obvious differences were saturated (FA16:0, FA18:0) and monounsaturated (FA16:1, FA18:1), which are the direct or proximal products of FASN or SCD1, respectively^27,28^ (Fig. 3f and Extended Data Fig. 6e). Taken together, these results imply that WDR6 promotes hepatic DNL during IR, and among the genes involved, *Fasn* might be a primary target.

We next tested whether WDR6 could mediate the process of insulin regulating FASN during IR. Thus, HFD-shWDR6 mice were injected with insulin and euthanized after 6 h. Unlike in HFD-shNC mice, knockdown of *Wdr6* markedly attenuated the ability of insulin to increase FASN levels but with minimal effect on ACC1 or SCD1 (Fig. 3g), which indicated that during IR, upregulation of WDR6 may serve as an alternative means by which insulin increases FASN levels and DNL.

**Extended Data Fig. 7:**
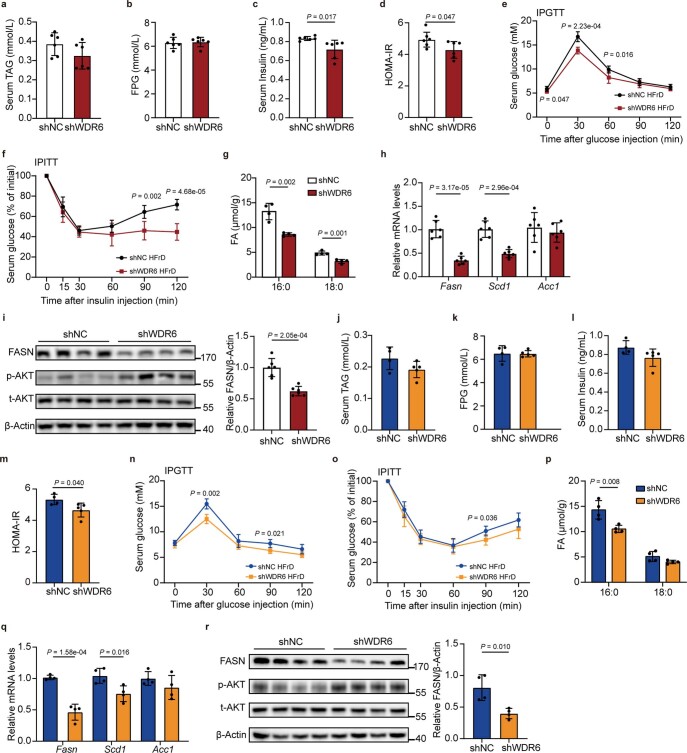
Liver-specific intervention of *Wdr6* can ameliorate hepatic DNL in high fructose diet feeding mice, related to Fig. 3. **a-d**, Serum TAG, FPG, insulin levels and HOMA-IR index of male HFrD feeding shWDR6 mice or HFrD feeding shNC mice (control). **e,f**, IPGTT and IPITT of male shWDR6 mice with HFrD or control. **g**, Abundance of long-chain fatty acid species (FA16:0, FA 18:0) in liver of male shWDR6 mice with HFrD or control, respectively. **h**, qPCR of hepatic DNL genes in livers of male shWDR6 mice with HFrD or control, respectively. **i**, Western blots of FASN in liver of male shWDR6 mice with HFrD or control, respectively. **j-m** (♀), Serum TAG, FPG, insulin levels and HOMA-IR index of female HFrD fed shWDR6 mice or HFrD fed shNC mice (control). **n,o** (♀), IPGTT and IPITT of female shWDR6 mice with HFrD or control. **p** (♀), Abundance of long-chain fatty acid species (FA16:0, FA18:0) in livers of female shWDR6 mice with HFrD or control, respectively. **q** (♀), qPCR of hepatic DNL genes in livers of female shWDR6 mice with HFrD or control, respectively. **r** (♀), Western blots of FASN in livers of female shWDR6 mice with HFrD or control, respectively. For (**a**-**f**), (**h**, **i**), n = 6 biologically independent mice per group. (**g**), (**p**-**r**), n = 4 biologically independent mice per group. (**j**-**o**), n = 4 biologically independent mice in shNC group, n = 5 biologically independent mice in shWDR6 group. Data in **(a)-(r)** are presented as mean ± SD, determined by unpaired two-sided Student’s *t*-test. * *P* < 0.05, ** *P* < 0.01. Source data

To validate the relationship of WDR6 and hepatic DNL, we built an AAV-shWDR6 model in mice with a high-fructose diet (HFrD), which is more commonly used in hepatic DNL studies, as fructose is a strong substrate for hepatic DNL^7,29–32^. Compared to HFrD-shNC, HFrD-shWDR6 showed improved insulin sensitivity, while the serum TAG/FPG levels did not obviously change (Extended Data Fig. 7a–f). Hepatic saturated FAs, the product of DNL, were lower (Extended Data Fig. 7g), and the hepatic FASN level decreased (Extended Data Fig. 7h,i). Similar tendencies were also observed in female mice (Extended Data Fig. 7j–r). These data confirm that WDR6 plays a crucial role in regulating hepatic DNL.

Next, we wondered whether WDR6-mediated regulation of *Fasn* expression depends on SREBP1c, the key transcription factor of DNL^7^. We overexpressed WDR6 on the background of SREBP1c-KO C57BL6 mice. Although SREBP1c deletion can reduce liver TAG deposition (Fig. 3h,i), similar to previously reported findings^33^, WDR6 overexpression could still enhance the liver TAG and FASN protein levels in SREBP1c-KO mice (Fig. 3h–j). These results demonstrate that SREBP1c is important but not essential for the effect of WDR6-mediated regulation of *Fasn* expression and liver TAG deposition, as deleting SREBP1c only partially blocks the effects.

### WDR6 interacts with and dephosphorylates PPP1CB at Thr316

**Fig. 4:**
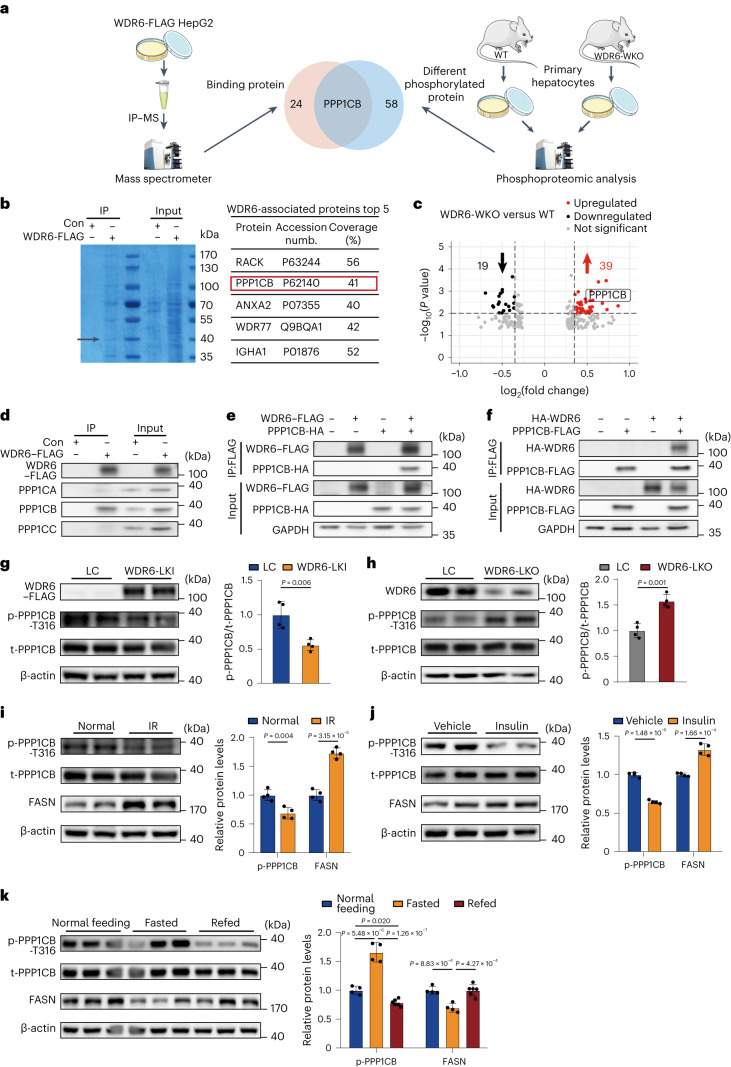
WDR6 interacts with PPP1CB to facilitate the latter’s dephosphorylation at Thr316. **a**, Schematic of immunoprecipitation using mass spectrometry (IP–MS) to identify proteins associated with WDR6 in WDR6–FLAG cells (left) and phosphoproteomic analysis to identify differential phosphorylated proteins in primary hepatocytes of WT and WDR6-WKO mice, respectively (right). Petri dish, mouse and microtube icons produced using Servier Medical Art by Servier (https://smart.servier.com/) under a Creative Commons Attribution 3.0 Unported Licence. Image of mass spectrometer reproduced with permission from Jingjie PTM BioLab. **b**, Eluted fraction from IP indicated in **a** was analysed by SDS–PAGE with Coomassie Brilliant blue staining (left), and top five of the WDR6-associated proteins identified in MS are listed (right). The black arrow represents the position of PPP1CB. **c**, A volcano plot of differential proteins shown in phosphoproteomic analysis of primary hepatocytes isolated from WT and WDR6-WKO mice. There were 19 downregulated proteins (black) and 39 upregulated proteins (red). **d**, Representative IP analysis of the interaction of endogenous WDR6–FLAG fusion protein with endogenous PPP1CA, PPP1CB or PPP1CC in HepG2 cells. **e**,**f**, Representative IP analysis of the interaction of WDR6–FLAG and PPP1CB-HA (**e**) and PPP1CB-FLAG and HA-WDR6 (**f**) in HEK293 cells. GAPDH served as the loading control. **g**,**h**, Western blots of phospho-PPP1CB (Thr316) in liver of WDR6-LKI (**g**) and WDR6-LKO (**h**) mice. *n* = 4 biologically independent mice per group. β-actin served as the loading control. **i**–**k**, Western blots of phospho-PPP1CB (Thr316) and FASN levels in liver in response to HFD-induced IR state (*n* = 4 biologically independent mice per group) (**i**), insulin stimulation (0.75 units per kg body weight, i.p.) for 6 h (*n* = 4 biologically independent mice per group) (**j**) and normal feeding, overnight fasting (*n* = 4 biologically independent mice) and 6 h of refeeding (*n* = 6 biologically independent mice) (**k**). β-actin served as the loading control. Experiments in **c** and **g**–**k** were performed using male mice. Data in **g**–**j** are presented as the mean ± s.d., determined by unpaired two-sided Student’s *t*-test. Data in **k** are presented as the mean ± s.d., determined by one-way ANOVA and Tukey’s multiple-comparisons test. Source data

**Extended Data Fig. 8:**
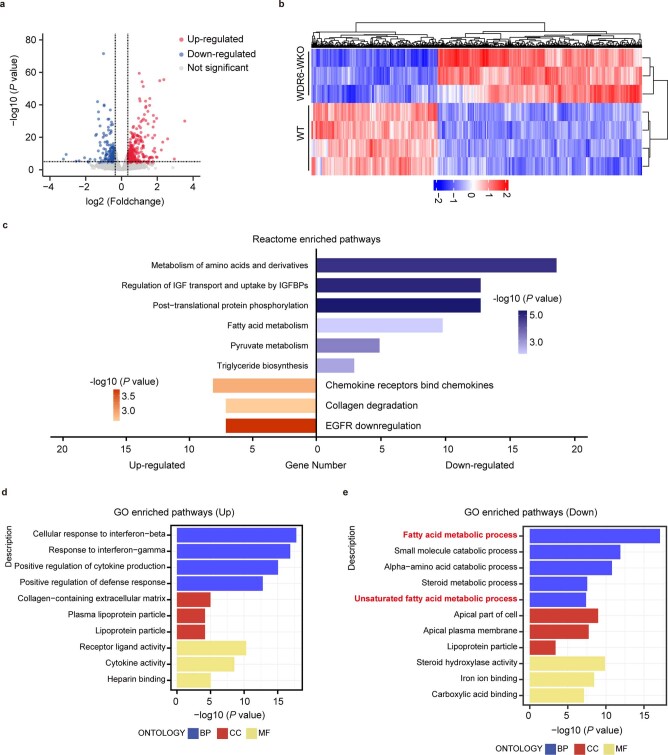
Transcriptome analysis of primary hepatocytes from WT and WDR6-WKO mice, related to Fig. 3. **a**, A volcano plot showing differentially expressed genes in WDR6-WKO *vs*. WT groups. Red, upregulated genes; blue, downregulated genes, gray, no significant change. **b**, Heat map of differentially expressed genes in WDR6-WKO vs. WT groups. n = 3 biologically independent mice per group. **c**, Reactome enrichment analysis of differentially expressed genes in WDR6-WKO *vs*. WT groups. **d**, GO enrichment analysis of up-regulated genes in WDR6-WKO *vs*. WT groups. **e**, GO enrichment analysis of down-regulated genes in WDR6-WKO *vs*. WT groups.

We continued to explore the mechanisms by which WDR6 regulates FASN expression. Transcriptomic analyses of primary hepatocytes from WDR6-WKO and control mice were performed (Extended Data Fig. 8a,b). Gene Ontology (GO) and Reactome analyses revealed that the enriched DEGs were involved not only in FA metabolism and TAG biosynthesis, but also in post-translational protein phosphorylation pathways (Extended Data Fig. 8c–e), suggesting that WDR6 might participate in post-translational regulatory processes. Along these lines, we chose to perform FLAG affinity immunoprecipitation (IP) of WDR6–FLAG cells (Extended Data Fig. 1) to identify binding partners for WDR6 (Fig. 4a). The predominant bands observed by SDS–PAGE were selected for identification by mass spectrometry (MS; Fig. 4b).

In parallel with the IP–MS analysis, we also performed phosphoproteomic analysis of primary hepatocytes from both WDR6-WKO and WT mice (Fig. 4a and Supplementary Data Fig. 2). Proteomic analysis was performed as an internal control (Supplementary Data Fig. 3). An unreported phosphorylation site in the beta-type catalytic subunit of serine/threonine-protein phosphatase 1 (PP1; PPP1CB), phospho-Thr316, was discovered to be increased in WDR6-WKO primary hepatocytes, compared with controls (Fig. 4c). Further, PPP1CB was also found in the WDR6–FLAG IP elution sample identifying it as a binding partner of WDR6 (Fig. 4b). Thus, PPP1CB appeared in the two distinct datasets (Fig.4a). These results led us to hypothesize that *Wdr6* deficiency is associated with increased phospho-PPP1CB (Thr316) perhaps because WDR6 is a direct or indirect partner for PPP1CB.

To further validate the interaction of WDR6 and PPP1CB, IP analysis was performed from extracts of WDR6–FLAG cells. We found that endogenous WDR6 interacted with PPP1CB, but no obvious interaction with PPP1CA or PPP1CC, the other two catalytic isoforms of PP1 (refs. ^34,35^; Fig. 4d). In addition, recombinant WDR6–FLAG and PPP1CB-HA, or recombinant HA-WDR6 and PPP1CB–FLAG were co-overexpressed in HEK293 cells in order to perform reciprocal FLAG affinity IP analysis. We found that in both scenarios there was a (direct or indirect) physical interaction between WDR6 and PPP1CB (Fig. 4e,f).

Next, we investigated whether phospho-PPP1CB (Thr316) was affected by WDR6. The level of phospho-PPP1CB (Thr316) was examined in liver samples of *Wdr6* liver-specific knock-in (WDR6-LKI; Supplementary Data 4) and WDR6-LKO mice, using custom primary antibody that specifically recognizes the phospho-PPP1CB at Thr316. Compared to the corresponding WT littermates, liver phospho-PPP1CB (Thr316) levels were lower in WDR6-LKI mice (Fig. 4g) and, conversely, higher in WDR6-LKO mice (Fig. 4h). The responses of phospho-PPP1CB (Thr316) to exogenous/endogenous insulin were also tested. We found that phospho-PPP1CB (Thr316) levels were lower in HFD-induced IR mice, compared to normal controls (Fig. 4i), and lower in IR mice with 6 h of insulin treatment (0.75 U per kg body weight), compared to IR control mice (Fig. 4j). Moreover, phospho-PPP1CB (Thr316) levels were increased after 16 h of fasting (when insulin levels are low), compared to normal feeding, and decreased dramatically in response to 6 h of refeeding (when insulin levels rise; Fig. 4k). The FASN levels in those above treatments showed corresponding changes as expected. These results demonstrated that the phospho-PPP1CB (Thr316) is affected by WDR6, as well as insulin and external nutritional circumstances.

### PPP1CB-Thr316 mediates WDR6 regulating *Fasn* transcription

**Fig. 5:**
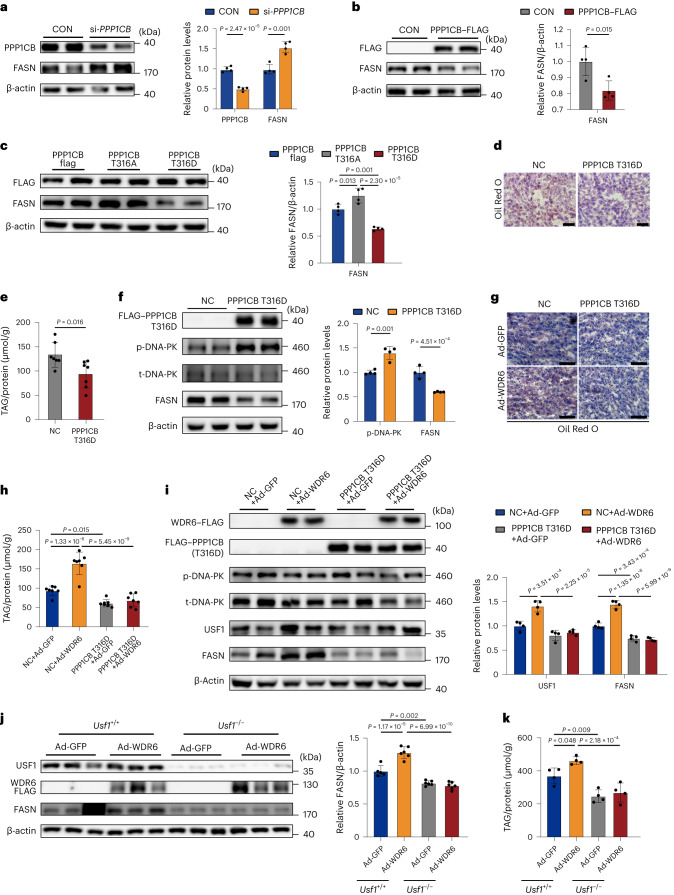
WDR6 regulates *FASN* transcription by promoting dephosphorylation of PPP1CB. **a**,**b**, Western blots of FASN levels in HepG2 cells transfected with *PPP1CB* siRNA (**a**) or *PPP1CB* overexpression vector (**b**). β-actin served as the loading control. **c**, FASN protein levels of lysates from cells expressing PPP1CB–FLAG, Thr316Ala–FLAG or Thr316Asp–FLAG. β-actin served as the loading control. **d**, Representative ORO staining of liver sections from mice injected with AAV2/8-PPP1CBb Thr316Asp (PPP1CB-Thr316Asp group) or AAV2/8-GFP (NC group). Scale bars, 50 μm. **e**, Liver TAG levels of mice in PPP1CB-Thr316Asp group or NC group; the amount was normalized to the protein content of the same sample, *n* = 7 biologically independent mice per group. **f**, Western blots of phospho-DNA-PK and total-DNA-PK and FASN protein levels in liver of PPP1CB-Thr316Asp or NC group. *n* = 4 biologically independent mice per group. β-actin served as a loading control. **g**, Representative ORO staining of liver sections of PPP1CB-Thr316Asp or NC group randomly injected with Ad-WDR6 or Ad-GFP. Scale bars, 50 μm. **h**, Liver TAG levels of the mice described in **g**; the amount was normalized to the protein content of the same sample, *n* = 7 biologically independent mice per group. **i**, Phospho-DNA-PK and total-DNA-PK, USF1 and FASN protein levels in liver of the mice described in **g**. *n* = 4 biologically independent mice per group. β-actin served as the loading control. **j**, Western blots of FASN protein levels in primary hepatocytes of *Usf1*^−/−^ or *Usf1*^+/+^ (WT) mice injected with Ad-WDR6 or Ad-GFP, respectively. β-actin served as the loading control. **k**, TAG levels of the primary hepatocytes described in **j**; the amount was normalized to the protein content. For **a**–**c** and **k**, *n* = 4 independent experiments; for **j**, *n* = 5 independent experiments in *Usf1*^+/+^, Ad-GFP group; *n* = 6 independent experiments in other groups. Experiments in **e**–**k** were performed using male mice. Data in **a**, **b**, **e** and **f** are presented as the the mean ± s.d., determined by unpaired two-sided Student’s *t*-test. Data in **c** are presented as the mean ± s.d., determined by one-way ANOVA and Tukey’s multiple-comparisons test. Data in **h**–**k** are presented as the mean ± s.d., determined by two-way ANOVA and Tukey’s multiple-comparisons test. Source data

To clarify the physiological function of PPP1CB in hepatic DNL, HepG2 cells were transfected with si-*PPP1CB* or PPP1CB–FLAG overexpression vectors. We observed a negative correlation between the levels of PPP1CB and FASN (Fig. 5a,b). Additionally, we constructed overexpression vectors of mutant PPP1CB in which Thr316 was replaced with either alanine (PPP1CB-Thr316Ala) or aspartic acid (PPP1CB-Thr316Asp) to mimic the dephosphorylated or hyperphosphorylated forms of the protein, respectively. Consistent with the data above, we found that FASN levels were elevated after overexpression of PPP1CB-Thr316Ala and, conversely, decreased upon overexpression of PPP1CB-Thr316Asp (Fig. 5c).

We then performed an in vivo study to assess the effect of the PPP1CB mutants. Mammalian PP1 is reported to regulate liver lipogenesis through DNA-dependent protein kinase (DNA-PK) and upstream stimulatory factor 1 (USF1), a key transcriptional factor for Fasn^36^. DNA-PK is dephosphorylated by PP1 and increases the transcriptional activation of lipogenic genes during insulin treatment^37^, so we wondered whether it could act as a downstream molecule of PPP1CB. We performed AAV-mediated liver overexpression of PPP1CB-Thr316Asp (PPP1CB-Thr316Asp mice). This resulted in lower liver lipid deposition and TAG levels, compared to control-treated mice and was accompanied by increased phospho-DNA-PK and lower FASN levels (Fig. 5d–f). On this basis, we subsequently overexpressed WDR6 to assess whether WDR6 could still enhance DNL when PPP1CB was mutated. Similarly to the results above, increased WDR6 expression aggravated liver lipid deposition in the control group and increased FASN levels; however, these differences were diminished in the PPP1CB-Thr316Asp mice (Fig. 5g–i). Additionally, we found that USF1, a downstream target of PP1/DNA-PK, increased following overexpression of WDR6, while the response was smaller when PPP1CB-Thr316Asp was overexpressed. These data indicate that phospho-PPP1CB (Thr316) mediates WDR6 regulating USF1 and FASN^31^. Because SREBP1c is not an essential mediator of WDR6-induced regulation of lipogenesis (Fig. 3h–j), we hypothesized that USF1 might play this role. Primary hepatocytes from *Usf1*^−/−^ mice and WT littermates were isolated and transfected with the WDR6-overexpressing adenovirus (*Usf1*^−/− ^+ Ad-WDR6) or a negative control (*Usf1*^−/− ^+ Ad-GFP). WDR6 was unable to drive lipogenesis and cellular TAG deposition without *Usf1* (Fig. 5j,k).

### WDR6 interacts with PPP1CB through a FKSRSR motif

**Fig. 6:**
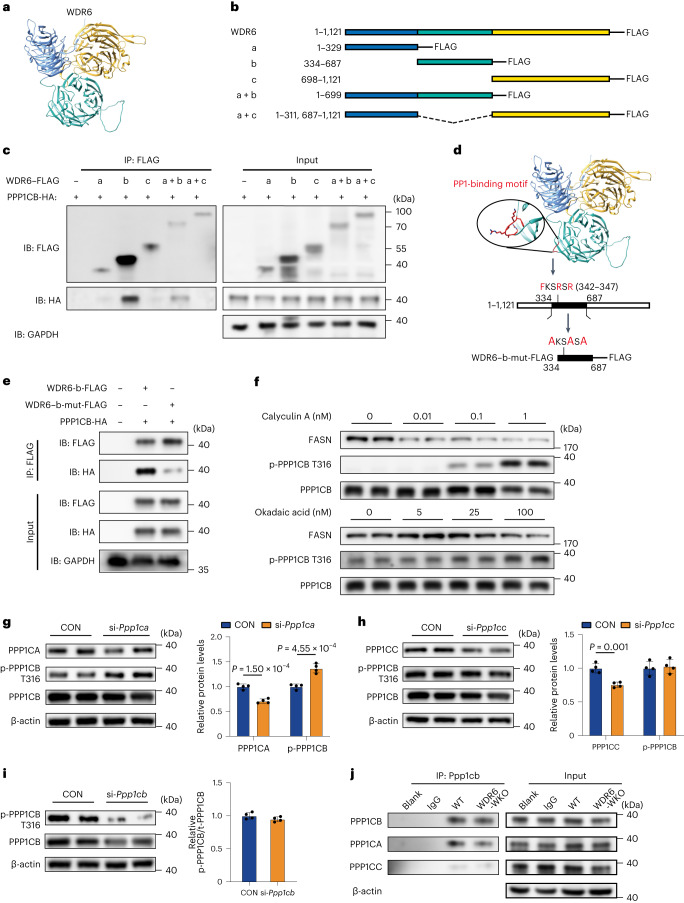
An FKSRSR motif of WDR6 interacts with PPP1CB, while PPP1CA is involved in the dephosphorylation of PPP1CB. **a**, The protein structure of WDR6 predicted by the AlphaFold. **b**, The truncated WDR6 variants. **c**, Representative IP analysis of the interaction of truncated WDR6 proteins and PPP1CB in lysates of HEK293 cells. GAPDH served as the loading control. **d**, The corresponding location of the putative PP1-binding motif FKSRSR on WDR6 protein and the design of the WDR6–b-mut-FLAG variant. **e**, Representative IP analysis of WDR6–b-mut-FLAG and PPP1CB expressed in HEK293 cells. GAPDH served as a loading control. **f**, Representative western blots to evaluate the dose-dependent effects of treatment for 24 h with calyculin A or okadaic acid on the phospho-PPP1CB (Thr316) and FASN protein levels. **g**–**i**, Western blots for phospho-PPP1CB (Thr316) in mouse primary hepatocytes transfected with *Ppp1ca* siRNA (**g**), *Ppp1cb* siRNA (**h**) or *Ppp1cc* siRNA (**i**), as indicated. *n* = 4 independent experiments. β-actin served as the loading control. **j**, Representative IP analysis of the interaction of PPP1CB with PPP1CA, and PPP1CB with PPP1CC, in primary hepatocytes of WT and WDR6-WKO mice, respectively. β-actin served as the loading control. Data in **g**–**i** are presented as the mean ± s.d., determined by unpaired two-sided Student’s *t*-test. Source data

To explore the mechanism by which WDR6 interacts with PPP1CB, the structure of the WDR6 protein was predicted using AlphaFold Protein Structure Database based on homology modelling. It was predicted that WDR6 has three domains with highly conserved β-barrel topology (Fig. 6a). Based on the predicted structure, a series of truncated WDR6–FLAG variants were designed (Fig. 6b), and each was co-overexpressed together with HA-tagged PPP1CB in HEK293 cells. FLAG-based IP analysis showed that PPP1CB-HA was detected in the eluted samples from cells harbouring either the b variant (334–687 amino acids) or the a + b variant of WDR6 (1–699 amino acids; Fig. 6c), suggesting that WDR6 binds PPP1CB through the b domain. Notably, we found a putative PP1-binding motif ‘FKSRSR’ (F-x-x-K/R-x-K/R, where x can be any residue^38^) located in the b variant (342–347 amino acids; Fig. 6d). The motif was first discovered in B cell lymphoma 2 (BCL-2) and was recognized as a PPP1C-dependent apoptotic signature^38^. We subsequently mutated the conserved amino acids of the motif to alanine in the b variant (b-mut-FLAG) to determine whether this domain is required for the binding to PPP1CB (Fig. 6d). IP analysis showed that the b-mut-FLAG version of WDR6 cannot bind PPP1CB (Fig. 6e).

To the best of our knowledge, WD40 domains are responsible for binding with other proteins; thus, we speculated that WDR6 might bring about the dephosphorylation of phospho-PPP1CB (Thr316) by acting as a scaffold protein for certain phosphatase. To date, the Ser/Thr protein phosphatases are mainly distributed in the PP1 and PP2A family^39,40^. Thus, we treated HepG2 cells with several inhibitors specific to various phosphatases, especially within the PP1 and PP2A family. Calyculin A, an inhibitor of PP1 and PP2A^41^, increased the level of phospho-PPP1CB (Thr316) in a dose-dependent manner (Fig. 6f). Okadaic acid is also an inhibitor of PP1 (half-maximal inhibitory concentration (IC_50_) = 10 nM) and PP2A (IC_50_ = 0.1 nM), with complete inhibition of PP2A activity at 1–2 nM, whereas higher concentrations are required to inhibit PP1 activity^42–44^. The levels of phospho-PPP1CB (Thr316) showed only a modest increase when cells were treated with 5 nM or 25 nM okadaic acid, under which circumstances PP2A activity is blunted but PP1 remains active. However, an increase in phospho-PPP1CB (Thr316) levels was more evident at 100 nM okadaic acid, a concentration at which PP1 activity is inhibited (Fig. 6f).

Next, we determined the levels of phospho-PPP1CB (Thr316) in mouse primary hepatocytes transfected with siRNAs targeting *Ppp1ca*, *Ppp1cb* or *Ppp1cc*. The ratio of phospho-PPP1CB (Thr316) to total PPP1CB was higher in *Ppp1ca*-deficient cells than in *Ppp1cb*-deficient or *Ppp1cc*-deficient cells (Fig. 6g–i). These results indicate that PPP1CA might be involved in the dephosphorylation of PPP1CB (Thr316). IP was therefore performed for PPP1CB in primary hepatocytes of WDR6-WKO mice and their WT counterparts. We found that PPP1CB could bind PPP1CA in WT hepatocytes, but the amount of binding was reduced when WDR6 was absent (Fig. 6j). Taken together, these results suggest that WDR6-mediated regulation of hepatic DNL occurs via its interactions with PPP1CB to promote dephosphorylation of PPP1CB at Thr316.

### XLIX inhibits WDR6–PPP1CB interaction and reduce hepatosteatosis

**Fig. 7:**
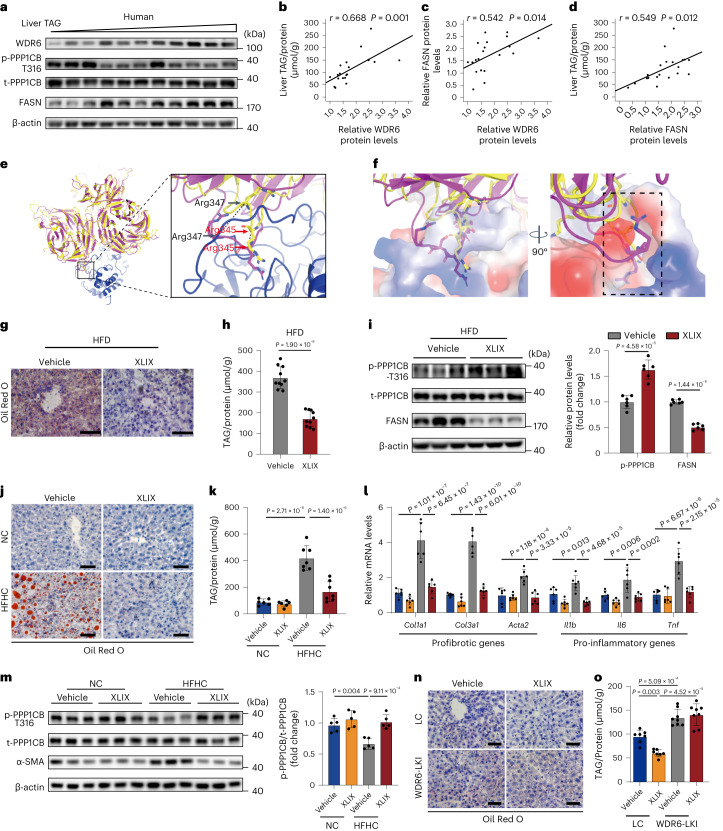
WDR6 may be a potential therapeutic target for hepatic steatosis. **a**, Representative western blots of phospho-PPP1CB (Thr316), WDR6 and FASN in human liver samples. *n* = 20 independent samples. **b**–**d**, Scatter diagram of the correlation of WDR6 and liver TAG levels (**b**), WDR6 and FASN levels (**c**), FASN and liver TAG levels (**d**) in human liver tissues. *n* = 20 independent samples. **e**, Left: MD simulation of the interaction of PPP1CB (blue) and superimposition of WDR6 with (yellow) or without (pink) coupling XLIX. Right: the Cα of key amino acids (Arg347 in black, Arg345 in red) with displacement change when binding XLIX. Black dashed lines indicate the distances between Cα atoms. **f**, Detailed interactions of WDR6 binding in PPP1CB. Black dashed box indicates the binding pocket of PPP1CB. **g**, Representative ORO staining of liver sections in XLIX and vehicle groups. Scale bars, 50 μm. **h**, Liver TAG levels of mice described in **g**, *n* = 10 biologically independent mice per group. **i**, Western blots of phospho-PPP1CB (Thr316) and FASN protein levels in livers of the mice described in **g**; *n* = 6 biologically independent mice per group. β-actin served as the loading control. **j**, Mice with HFHC or NC feeding were treated with XLIX or vehicle. Representative ORO staining of liver tissue is shown. Scale bars, 50 μm. **k**, TAG levels of the liver tissue described in **j**. *n* = 7 and 6 biologically independent mice in HFHC and NC groups, respectively. **l**, RT–PCR of representative genes of pro-inflammatory and profibrotic genes in the liver tissue described in **j**. *n* = 6 biologically independent mice per group. *Actb* served as the normalization control. **m**, Western blots of phospho-PPP1CB (Thr316) in the liver tissue described in **j**. *n* = 5 biologically independent mice per group. **n**, Representative ORO staining of liver sections in WDR6-LKI or LC given XLIX or vehicle. Scale bars, 50 μm. **o**, Liver TAG levels described in **n**. *n* = 7 biologically independent mice in LC group and 8 biologically independent mice in WDR6-LKI group. Experiments in **j**–**o** were performed using male mice. Data in **b**–**d** were analysed by linear regression and Pearson correlation coefficient (two-sided test). Data in **h** and **i** are presented as the mean ± s.d., determined by unpaired two-sided Student’s *t*-test. Data in **k**–**m** and **o** are presented as the mean ± s.d., determined by two-way ANOVA and Tukey’s multiple-comparisons test. For western blots, β-actin served as the loading control. TAG levels were normalized to the protein content of the relevant sample. Source data

To extend our findings to human samples, the protein levels of WDR6 and FASN, as well as liver TAG contents, were detected in 20 liver tissue samples collected from individuals with hepatobiliary tumours who underwent liver resection in Shandong Provincial Hospital between March 2016 and April 2021 (Supplementary Data Fig. 5a). The tissue samples (‘Human liver samples’) were confirmed as non-tumour liver tissues by pathology^45,46^ (Supplementary Data Fig. 5b–d). WDR6 and FASN levels were elevated in liver samples with higher TAG levels, whereas phospho-PPP1CB (Thr316) levels were lower (Fig. 7a). Liver WDR6 protein levels showed a positive correlation with liver TAG or FASN levels in clinical liver samples (Fig. 7b–d).

**Extended Data Fig. 9:**
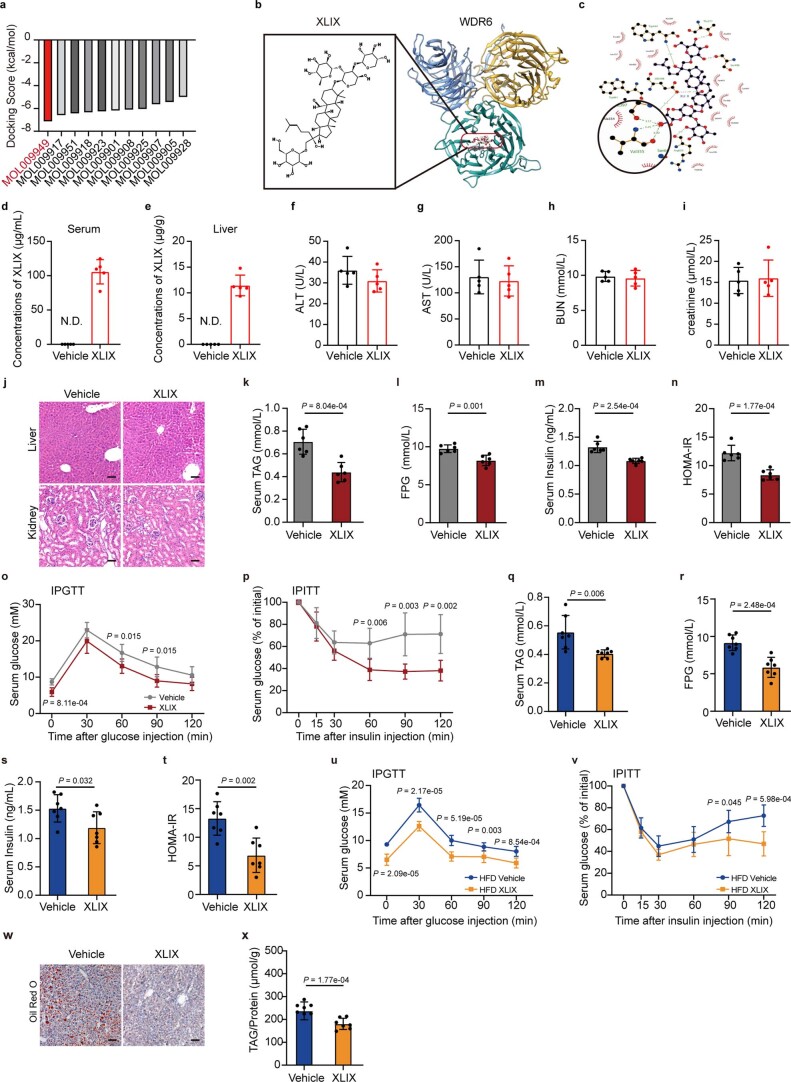
The binding details of XLIX with WDR6, and tissue distribution of XLIX, related to Fig. 7. **a**, Molecular docking of WDR6 with indicated small molecule compounds. MOL009949 stands for XLIX. **b**, Left panel, chemical structure of XLIX. Right panel, molecular dynamics simulation of the interaction of WDR6 with XLIX. **c**, The predicted binding region of XLIX withWDR6. **d**,**e**, XLIX contents in serum (**d**) and liver (**e**) of 8-week-old C57BL/6J mice given XLIX (40 mg/kg/d, i.p.) or vehicle for 1 h. The concentrations were normalized to serum volume (mL) (**d**) and tissue weights (**g**) (**e**), respectively. **f**-**i**, Serum ALT (**f**), AST (**g**), BUN (**h**) and creatinine (**i**) levels of 4-week XLIX (40 mg/kg/d, i.p.) treated mice. **j**, Representative H&E staining of liver and kidney sections of mice indicated in (**f-i**). Scale bars, 50 μm. **k-n**, Serum TAG (**k**), FPG (**l**), insulin levels (**m**) and HOMA-IR index (**n**) of male mice after 3-week HFD feeding, followed by 4-week treatment with XLIX. **o,p**, IPGTT and IPITT of mice described in (**k-n**). **q-t** (♀), Serum TAG (**q**), FPG (**r**), insulin levels (**s**) and HOMA-IR index (**t**) of female mice described in (**k-n**). **u, v** (♀), IPGTT and IPITT of female mice described in (**k-n**). **w** (♀), Representative ORO staining of liver sections of female mice described in (**k-n**). Scale bars, 50 μm. **x** (♀), Liver TAG levels were measured in female mice described in (**k-n**), the concentrations were normalized to the protein content of the same sample. For (**d**-**i**), n = 5 biologically independent mice per group. (**k**-**p**), n = 6 biologically independent mice per group. (**q**-**v**), (**x**), n = 7 biologically independent mice per group. Data in (**d**)-(**i**), (**k**)**-**(**v**) and (**x**) are presented as mean ± SD, determined by unpaired two-sided Student’s *t*-test. * *P* < 0.05, ** *P* < 0.01, *N.D*. not detected. Source data

Among domains of WDR6, the largest binding pocket was located in domain b (amino acids 330–687), which contains the putative PP1-binding motif FKSRSR. We screened the binding ability of 11 monomers from the plant *Gynostemma pentaphyllum* to the pocket of WDR6 by molecular docking and found that XLIX had the strongest binding ability with WDR6 (Extended Data Fig. 9a). The simulations implied that XLIX interacted well with WDR6, through multiple hydrogen bonds formed with several amino acid residues, such as Val355/Leu397 (Extended Data Fig. 9b,c), which are close to the FKSRSR motif and might exert steric hindrance effects on the interaction between WDR6 and PPP1CB. To further investigate the binding mode, we carried out in silico protein–protein docking to construct the WDR6–PPP1CB complex, which was then subjected to molecular dynamics (MD) simulation. The results indicated that the putative PP1-binding motif of WDR6, FKSRSR (residue 342–347), formed a loop that could insert into one pocket of PPP1CB (Fig. 7e). In detail, Arg345 within the FKSRSR loop was most deeply inserted into the pocket in PPP1CB (Fig. 7e). In the WDR6–XLIX complex, the conformation of the FKSRSR loop was notably changed after binding to XLIX. The distances of movement of the Cα atom in Arg345 and Arg347 were 3.5 Å and 8.9 Å, respectively. Therefore, we speculate that the allosteric effect caused by XLIX might disrupt the interaction of the FKSRSR loop with PPP1CB (Fig. 7f).

This interference prompted us to explore the effects of XLIX on liver lipid deposition in vivo. In the literature, Gypenosides possess many pharmacological and biological activities including lipid-regulating and hepatoprotective effects^47,48^. In vivo studies showed that XLIX could be detected in plasma with a half-life of 7–12 h and was able to alleviate kidney injury and atherosclerosis^49,50^. In our study, XLIX can be detected in serum and liver tissue 1 h after injection (Extended Data Fig. 9d,e). The drug safety was estimated in mice treated for 4 weeks with XLIX (40 mg per kg body weight per day, i.p.) by evaluating both renal and hepatic functions (Extended Data Fig. 9f–j).

Next, we tested whether XLIX could ameliorate hepatic lipid deposition. Considering NASH, rather than simple hepatic lipid deposition or IR, is the pathological state with the greatest unmet clinical need^51,52^, XLIX treatment was performed in both the HFD-induced NAFLD and the high-fat high-cholesterol (HFHC) diet-induced NASH models.

For the HFD model, because the 2-week HFD already resulted in higher liver lipid deposition (Extended Data Fig. 3b–e), we performed 3-week HFD feeding of mice, followed by 4-week treatment with XLIX (40 mg per kg body weight per day, i.p.). Compared with the control vehicle-treated group, in the XLIX-treated group, the serum lipid and insulin sensitivity were improved (Extended Data Fig. 9k–p). Moreover, liver lipid deposition and TAG levels decreased (Fig. 7g,h). Notably, phospho-PPP1CB (Thr316) increased and FASN level decreased (Fig. 7i). Similar results were observed in the HFD-induced female mouse model (Extended Data Fig. 9q–x).

**Extended Data Fig. 10:**
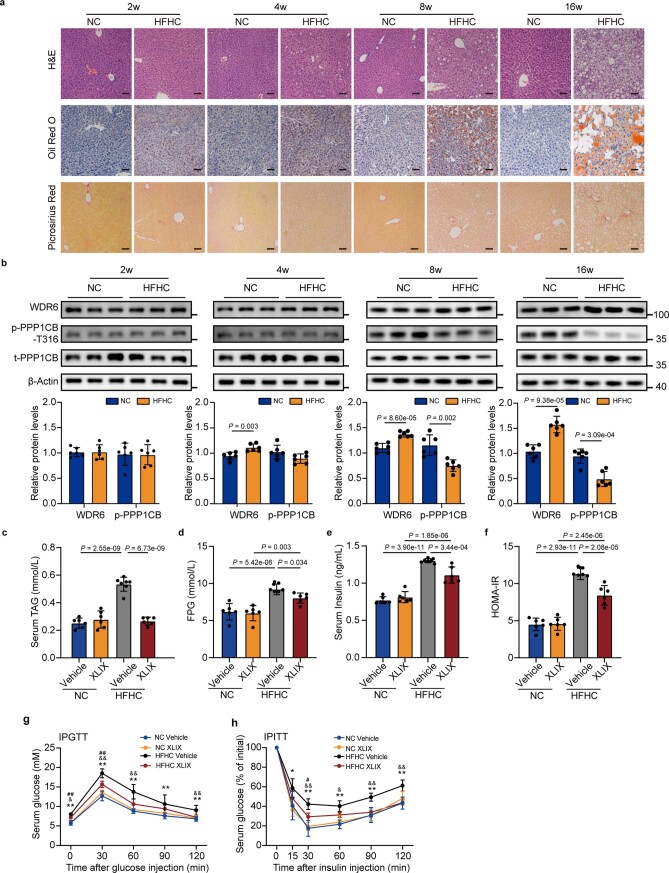
Liver Wdr6 protein levels increased with the progression of NASH, related to Fig. 7. 8-week-old C57BL/6J mice were fed high fat high cholesterol diet (HFHC group) or standard control diet (NC group) for 2, 4, 8 or16 weeks, respectively. **a**, Representative H&E, Oil Red O and Picrosirius red staining of liver sections for the indicated groups. Scale bars, 50 μm. **b**, Western blots of WDR6 protein and phospho-PPP1CB (Thr316) levels in livers of mice in the indicated groups. n = 6 biologically independent mice per group. β-Actin served as a loading control. **c-f**, Serum TAG (**c**), FPG (**d**), insulin levels (**e**) and HOMA-IR index (**f**) of mice with HFHC diet for 16 weeks, with additional XLIX- or vehicle- treatment for 4 weeks. **g, h**, IPGTT and IPITT of mice described in (**c-f**). For (**c**-**h**), n = 7 biologically independent mice in HFHC vehicle group, n = 6 biologically independent mice in other groups. Data in **(b)** are presented as mean ± SD, determined by unpaired two-sided Student’s *t*-test, data in (**c**)-(**h**) are presented as mean ± SD, determined by two-way ANOVA and Tukey’s multiple-comparisons test. * indicates statistically significance between HFHC vehicle *vs*. NC vehicle group, ^#^ indicates statistically significance between HFHC XLIX *vs*.NC XLIX group, ^$^ indicates statistically significance between NC XLIX *vs*. NC vehicle group. ^&^ indicates statistically significance between HFHC XLIX *vs*. HFHC vehicle group. *, ^#^, ^$^, ^&^, *P* < 0.05, **, ^##^, ^$$^, ^&&^, *P* < 0.01. Source data

For the NASH model, liver WDR6 was increased in mice with a HFHC diet, along with the pathological progression of NASH, in the 2- to 16-week time period of feeding, and that phospho-PPP1CB (Thr316) was, as expected, decreased (Extended Data Fig. 10a,b). Serum TAG/FPG and IR were also deteriorated. After a 4-week XLIX treatment, mice showed clearly ameliorated serum lipid disorders and improved insulin sensitivity (Extended Data Fig. 10c–h). More importantly, the lipid deposition and TAG levels (Fig. 7j,k) were also decreased. The expression of several marker genes, such as pro-inflammatory-related genes *Il1b*, *Il6* and *Tnf* and profibrotic genes *Col1a1*, *Col3a1* and *Acta2* (encoding protein α-SMA)^53,54^, were also decreased (Fig. 7l). These data imply that the progression of liver inflammation and fibrosis are alleviated by XLIX. Importantly, similarly to the XLIX-treated HFD mouse model, the level of phospho-PPP1CB (Thr316) was increased in the XLIX-treated NASH mouse group (Fig. 7m).

Lastly, XLIX treatment was performed in WDR6-LKI mice to assess whether XLIX is still effective in ameliorating lipid deposition in mice with excess levels of WDR6 (with approximately 300-fold overexpression at the transcriptional level; Supplementary Data 4b). We found that liver-specific *Wdr6* overexpression blunted the ability of XLIX to decrease liver lipid accumulation (Fig. 7n,o). Together, these results indicate that XLIX can ameliorate the progression of NAFLD and NASH, likely by enhancing the phosphorylation of PPP1CB at Thr316, in a manner that involves WDR6.

## Discussion

In this study, we discovered a previously unreported pivotal regulator of hepatic DNL, WDR6. During IR, hepatic WDR6 is upregulated, and promotes hepatic DNL by inducing the dephosphorylation of PPP1CB, which leads to increased expression of *Fasn*. Further genetic or pharmacological targeting of WDR6 can effectively alleviate hepatic TAG accumulation associated with IR.

An increase in hepatic steatosis and its relationship to IR have been noted globally^55^. Several studies have reported that such increased lipid deposition is attributed to hyperinsulinaemia, or secondary effects of ectopic lipid deposition in liver due to systemic IR. It was reported that WDR6 responds to IGF-1/insulin stimulation in mouse hypothalamus-derived GT1-7 cells^22^, and that it can interact with LKB1 (ref. ^23^), which implies that WDR6 could be involved in insulin-regulated metabolic processes. Here, we found that, in the normal state, deficiency of hepatic WDR6 did not obviously affect lipid metabolism. However, during IR, which can be considered as a pathological state, WDR6 was upregulated and served as a key molecule to induce DNL. Consistent with this, hepatic DNL did not respond much to insulin in mice with liver-specific deficiency of WDR6 during IR. These results suggest that targeting hepatic WDR6 can effectively alleviate IR-associated liver lipid deposition and the progression of NASH.

WD40 repeat-containing proteins are reported to have various functions, although, to date, the reporting on the function for WDR6 is limited, especially in vivo. It has been reported that in HEK293 cells, Cul4-DDB1-WDR3/WDR6, an E3 ubiquitin ligase complex, binds SPAK and OSR1 kinases in a phosphorylation-dependent manner^56^. In this case, WDR6 forms a complex together with an E3 ubiquitin ligase, which catalyses the ubiquitination of phosphorylated target proteins. These clues prompted us to assess whether WDR6 could induce degradation of PPP1CB by ubiquitination. However, total PPP1CB content did not show any clear change in WDR6-WKO hepatocytes (Fig. 4g,h), so the effect of WDR6 on PPP1CB may occur in a ubiquitination-independent manner.

Mammalian PP1 is an important cellular serine/threonine phosphatase family, regulating a broad range of cellular processes^57,58^. PPP1CB is one of the three distinct isoforms of the PP1 catalytic subunit. Previous studies have described many regulatory roles of PP1 (refs. ^36,59^). PP1 acts as a nutrient sensor that dephosphorylates and activates DNA-PK in response to feeding. Activated DNA-PK subsequently phosphorylates and activates USF1 (ref. ^36^). However, the modification and subsequent regulation of the catalytic subunit of PP1 have been rarely reported and, to the best of our knowledge, the phosphorylation of PPP1CB at Thr316 has not previously been described. Based on previous reports, we suspected that DNA-PK and USF1 might be involved in the process by which PPP1CB regulates FASN expression, and our further data are consistent with that hypothesis. However, as PP1 can regulate a broad range of cellular processes and the catalytic unit is highly conserved, other PP1 substrates should be tested to determine if Thr316 of PPP1CB influences the catalytic activity of PP1 against them.

Other than the highly conserved catalytic subunit, mammalian PP1 also possesses regulatory subunits, through which the substrate selectivity of PP1, and its localization, can be targeted and its activity can be regulated^34,35^. The regulatory subunits could also be defined as PPP1C-interacting proteins (PIPs). The sequence similarity between PP1 isoforms is about 90%, while PIPs are primarily proteins with unrelated structures that determine where and when a PP1 holoenzyme is active, and the types of substrates it acts on^60^. Based on the biological effects on PPP1C, the PIPs can be divided into three groups: activity-modulating proteins, targeting proteins and PPP1C substrates^61^. The first of these can modulate PPP1C activities, while the targeting proteins can perform an assembly function that facilitates the interaction of PPP1C with its substrates^62,63^. In our study, WDR6 appears to possess certain characteristics of PIPs. Firstly, WDR6 can interact with PPP1CB. Secondly, PPP1CB-Thr316 is a WDR6-related dephosphorylation site. Thirdly, we found that WDR6 harbours a putative PP1-binding motif ‘FKSRSR’, which was previously recognized as a PPP1C-dependent apoptotic signature^38^. We show here that a truncated WDR6 protein with mutations in the conserved residues of this motif no longer binds to PPP1CB. Interestingly, APAF-1, a key regulator of the mitochondrial apoptosis pathway, can bind to PP1 through its C-terminal regulatory domain, which contains 12 or 13 WD40 repeat domains^64^. Taken together, we believe that WDR6 can interact with PPP1CB, allowing it to promote DNL by upregulating *Fasn* expression.

Presently, simple hepatic steatosis is considered as a benign condition for the purposes of drug development and clinical trials; thus, it is not an unmet clinical need. However, it is well recognized that NAFLD is a major cause of chronic liver disease^65^. In 2019, 6.6% of total cirrhosis and liver cancer cases from all chronic liver diseases were attributable to NAFLD globally. Further, 8.6% of liver cancer deaths were related to NAFLD^66^. Given its increasing prevalence, NAFLD poses a substantial health burden worldwide^66,67^. Moreover, it was reported that alleviating liver steatosis could also improve insulin sensitivity^68^. These studies are in accordance with our findings of improved insulin sensitivity in mice in which WDR6 was manipulated, which demonstrates the necessity of early intervention of NAFLD. Indeed, although NASH is considered the unmet clinical need, we would argue that for any disease, early intervention is an important means to effectively control pathological progression and improve systemic insulin sensitivity. Based on MD simulation analysis, the compound XLIX was predicted to disrupt the interaction of WDR6 with PPP1CB through competitive binding and was effective in the amelioration of the phenotypes of NASH. It has been reported that XLIX can be detected by the liver^49^; our data also show that non-metabolized XLIX could be detected in the serum and liver. These results confirm that the liver is, at least, one of the major target tissues of XLIX, and that, as the effect of the compound on the liver is notably blunted in WDR6-LKI mouse, XLIX relies on WDR6 for at least some of its beneficial effects.

Our study supports the notion that there is an alternative pathway, aside from the canonical insulin signalling pathway, for insulin to drive hepatic DNL and that changes in WDR6 expression may explain the elevated hepatic steatosis. However, there are still some limitations. Firstly, further mechanistic insight is needed to explain in detail how hyperinsulinaemia increases WDR6 expression. Secondly, as PP1 can regulate a broad range of cellular processes and the catalytic unit is highly conserved, other PP1 substrates should be tested to determine if Thr316 of PPP1CB influences the activity of PP1 against them. Lastly, the detailed mechanism of XLIX should be clarified, and the effect of XLIX on the binding of WDR6 to PPP1CB also needs to be further evaluated.

## Methods

### Human liver samples

The human liver samples used in this study came from normal liver tissues (>2 cm from the distal end of the para-tumoural area, confirmed by pathological section in hepatobiliary surgery^45,46,69^). The age of participants ranged from 18 to 80 years. Participants with viral infections (for example, hepatitis B virus and hepatitis C virus), taking medicines that affected serum lipids and with excessive alcohol consumption (>140 g for men or >70 g for women, per week) were excluded. All participants provided written informed consent before sample collection. The procedures were approved by the Ethics Committee of the Shandong Provincial Hospital.

### Animals

All animal experiments were authorized by the Animal Ethics Committee of Shandong Provincial Hospital. C57BL/6J mice (male, 19–23 g, 7-week-old, or female, 16–20 g, 7-week-old, the same below unless otherwise specified) were purchased from Vital River Laboratory Animal Technology and housed under specific-pathogen-free conditions in Shandong Provincial Hospital. The mice were maintained on a 12-h light–dark cycle at 22 ± 0.5 °C, 50–60% humidity, with food and water provided ad libitum.

To establish the diet-induced IR model, 8-week-old mice were fed a HFD (60 kcal% fat, D12492; Research Diets) or CD (15 kcal% fat, 2151; Beijing KeaoXieli Feed) for at least 6 weeks^20^ or the indicated lengths of time. For the insulin-treated mouse model, mice were fasted overnight (16 h) and stimulated with insulin (0.75 units per kg body weight per day, i.p. HI0219; Lilly Egypt) or its vehicle BSA (A1933; Sigma-Aldrich) for 6 h. For the fasted–refed mouse model, mice were fed on CD/HFD for 6 weeks, then fasted overnight (16 h) and then refed for 6 h. Normally fed mice were allowed food ad libitum. To establish the HFrD model, mice were placed on either a CD or a HFrD (60% fructose; Jiangsu Xietong Pharmaceutical Bio-engineering) for 4 weeks^70^. To establish the NASH model^53^, 8-week-old mice were fed a HFHC diet (TP26304; TrophicDiet) for 2, 4, 8 and 16 weeks. The control mice were fed the standard control diet (TP26358; TrophicDiet). For XLIX treatment, 8-week-old mice were given XLIX (40 mg per kg body weight per day, i.p. A0605; Must Bio-Technology) or vehicle DMSO treatment for additional 4 weeks.

*Wdr6* (gene ID: 83669) whole-body knockout (WDR6-WKO) mice were generated by deleting exon 2 of *Wdr6* using the CRISPR–Cas9 system (Cyagen Biosciences). The sgRNA sequence was as follows: gRNA1 (matches forward strand of gene): TGGGATGTCCGCTGGATCGAGGG; gRNA2 (matches reverse strand of gene): GCCCAATGTGATTCTCCGGGAGG. For genotyping, genomic DNA was extracted from mouse tail biopsy samples, and subjected to standard genotyping PCR. PCR amplification products were as follows: WT allele = 2,294 bp, delete allele = 590 bp.

SREBP1c-KO (gene ID: 20787) mice were kindly provided by Y. Guan^71^. For genotyping, PCR amplification products were as follows: WT allele = 530 bp, delete allele = 170 bp.

*Usf1*^−/−^ (gene ID: 22278) mice were purchased from GemPharmatech (strain ID: T043964). For genotyping, PCR amplification products were as follows: WT allele = 1,751 bp, delete allele = 489 bp. Littermate WT mice were used as control.

Hepatocyte-specific *Wdr6*-knockout mice were generated by inserting two LoxP sites into the flank of exons 2–6 of *Wdr6* through the CRISPR–Cas9 system (GemPharmatech). Homozygous *Wdr6*^loxp/loxp^ mice were subsequently crossed with Albpro::Cre mice (with Albpro::Cre specifically into H11 site to obtain liver-specific Cre-expressing mice, purchased from GemPharmatech, strain ID: T003814) to obtain *Wdr6* liver-specific knockout mice (*Wdr6*^loxp/loxp^ Cre^+/−^, WDR6-LKO), together with WT littermates (*Wdr6*^loxp/loxp^ Cre^−/−^, WDR6-LNC). For genotyping, PCR amplification products were as follows: WT allele = 4,498 bp, delete allele = 414 bp.

To generate hepatocyte-specific knockdown of *Wdr6*, mice were injected with AAV2/8-TBG-shWDR6 (targeting sequence: 5′-AGGTGAAGCTCTTAGAGAA-3′) or AAV2/8-TBG-shNC (targeting sequence: 5′-TTCTCCGAACGTGTCACGT-3′) into the tail vein at a dose of 7 × 10^11^ viral genomes (vg) per mouse. This primarily targets the shRNA to the liver given the tropism of HBAAV2/8-TBG^72,73^. AAV was obtained from Hanbio Biotechnology.

To perform hepatocyte-specific knock-in of *Wdr6* in mice (WDR6-LKI), a CAG promoter-driven LoxP-stop codon-LoxP-*Wdr6* CDS region was specifically inserted to H11 gene locus to generate *Wdr6-ki*^loxp/loxp^ mice (GemPharmatech). Twelve-week-old *Wdr6-ki*^loxp/loxp^ mice were injected with AAV-TBG-Albumin-Cre (or AAV-TBG-GFP as a control) via tail veins (10^11^ vg per mouse) to delete the stop codon and start *Wdr6* expression in liver tissue. AAV was obtained from Hanbio Biotechnology.

For adenovirus-induced *Wdr6* overexpression, mice were injected with adenoviral *Wdr6* (Ad-WDR6, adenovirus expressing WDR6), or adenoviral green fluorescent protein (Ad-GFP, empty vector adenovirus) as a control, into the tail vein at a dosage of 3 × 10^8^ plaque-forming units per mouse, three times with a 5-d interval. The adenovirus was purchased from Genechem. For AAV-induced PPP1CB-Thr316Asp overexpression, 8-week-old mice were injected with AAV2/8-PPP1CB-Thr316Asp (AAV2/8-mPPP1CB Thr316Asp-3×Flag- ZsGreen, Hanbio Biotechnology) or AAV2/8-GFP (AAV2/8-TBG-mir30-3×-ZsGreen) into the tail vein at a dose of 7 × 10^11^ vg per mouse. Mice were euthanized and liver samples were analysed 12 weeks after AAV injection.

The primers used for genotyping of mice are provided in Supplementary Data 6. Primer sequences for adenoviral and adeno-associated viral plasmid construction are listed in Supplementary Data 7.

### Isolation and culture of mouse primary hepatocytes

Primary hepatocytes were isolated from male mice using the two-step collagenase perfusion protocol, as previously described^74^. The isolated hepatocytes were then incubated overnight at 37 °C (95% relative humidity, 5% CO_2_) before further experiments, including siRNA transfection, or transcriptomic, or phosphoproteomic or DNL analyses. For NASH mice, 12 h after isolation, primary hepatocytes were treated with XLIX (200 μmol l^−1^, Must Bio-Technology) or vehicle for 36 h.

### Cell cultures

HEK293 cells (GNHu 43, Cell Library of the Chinese Academy of Sciences) were cultured in DMEM-high glucose (C11995500BT, GIBCO) supplemented with 10% FBS. HepG2 cells (SCSP-510, Cell Library of the Chinese Academy of Sciences) were routinely maintained in MEM (41500-034, GIBCO) supplemented with 10% FBS. Hepa1-6 cells (TCM39, Cell Library of the Chinese Academy of Sciences) were cultured in DMEM-high glucose (C11995500BT, GIBCO) supplemented with 10% FBS. Both media were supplemented with 100 U ml^−1^ penicillin and 100 μg ml^−1^ streptomycin, and the cells were cultured at 37 °C in a humidified atmosphere with 5% CO_2_. Cells were authenticated by the Cell Library from which they were sourced and were not contaminated by mycoplasma.

To construct HepG2 cells expressing endogenous WDR6–FLAG (WDR6–FLAG cells), 800 bp of the upstream and downstream flanking sequences of *WDR6* stop codon were selected as homology arms, and subsequently inserted into the donor vector and located upstream and downstream of the 3×FLAG tag. HepG2 cells were co-transfected with donor vector and sgRNA (GGTATGACTGAGGTATCCTGCGG) by electro-transfection (1300 V, 10 ms, 2 pulse). Forty-eight hours after transfection, single-clonal cells were picked and subjected to drug screening by using 1.5 μg ml^−1^ puromycin. The primer sequences of genotyping are listed in Supplementary Data 6. The sizes of F-R2 amplicons in WT and WDR6–FLAG cells were predicted to be 925 bp and 991 bp, respectively. No amplification products were predicted by F-R1 primers in WT cells, while the amplicon in WDR6–FLAG cells were predicted to be 792 bp.

### Cell treatment

When treated with the reagents, the cells were washed twice with PBS and then starved for 2 h in serum-free medium before treatment. To assess the response of WDR6 expression to insulin, cells were treated with insulin (100 nM, I1882; Sigma-Aldrich) for 0, 2 or 12 h. To inhibit INSR activity, WDR6–FLAG cells were cultured with 10 nM S961 (INSR inhibitor, HY-P2093B; MedChemExpress) for 4 h. To knockdown *Insr*, cells were transfected with siRNA targeting *Insr* for 48 h. For phosphatase screening, HepG2 cells were treated with calyculin A (0, 0.01, 0.1 or 1 nM, SC0348; Beyotime Biotechnology) or okadaic acid (0, 5, 25 or 100 nM, MedChemExpress; Sigma-Aldrich) for 24 h.

### Intraperitoneal glucose tolerance tests

Mice fasted for 18 h were given glucose (1.5 g per kg body weight, i.p.), and blood glucose was measured using a glucometer (ACCU-CHECK, Roche) at 0, 30, 60, 90 and 120 min thereafter.

### Intraperitoneal insulin tolerance tests

Mice fasted for 4 h and were injected with insulin (0.75 U per kg body weight, i.p.; HI0219, Lilly Egypt), and blood glucose was measured at 0, 15, 30, 60, 90 and 120 min after insulin injection.

### Transmission electron microscopy analysis

Liver tissues were fixed in 3% glutaraldehyde for 2 h and in 1% osmium tetroxide for 1 h. The fixed tissues were dehydrated through ethanol concentration gradient washing and embedded in epoxy resin. Ultrathin sections were obtained and observed by transmission electron microscope (H-800, Hitachi).

### Liver B-mode ultrasound imaging

The mice were evaluated using ultrasound (Vevo 2100, 30-MHz; VisualSonics). The mice were anaesthetized with sodium pentobarbital (30 mg per kg body weight, i.p.). The fur on the abdomen was removed using a depilatory cream. During the entire imaging process, the mice were positioned on a warmed platform to track the body temperature, heart rate and respiration. Hepatic/renal and maximum liver planes were analysed. Average intensity/mm^2^ (a.u.) was calculated by taking the average intensity of three different areas of each plane^75^. All ultrasound images were captured using the same settings (frequency = 30 MHz, gain = 28 dB, depth = 12 mm, dynamic range = 60 dB, width = 15.36 mm, persistence = off, sensitivity = high) from the same machine and completed by the same technician, who was blinded to this study.

### Magnetic resonance examination

MR was performed on a 9.4 Tesla (9.4 T) BioSpec magnetic resonance scanner (Bruker). The mice were anaesthetized with 4.0% isoflurane gas and maintained for 3–4 h using 1.5% gas with oxygen flowing at 1.2 l min^−1^. A volume coil with an inner diameter of 40 mm was used for RF transmission and signal reception. Multi-slice spin-echo trans-axial images were obtained using a repetition time of 1,000 ms, echo time of 8 ms and a data matrix size of 128 × 128. Fat and water images with a slice thickness of 1.5 mm were collected with a field of view of 30 × 30 mm^2^. The single voxel point-resolved selective spectroscopy (PRESS) technique was used to determine the 1^H^-MRS fat content in the liver. Data were acquired with a repetition time of 1,500 ms, an echo time of 16 ms and bandwidth at 8,196 Hz. Motion artefacts were minimized by applying cardiac and respiratory gating to all MRI studies. All animals were scanned using the settings and parameters described above. This approach for MR examination was modified from a previous publication^76^.

### Positron emission tomography–computed tomography

Before PET imaging, the mice were fasted for 12 h and then anaesthetized with isoflurane (1.5–2.5%, 0.3–0.8 l min^−1^). Approximately 200–500 µCi [^11^C]acetate was injected via the tail vein, and the PET scanning was initiated immediately. Dynamic PET images were acquired for 20 min using micro-PET equipment (Pingseng Healthcare). Image reconstruction was achieved by an ordered subset expectation maximization (OS-EM 3D-PSF) algorithm based on 150 × 150 × 212 image matrices, yielding voxel spacing (*x*, *y*, *z*) of 0.6667, 0.6667, 0.6 mm, respectively. Anatomical regions of interest, specifically the liver, were identified using PMOD software (version 3.805). The uptake of radioactive material in each region of interest was determined by calculating the SUV using the following formula. The SUV reflects FASN expression and the levels of de novo FA synthesis^26^.

1 $${\rm{SUV}}=\frac{{\rm{Uptake}}\; {\rm{value}}\; {\rm{of}}\; {\rm{radioactive}}\; {\rm{material}}\; {\rm{in}}\; {\rm{regions}}\; {\rm{of}}\; {\rm{interest}}\left(\frac{{{\mu }}{\rm{Ci}}}{{\rm{g}}}\right)}{\frac{{\rm{Total}}\; {\rm{injection}}\;{\rm{ dose}}\left(\frac{{{\mu }}{\rm{Ci}}}{{\rm{g}}}\right)}{{\rm{Body}}\; {\rm{weight}}\left({\rm{g}}\right)}}\,$$

### Transcriptomic analysis

Transcriptomic analysis was carried out at Novogene. The experiment was performed as previously described^77^. In brief, sequencing libraries were generated using the NEBNext Ultra RNA Library Prep Kit for Illumina. Sequencing was then performed on an Illumina HiSeq platform. Reads of each sample were aligned to the mouse genome (NCBI build 38/mm10) using Bowtie v2.2.3. The DEGs were analysed using DESeq2 (1.32.0). Genes with |log_2_ fold change| ≥ 0.35 and *P* values < 0.05 were scored as DEGs. Volcano plots and heat maps were produced using ggplot2 (3.3.5) and pheatmap (1.0.12).

### Identification of candidate genes

Two microarray datasets (GSE66676 (ref. ^78^) and GSE48452 (ref. ^79^)) were obtained from the Gene Expression Omnibus database. Genes that showed a positive association with *FASN* level (Hmisc package (version 3.16.0), *P* values < 0.05) and that intersected in all three databases were scored as candidates (gene set 1). In our transcriptomic data, genes that responded to insulin were selected based on the upregulated (log_2_ fold change > 0.7, log_10_*P* value > 1.5) and downregulated (log_2_ fold change < −0.7, log_10_*P* values > 1.5) DEGs by pairwise comparison (IR model + insulin versus IR model + BSA; gene set 2). Genes at the intersection of gene sets 1 and 2 were identified as candidate genes.

### Lipidomic analysis

Lipidomic analysis was done in collaboration with LipidALL Technologies. The detailed lipidomic profiling was carried out according to the method described previously^80–82^.

### De novo lipogenesis

Primary hepatocytes were starved for 2 h by incubation in PBS. The cells were then incubated for an additional 24 h in DMEM-without glucose (11966025; GIBCO) supplemented with 100 U ml^−1^ penicillin, 100 μg ml^−1^ streptomycin and 25 mM d-[U-^13^C] glucose (389374-2G; Sigma-Aldrich). The fraction of newly synthesized palmitate was measured using liquid chromatography–mass spectrometry (LC–MS) as previously described^83,84^.

### Determination of palmitic acid and stearic acid content

Measurements of palmitic acid and stearic acid in mice liver were performed by using freshly isolated liver samples (10 mg ± 0.2 mg), through liquid chromatography–high-resolution mass spectrometry (LC–HRMS), as previously described^83^.

### Phosphoproteomic analysis

For preparation of samples for phosphoproteomics, primary hepatocytes were isolated from WT or WDR6-WKO mice to obtain about 1 mg of protein lysate. The phosphoproteomics experiment was performed by Jingjie PTM BioLab, with standard protocols as previously described^85^.

### Tissue distribution of XLIX

To detect the tissue distribution of XLIX, 8-week-old mice were given XLIX (40 mg per kg body weight per day, i.p., Must Bio-Technology) or vehicle DMSO treatment. One hour after injection, serum and liver biopsy samples were taken and assayed for XLIX. Sample preparation was modified according to the reported method^86^. Briefly, 200 µl and 300 µl of cold methanol including gynostemma saponin A at 1.25 µg ml^−1^ was added into the 50 µl and 10 mg of serum and liver samples, respectively. Then, samples were vortexed for 1 min followed by centrifugation at 18,624 r.p.m. for 10 min at 4 °C. The supernatant was used for LC–MS analysis.

Ultra-high-performance liquid chromatography and mass spectrometry (UHPLC–MS) analysis for Ginsenoside XLIX was modified according to a previously reported method^86^. In brief, an AB SCIEX 5600 Plus Q-TOF mass spectrometer (Applied Biosystems Sciex) equipped with an electrospray ionization (ESI) source interfaced with UHPLC (Shimadzu) was used. Chromatographic separation was performed using a BEH C18 column. The mobile phases A and B were ultrapure water and HPLC-grade acetonitrile with ammonium acetate. The volume of the sample solution injected into the chromatographic system was 5 µl. The flow rate was set at 0.35 ml min^−1^ and the total run time was 7.0 min. The negative ESI mode was used at *m/z* 150–1,250 Da. The ESI-MS/MS experiment was operated in information-dependent acquisition mode to obtain fragmentation in the negative mode. The capillary temperature was 500 °C and the capillary voltage was −4,500 V.

### FASN activity assay

FASN enzyme activity was assessed on tissue extracts of mouse liver specimens using the Fatty Acid Synthase Activity Assay Kit (BC0555; Solarbio), following the manufacturer’s instructions. The principle of this experiment was as follows: FASN utilizes acetyl-CoA, malonyl-CoA and NADPH to generate long-chain FAs and NADP^+^. NADPH had the maximum absorption peak at 340 nm. FASN activity was calculated by measuring the decline rate of light absorption at 340 nm. Protein concentrations of tissue lysates were determined using the BCA Protein Assay Kit (23225; Thermo Scientific) and enzyme activity levels were normalized to protein amount.

### Immunoprecipitation and mass spectrometry analysis

IP was performed using the Flag Immunoprecipitation Kit (FLAGIPT1-1KT; Sigma-Aldrich^87,88^) according to the manufacturer’s instructions. Briefly, the beads are designed to have affinity for the FLAG tag and can capture FLAG-tagged proteins independently of a primary antibody. Cell lysates of WDR6–FLAG HepG2 cells (HepG2 cells with endogenous WDR6–FLAG fusion protein) were incubated overnight at 4 °C with these beads. The control experiment was performed by incubating the same beads with the whole-cell lysates of WT HepG2 cells (cells that do not express FLAG). Beads containing immune complexes were washed and proteins were eluted with 150 µg ml^−1^ 3×FLAG-peptide. The input and precipitated protein samples were subjected to western blot analysis with corresponding antibodies as stated. Western blots of the elution of the control group were used to control for any non-specific binding of the beads. The specific bands from SDS–PAGE were cut and digested, followed by further MS analysis supported by Jingjie PTM BioLab.

### Preparation of phospho-PPP1CB (Thr316) polyclonal antibody

The polyclonal phosphorylation antibodies against phosphorylation of PPP1CB-Thr316 were generated by Shanghai Immune Biotech. The antigen sequence used for immunization was an appropriately phosphorylated polypeptide corresponding to amino acids 311 to 323 of PPP1CB (C-SGRPV-pT-PPRTANP). Rabbits were immunized with BSA-conjugated peptide. Sera were collected after the third immunization, and then we performed affinity chromatography purification by phosphorylated peptide C-SGRPV-pT-PPRTANP, followed by elimination of antibodies against unphosphorylated antigen C-SGRPVTPPRTANP.

### Molecular docking

The protein structure of WDR6 was predicted by the AlphaFold Protein Structure Database and prepared using Schrodinger Suite. The protonation state of the WDR6 protein was determined using PDB2PQR webserver^89^. Pocket analysis was performed using AlphaSpace^90^. Molecular docking was performed using Glide^91^ against the second domain of WDR6 (residues 330–687), and the top-scored result was used for each ligand.

### Molecular dynamics simulation

The initial WDR6–XLIX complex was obtained from the top-scored molecular docking result. MD simulation was carried out using the Amber16 package. We used the Amber ff4SB force field^92^ for the protein and the GAFF force field for the ligand. The partial charge of XLIX was assigned using AM1-BCC methods via antechamber. The system was neutralized and solvated in a rectangular periodic box with explicit TIP3P water using AmberTools17. The Particle-mesh Ewald method for nonbonded interactions was used for MD simulation. Briefly, a series of minimizations and equilibrations was performed as described previously^93^. Then, standard MD simulations were performed on the GPU using the CUDA version of PMEMD for 100 ns with periodic boundary conditions. A time step of 2 fs was used, and the system temperature was controlled at 300 K using the Berendsen thermostat method. All other parameters were at default settings. All figures and movies were created using UCSF Chimera^94^. Protein–ligand interactions were analysed using LigPlot^95^.

### Statistics and reproducibility

Statistical analyses and graphical presentations were performed using SPSS v25.0 and GraphPad Prism (version 9.5.0). Continuous variables are presented as means ± s.d. Normality tests were performed before analyses. Comparisons between two groups were performed by unpaired two-sided Student’s *t*-test or Mann–Whitney test. Comparisons among multiple groups were one-way or two-way ANOVA with Tukey’s post hoc test for continuous variables. Sample size and detailed statistical information for each experiment can be found in the corresponding figure legends. In vitro and in vivo experiments were conducted randomly. Data from animal and cell studies were collected in a blinded fashion. No data were excluded when performing the statistical analysis. All calculated *P* values are two sided, and *P* < 0.05 was considered statistically significant. The exact *P* value is indicated on the graph. The sample sizes were not pre-determined by any statistical methods but are similar to those reported in previous publications. All experiments requiring statistical analysis were performed three times with similar results.

All experiments for which statistics was derived were performed two or three times with similar results. All the replicates performed were biological and not technical. Detailed information for each experiment is provided below. Experiments shown in Figs. 2b,c,e,g, 3h, 5d,g, 6c,e and 7j and Extended Data Figs. 1c, 2s, 3d, 4r, 5h, 9j,w and 10a and Supplementary Fig. 5d were repeated three times. Experiments shown in Figs. 4b,d,e,f and 6f,j were repeated two times.

Figures 1a, 3e and 4a and Extended Data Fig. 3a were produced using Servier Medical Art by Servier (https://smart.servier.com/) under a Creative Commons Attribution 3.0 Unported Licence. Illustrations in Supplementary Data 5a,d were generated by us. The pictures of instruments in Figs. 3e and 4a were provided by MITRO Biotech and Jingjie PTM BioLab, respectively.

Further materials and methods are described in the Supplementary Information.

### Reporting summary

Further information on research design is available in the Nature Portfolio Reporting Summary linked to this article.

### Supplementary information

Supplementary Information Supplementary Methods, Supplementary Data Figs. 1–5 and Supplementary Data 6–10 (tables including primer sequences for genotyping, plasmid construction, RT–PCR and antibodies).

Reporting Summary

Supplementary Data 1 Statistical source data.

Supplementary Data 2 Unprocessed western blots.

### Source data

Source Data Fig. 1 Statistical source data.

Source Data Fig. 1 Unprocessed western blots.

Source Data Fig. 2 Statistical source data.

Source Data Fig. 3 Statistical source data.

Source Data Fig. 3 Unprocessed western blots.

Source Data Fig. 4 Statistical source data.

Source Data Fig. 4 Unprocessed western blots.

Source Data Fig. 5 Statistical source data.

Source Data Fig. 5 Unprocessed western blots.

Source Data Fig. 6 Statistical source data.

Source Data Fig. 6 Unprocessed western blots.

Source Data Fig. 7 Statistical source data.

Source Data Fig. 7 Unprocessed western blots.

Source Data Extended Data Fig. 1 Statistical source data.

Source Data Extended Data Fig. 1 Unprocessed western blots.

Source Data Extended Data Fig. 2 Statistical source data.

Source Data Extended Data Fig. 2 Unprocessed western blots.

Source Data Extended Data Fig. 3 Statistical source data.

Source Data Extended Data Fig. 4 Statistical source data.

Source Data Extended Data Fig. 4 Unprocessed western blots.

Source Data Extended Data Fig. 5 Statistical source data.

Source Data Extended Data Fig. 5 Unprocessed western blots.

Source Data Extended Data Fig. 6 Statistical source data.

Source Data Extended Data Fig. 6 Unprocessed western blots.

Source Data Extended Data Fig. 7 Statistical source data.

Source Data Extended Data Fig. 7 Unprocessed western blots.

Source Data Extended Data Fig. 9 Statistical source data.

Source Data Extended Data Fig. 10 Statistical source data.

Source Data Extended Data Fig. 10 Unprocessed western blots.

## Data Availability

All RNA-sequencing datasets have been deposited in the Gene Expression Omnibus and are publicly available (Fig. 1c and Supplementary Data Fig. 1 (GSE205460) and Extended Data Fig. 8 (GSE205459)). Proteomics and phosphoproteomics data have been deposited at the ProteomeXchange Consortium via the PRIDE partner repository and are publicly available under accession numbers PXD034367 and PXD034542. Source data are provided with this paper.
