# Supplementary material for: Upregulation of WDR6 drives hepatic de novo lipogenesis in insulin resistance in mice
Source: Nat Metab. 2023 Sep 21;5(10):1706–25. doi: 10.1038/s42255-023-00896-7 (PMC10590755; doi:10.1038/s42255-023-00896-7)
Supplement: Supplementary file 1 — Supplementary Methods, Supplementary Data Figs. 1–5 and Supplementary Data 6–10 (tables including primer sequences for genotyping, plasmid construction, RT–PCR and antibodies). [file 42255_2023_896_MOESM1_ESM.pdf]

nature metabolism

Article https://doi.org/10.1038/s42255-023-00896-7

Upregulation of WDR6 drives hepatic de novo lipogenesis in insulin resistance in mice

In the format provided by the authors and unedited

Supplementary Materials and Methods

Serum parameter measurements

FPG and serum levels of lipid profiles, ALT, AST, BUN, and creatinine were determined using enzymatic methods with an Auto Biochemical Analyzer (AU5400, Olympus). Serum insulin levels were measured using Mouse insulin ELISA kit (csb-e05071m; CUSABIO). All steps were performed in strict accordance with the manufacturer's instructions. HOMA-IR = FPG (mmol/L) × fasting insulin levels (mIU/L)/22.5.

TAG contents in liver tissue and primary hepatocytes

TAG in liver tissue and primary hepatocytes was extracted and assayed in strict accordance with the Triglyceride content assay kit (E1013; Applygen Technologies) manufacturer's instructions. The contents of TAG were normalized to those of the protein content or tissue weight in the same sample.

Oil Red O staining

Liver tissues were fixed in the OCT-Freeze medium and then cut into 10 μm thick sections. For oil red O staining, frozen sections or primary hepatocytes were fixed with 4% paraformaldehyde for 30 min before staining with Oil Red O solution (G1015; Servicebio) according to the manufacturer's instructions. Picture acquisition was done using LAS V4.9.

Hematoxylin & eosin (H&E) and Picrosirius red staining

Liver tissues were fixed in 4% paraformaldehyde, dehydrated, embedded in paraffin, cut into 5 μm thick sections. Then, the paraffin sections were stained with hematoxylin (G1004; Servicebio), eosin solution (G1002; Servicebio) or picrosirius red (365548, Sigma-Aldrich) according to the manufacturer's instructions. Picture acquisition was done using LAS V4.9.

Reactome pathway and Gene Ontology (GO) enrichment analysis

Reactome pathway analysis was performed using ReactomePA (1.36.0) and GO analysis was run using clusterProfiler (4.0.5). Pathways with a P value less than 0.05 were defined as enriched with significance.

siRNA transfection

siRNAs targeting *INSR*, *PPP1CA*, *PPP1CB* and *PPP1CC* were designed according to the common sequences in both humans and mice. The targeting sequence for *INSR* was 5'-GAACAATGTTGTACACTTATT-3'. The targeting sequence for *PPP1CA* was 5'-GAGAAGATACAACATCAAA-3'. The targeting sequence for *PPP1CB* was 5'-GCCTATAGCTGCTATTGTT-3'. The targeting sequence for *PPP1CC* was 5'-GCAAGAATGTCCAGCTCCA-3'. Lipofectamine 3000 reagent (L3000015; Invitrogen) was used for transfection according to the manufacturer's instructions. After 48 h, the cells were collected for further analysis.

Plasmid construction

Plasmids encoding full-length, mutant or truncated human PPP1CB-FLAG, HA-PPP1CB, WDR6-FLAG or HA-WDR6 were obtained by cloning the corresponding cDNA into the pcDNA3.1-Flag or pcDNA3.1-HA backbones, respectively. Primer sequences for overexpressing plasmid construction are listed in Supplementary Data 8.

RNA isolation and real-time quantitative RT-PCR

The total RNA extraction procedure has been described previously[1]. RNA concentration was determined by Nanodrop2000. The real-time PCR was performed by SYBR Green (11201ES08; Yeasen Biotechnology) on LightCycler480 Software (Roche) according to the manufacturer's instructions. *Actb* or *Acta1* were utilized as internal controls for normalization. Each sample was analyzed in triplicates, and all data were analyzed relative to the controls. Primer sequences for RT-PCR are listed in Supplementary Data 9.

Protein extraction and Western blots

Cells and tissues were solubilized in RIPA buffer (MA0151; Meilunbio) containing phosphatase inhibitor cocktail (B15001; Bimake) and protease inhibitor cocktail (R0092-010; ShenergyBiocolor Bioscience) according to the manufacturer's instructions. The protein concentration was determined using BCA Protein Assay Kit (23225; Thermo Scientific). Equal amounts of protein from different

samples were subjected to 6%-12% SDS-PAGE, followed by electro-transfer to polyvinylidene difluoride (PVDF) membranes (IPVH00010; Merck Millipore). β-ACTIN or GAPDH was utilized as an internal control for normalization. Immune complexes were detected using Amersham Imager 680. Protein quantitative analysis was applied by measuring bands densitometry using AlphaView 3.2.2. The phosphorylated protein levels were normalized to the respective 'total' protein levels, while the others were normalized to the internal control. Antibodies for western blot are listed in Supplementary Data 10.

Reference

1. Wang X, et al. Cyclophilin D deficiency attenuates mitochondrial perturbation and ameliorates hepatic steatosis. Hepatology 68, 62-77 (2018).

Supplementary Data 1

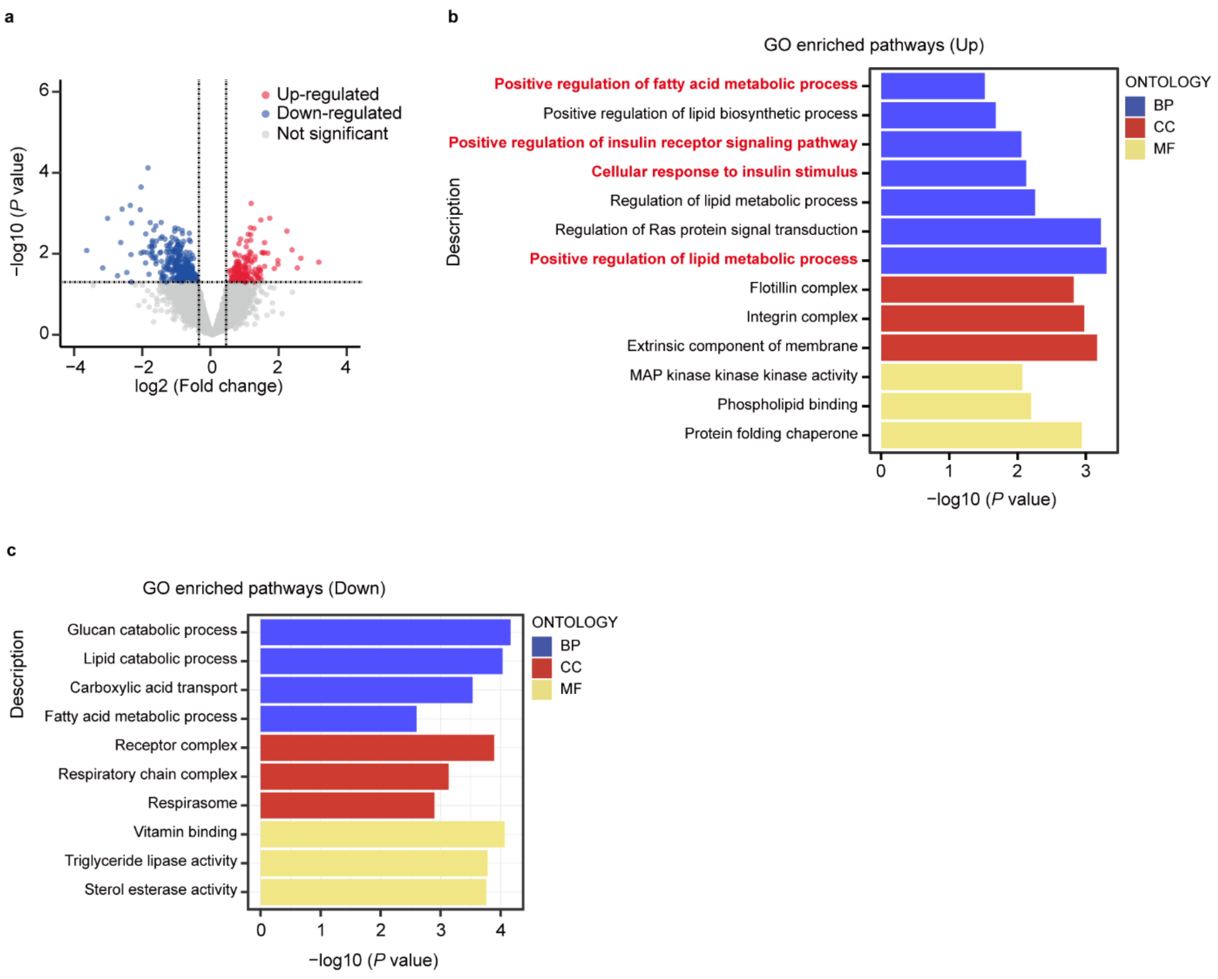

Supplementary Data 1 | Transcriptomic analysis of genes in liversdifferentially expressed in response to insulin stimulation during IR, related to Fig. 1.

a, A volcano plot showing differentially expressed genes (red, upregulated genes; blue, downregulated genes; gray, not significantly changed) in insulin group *vs*. vehicle group. **b**, GO enrichment analysis of up-regulated genes in insulin group *vs*. vehicle group. **c**, GO enrichment analysis of down-regulated genes in insulin group *vs*. vehicle group. For (**a**-**c**), n = 3 biologically independent mice per group.

Supplementary Data 2

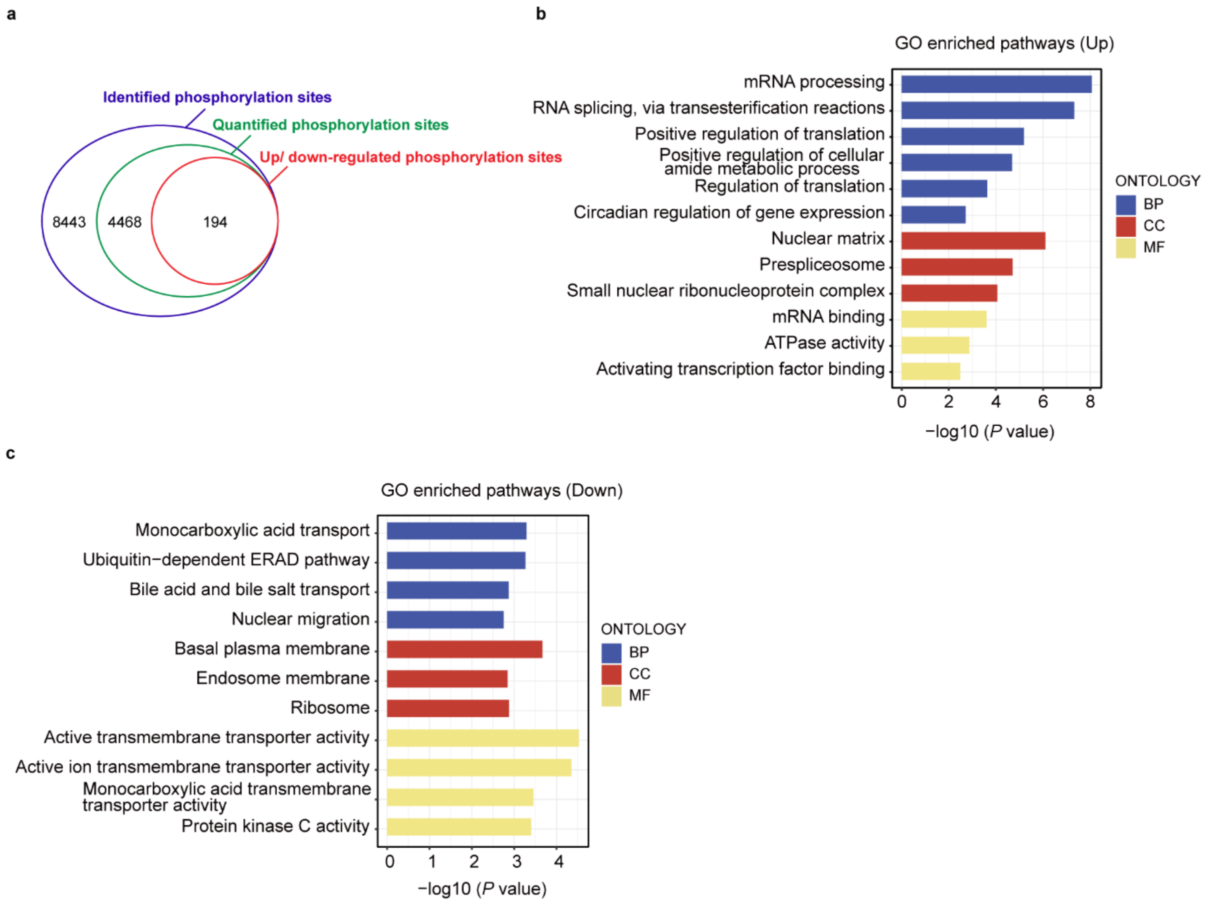

Supplementary Data 2 | Phosphoproteomic analysis of primary hepatocytes from WT and WDR6-WKO mice, related to Fig. 4.

a. The number of identified (blue), quantified (green) and quantitatively different (red) phosphorylation sites. **b.** GO enrichment analysis of proteins with increased levels of phosphorylation in WDR6-WKO *vs*. WT groups. **c.** GO enrichment analysis of proteins with lower levels of phosphorylation in WDR6-WKO *vs*. WT groups.

Supplementary Data 3

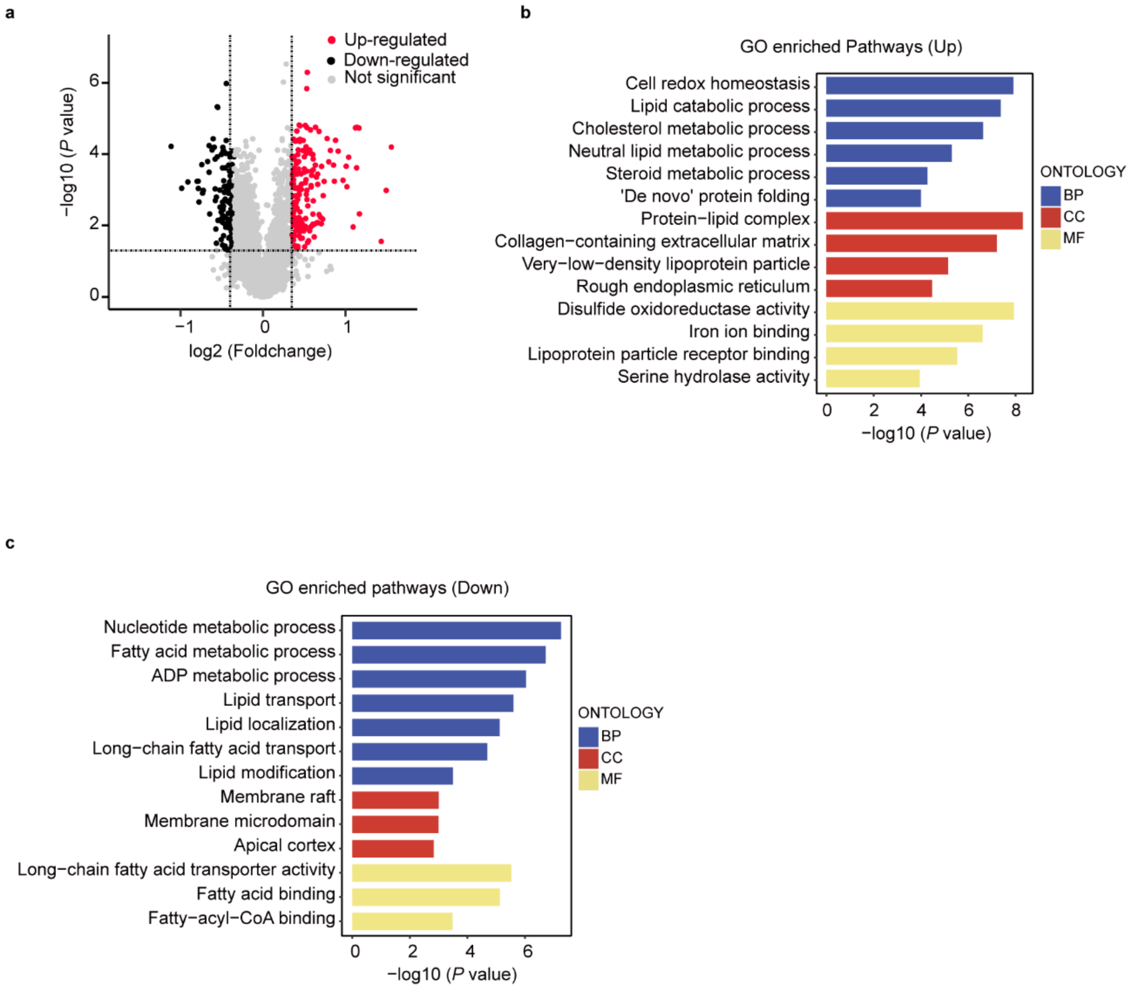

Supplementary Data 3 | Proteomic analysis of differentially expressed proteins in primary hepatocytes from WT and WDR6-WKO mice, related to Fig. 4.

a. A volcano plot showing differentially expressed proteins. Red, increased overall levels; black, lower levels, gray, no significant difference. **b.** GO enrichment analysis of proteins increased in WDR6-WKO *vs*. WT groups. **c.** GO enrichment analysis of proteins decreased in WDR6-WKO *vs*. WT groups.

Supplementary Data 4

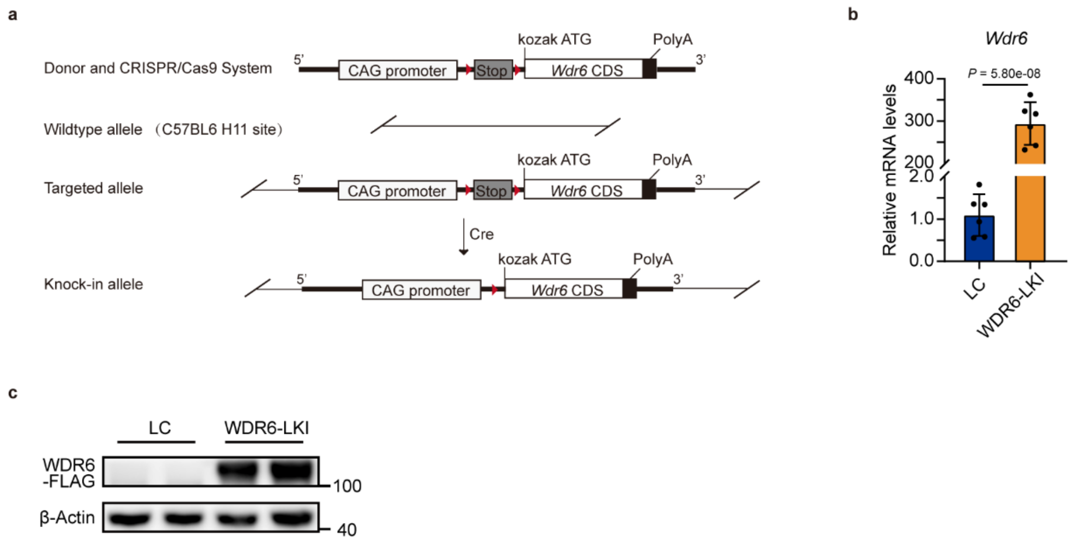

Supplementary Data 4 | WDR6-LKI mice were created and show liver specific overexpression of Wdr6, related to Fig. 4.

a. A schematic diagram illustrating the strategy for creatingWDR6-LKI mice. **b.** RT-PCR analysis of *Wdr6* mRNA levels in livers of LC and WDR6-LKI mice. Expression of *Wdr6* was normalized to *Actb* mRNA levels. n = 6 biologically independent mice per group. **c.** Western blots of WDR6-FLAG protein levels in livers of LC and WDR6-LKI mice. n = 4 biologically independent mice per group. β-Actin serves as a loading control. Data in (b) are presented as mean ± SD and analyzed by unpaired two-sided Student's *t*-test.

Supplementary Data 5

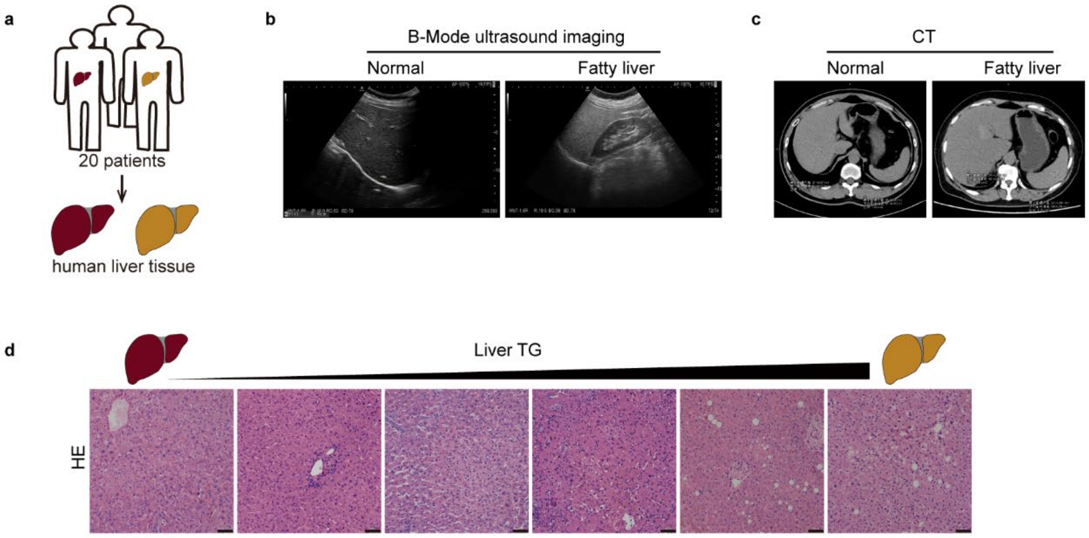

Supplementary Data 5 | The correlation of WDR6, FASN and TAG levels in human liver tissue, related to Fig. 7.

a. Schematic diagram illustrating the collection of human liver samples with different degrees of hepatic lipid deposition. **b.** Representative liver B-Mode ultrasound imaging of the above patients. **c.** Representative liver computed tomography (CT) imaging of the above patients. **d.** Representative H&E staining of liver sections of the above patients. Scale bars, 50 μm.

Supplementary Data 6 | Primer sequences for genotyping the genetically modified mice and cells.

mice/ cells	Forward primer (5'-3')
Wdr6 whole-body knockout mice	F: CTGTCTAGGCGAGGGGCCTGAT<br>R: GTCGAGTATCCTGTGGGCCACCT
Srebp1c whole-body knockout mice	F: ATCGGCGCGGAAGCTGGGGTAGCGTCT<br>R1: TCTCCAGATTTATGCAGGTCATAAATAGTAC<br>R2: GCTCAGTTCGAGGTGCTGTTTCTG
Usf1 whole-body knockout mice	F: CGTTACAGATAGTTGTGAGCCACC<br>R: CTCTATCCCTTAGCAGAACACCATG
Hepatocyte-specific Wdr6 knockout mice	F: TCTGTTGAGCCTCAGGGATTAGACTG<br>R: ACCTCAACTGCAAAGGCACACT
Wdr6 knockin flox/flox mice	F1: ATGCCCACCAAAGTCATCAGTGTAG<br>R1: AGGCGGGCCATTTACCGTAAGTTA<br>F2: AGTCTTTCCCTTGCCTCTGCT<br>R2: GGGTCTTCCACCTTTCTTCAG
WDR6-Flag HepG2 cells	F: ATCTCTGTTATTGACCAGGCTGTC<br>R1: CTTGTCATCGTCATCCTTGTAATC<br>R2: CTGTCTTGTCATGGGGAGTCG

Supplementary Data 7 | Primer sequences for adenoviral and adeno-associated viral plasmid construction.

Gene	Vector	Forward primer (5'-3')	Reverse primer (5'-3')
Ad-mWDR6	CMV-MCS-3*FLAG-SV40-EGFP	AGGTCGACTCTAGAGGATCCCGCCACCATGGACGCTTTCGGGGACTATGTCTGG	TCCTTGTAGTCCATACCGGTGTCATACCAGTTGTAAACCTCAAGTCC
AAV-mPPP1CB (316D)	TBG-3*FLAG-P2A-ZSGREEN	GCCACCATGGCGGACGGGGAGCT	CCTTTTCTTCGGTGGATTAGCTGTTCGAGGCGGATCGACAGGACGT

Supplementary Data 8 | Primer sequences for constructing overexpression plasmids.

Gene	Forward primer (5'-3')	Reverse primer (5'-3')
hWDR6-FLAG	ATGGGCAGCGCGGCGCGCTGG	TTGAGGTTTACAACTGGTATGAC
hWDR6-HA	ACGGGCCCTCTAGACTCGAGCGCCACCATGTACCCTTATGATGTCCCAGACTATGCTGG	TTAAACTTAAGCTTGGTACCTCAGTCATACCAGTTGTAAACCTCAAGC
hWDR6 (1-329aa)	GAAAACCTGTATTTTCAGGGCATGGACGCTCTCGAGGACTACG	TCGAGGCTGATCAGCGGGTTTCATTACCCTACCAAGTGCCACAGCCG
hWDR6 (334-687aa)	GAAAACCTGTATTTTCAGGGCGGATTGGGGGTCTCGGCTCTC	TCGAGGCTGATCAGCGGGTTTCATTAGCCACCCAGAGCCCTGTACAGC
hWDR6 (698-1121aa)	GAAAACCTGTATTTTCAGGGCGGTCTGCATGGCCGTGAGATCAC	TCGAGGCTGATCAGCGGGTTTCATTAGTCATACCAGTTGTAAACCTC
hWDR6 (1-699aa)	GAAAACCTGTATTTTCAGGGCATGGACGCTCTCGAGGACTACG	TCGAGGCTGATCAGCGGGTTTCATTACAGACCCTCCCGGAGAATCACG
hWDR6 (1-311, 687-1121aa)	GAAAACCTGTATTTTCAGGGCATGGACGCTCTCGAGGACTACG	TCGAGGCTGATCAGCGGGTTTCATTAGTCATACCAGTTGTAAACCTC
hWDR6-mut (AKSASA)	TCTCGGCTCTCTGCGCTGCATCCGCCAGTGCGCCAGGTACACTCAAGGCTGTG	TCGAGGCTGATCAGCGGGTTTCATTAGCCACCCAGAGCCCTGTACAGC
hPPP1CB-FLAG	GACCCAAGCTGGCTAGTTGAATTCGCCACC	TCACTTAAGCTTGGTACCGAGGATCC
hPPP1CB-HA	TACCCCTATGACGTGCCAGAC	TGCGTAGTCTGGAACGTCGTA
hPPP1CB (316A)-FLAG	GAATTCGCCACCATGGCGGACGGGGAGCT	GCGGATTAGCTGTTCGAGGTGGAGCGACAGGACGTCCAGAATTCAG
hPPP1CB (316D)-FLAG	GAATTCGCCACCATGGCGGACGGGGAGCT	CGGATTAGCTGTTCGAGGTGGATCGACAGGACGTCCAGAATTCAG

Supplementary Data 9 | Primer sequences for RT-PCR.

Gene	Species	Forward primer (5'-3')	Reverse primer (5'-3')
Acadm	mouse	AGGGTTTAGTTTTGAGTTGACGG	CCCCGCTTTTGTCATATTCCG
Acc1	mouse	GCTTATTGATCAGTTATGTGGCC	CTGCAGGTTCTCAATGCAAA
Acox1	mouse	TAACTTCCTCACTCGAAGCCA	AGTTCCATGACCCATCTCTGTC
Acox2	mouse	CCAGCACTTTGAGGAGGAGA	GGACTTGGCTTCCTTTAGGG
ApoB	mouse	AAGCACCTCCGAAAGTACGTG	CTCCAGCTCTACCTTACAGTTGA
Cd36	mouse	AAGCAAAGTTGCCATAATTGAGTC	GGAAAGGAGGCTGCGTCTG
ChREBP	mouse	AGATGGAGAACCGACGTATCA	ACTGAGCGTGCTGACAAGTC
Col1a1	mouse	TGCTAACGTGGTTCGTGACCGT	ACATCTTGAGGTCGCGGCATGT
Col3a1	mouse	ACGTAAGCACTGGTGGACAG	CCGGCTGGAAAGAAGTCTGA
Cpt1α	mouse	TTGGGCCGGTTGCTGAT	GTCTCAGGGCTAGAGAACTTGGAA
Cpt2	mouse	CAGCACAGCATCGTACCCA	TCCCAATGCCGTTCTCAAAAT
Ctgf	mouse	TGACCCCTGCGACCCACA	TACACCGACCCACCGAAGACACAG
Fabp1	mouse	ATGAACTTCTCCGGCAAGTACC	CTGACACCCCCTTGATGTCC
Fasn	mouse	GTCCTGGGAGGAATGTAAACAG	CGGATCACCTTCTTGAGAGC
G6pc	mouse	ACTAAAGCCTCTGAAACCC	AGATTCTGCACCGCAAG
Glut2	mouse	TCAGAAGACAAGATCACCGGA	GCTGGTGTGACTGTAAGTGGG
hadha	mouse	TGCATTTGCCGCAGCTTTAC	GTTGGCCCAGATTTCGTTCA
Il-1β	mouse	CCGTGGACCTTCCAGGATGA	GGGAACGTCACACACCAGCA
Il-6	mouse	TAGTCCTTCCTACCCCAATTTCC	TTGGTCCTTAGCCACTCCTTC
Insr	mouse	ATGGGCTTCGGGAGAGGAT	GGATGTCCATACCAGGGCAC

Lipe	mouse	CCAGCCTGAGGGCTTACTG	CTCCATTGACTGTGACATCTCG
Mttp	mouse	CTCTTGGCAGTGCTTTTTCTCT	GAGCTTGTATAGCCGCTCATT
Pck1	mouse	GCATAACGGTCTGGACTTCT	TGATGACTGTCTTGCTTTCG
Pnpla2	mouse	GGATGAAAGAGCAGACGGGTAG	CGCAAGACAGTGGCACAGAG
Scd1	mouse	AAGATATTCACGACCCCACC	CAGCCGTGCCTTGTAAGTTC
Srebp1c	mouse	GCGCTACCGGTCTTCTATCA	GGATGTAGTCGATGGCCTTG
Tnf-α	mouse	CATCTTCTCAAAATTCGAGTGACAA	TGGGAGTAGACAAGGTACAACCC
Wdr6	mouse	GGGATCTCACCACGGCAATG	AAGGTCAGCCGCTGGTCTATG
α-Sma	mouse	CCCAGACATCAGGGAGTAATGG	TCTATCGGATACTTCAGCGTCA

Supplementary Data 10 | Antibodies used for western blots.

Antibodies	Source	Identifier
Mouse-anti-phospho-AKT (Ser473), dil:1/1000	Cell Signaling Technology	Cat# 12694; RRID AB_2797994
Rabbit-anti-AKT, dil:1/1000	Cell Signaling Technology	Cat# 9272; RRID AB_329827
Rabbit-anti-FASN, dil:1/2000	Cell Signaling Technology	Cat# 3180; RRID AB_2100796
Rabbit-anti-phospho-ACC (Ser79), dil:1/1000	Cell Signaling Technology	Cat# 11818; RRID AB_2687505
Rabbit-anti-ACC, dil:1/1000	Cell Signaling Technology	Cat# 3676; RRID AB_2219397
Rabbit-anti-CPT1A, dil:1/1000	Cell Signaling Technology	Cat# 12252; RRID AB_2797857
Rabbit-anti-HA, dil:1/1000	Cell Signaling Technology	Cat# 3724; RRID AB_1549585
Rabbit-anti-WDR6, dil:1/1000	Origene	Cat# TA322298; RRID AB_2924343
Mouse-anti-PPP1CA, dil:1/1000	Novus	Cat# NBP1-51600; RRID AB_11027838
Rabbit-anti-PPP1CC, dil:1/1000	Novus	Cat# NBP1-32858; RRID AB_2168096
Rabbit-anti-phospho-DNA-PK (Thr2609), dil:1/500	Novus	Cat# NBP1-02456; RRID AB_1522176
Mouse-anti-DNA-PK, dil:1/500	Novus	Cat# NBP2-22128; RRID AB_2910254
Mouse-anti-βActin, dil:1/7500	Proteintech	Cat# CL594-66009; RRID AB_2883475
Rabbit-anti-CD36, dil:1/1000	Proteintech	Cat# 18836-1-ap; RRID AB_10597244
Rabbit-anti-FLAG, dil:1/1000	Proteintech	Cat# 20543-1-AP; RRID AB_11232216
Mouse-anti-GAPDH, dil:1/7500	Proteintech	Cat# 60004-1-Ig; RRID AB_2107436

Mouse-anti-Lamin B1, dil:1/5000	Proteintech	Cat# 66095-1-Ig; RRID AB_11232208
Mouse-anti-SREBP1, dil:1/500	abcam	Cat# ab3259; RRID AB_303650
Rabbit-anti-PPP1CB, dil:1/2000	abcam	Cat# ab53315; RRID AB_2168274
Rabbit-anti-USF1, dil:1/1000	OriGene	Cat# TA327163; RRID AB_2910255
Mouse-anti-αSMA, dil:1/1000	abcam	Cat# ab7817; RRID AB_262054
Rabbit-anti-phospho-PPP1CB (Thr316), dil:1/500	This paper	N/A
Peroxidase-AffiniPure Goat Anti-Mouse IgG (H + L) antibody, dil:1/5000	Jackson ImmunoResearch Labs	Cat# 115-035-003; RRID AB_10015289
Peroxidase-AffiniPure Goat Anti-Rabbit IgG (H+L) antibody, dil:1/5000	Jackson ImmunoResearch Labs	Cat# 111-035-003; RRID AB_2313567
