# Supplementary material for: Upregulation of WDR6 drives hepatic de novo lipogenesis in insulin resistance in mice
Source: Nat Metab. 2023 Sep 21;5(10):1706–25. doi: 10.1038/s42255-023-00896-7 (PMC10590755; doi:10.1038/s42255-023-00896-7)
Supplement: Supplementary file 2 — Reporting Summary [file 42255_2023_896_MOESM2_ESM.pdf]

nature portfolio

Corresponding author(s): Jiajun Zhao, Tao Bo, Ling Gao

Last updated by author(s): Jul 26, 2023

Reporting Summary

Nature Portfolio wishes to improve the reproducibility of the work that we publish. This form provides structure for consistency and transparency in reporting. For further information on Nature Portfolio policies, see our Editorial Policies and the Editorial Policy Checklist.

Statistics

For all statistical analyses, confirm that the following items are present in the figure legend, table legend, main text, or Methods section.

n/a	Confirmed	
☐	☒	The exact sample size (*n*) for each experimental group/condition, given as a discrete number and unit of measurement
☐	☒	A statement on whether measurements were taken from distinct samples or whether the same sample was measured repeatedly
☐	☒	The statistical test(s) used AND whether they are one- or two-sided<br>*Only common tests should be described solely by name; describe more complex techniques in the Methods section.*
☐	☒	A description of all covariates tested
☐	☒	A description of any assumptions or corrections, such as tests of normality and adjustment for multiple comparisons
☐	☒	A full description of the statistical parameters including central tendency (e.g. means) or other basic estimates (e.g. regression coefficient) AND variation (e.g. standard deviation) or associated estimates of uncertainty (e.g. confidence intervals)
☐	☒	For null hypothesis testing, the test statistic (e.g. *F*, *t*, *r*) with confidence intervals, effect sizes, degrees of freedom and *P* value noted<br>*Give P values as exact values whenever suitable.*
☒	☐	For Bayesian analysis, information on the choice of priors and Markov chain Monte Carlo settings
☒	☐	For hierarchical and complex designs, identification of the appropriate level for tests and full reporting of outcomes
☒	☐	Estimates of effect sizes (e.g. Cohen's *d*, Pearson's *r*), indicating how they were calculated

Our web collection on statistics for biologists contains articles on many of the points above.

Software and code

Policy information about availability of computer code

Data collection	LightCycler480 Software (1.5.1.62); Nanodrop2000; PMOD software (v3.805); Amersham Imager 680 (v2.0.0); Analyst TF 1.7.1 Software; MutiQuant 3.0; LAS V4.9.
Data analysis	GraphPad Prism (v9.5.0); SPSS Statistics v25.0; AlphaView 3.2.2; AlphaSpace (v1.0); Glide (schrodinger release 2020-4); AmberTools17; UCSF Chimera; LigPlot; R Studio; Bowtie (v2.2.3); DESeq2 (1.32.0); ggplot2 (3.3.5); pheatmap (1.0.12); Hmisc package (version 3.16.0); ReactomePA (1.36.0); clusterProfiler (4.0.5);

For manuscripts utilizing custom algorithms or software that are central to the research but not yet described in published literature, software must be made available to editors and reviewers. We strongly encourage code deposition in a community repository (e.g. GitHub). See the Nature Portfolio guidelines for submitting code & software for further information.

Data

Policy information about availability of data

All manuscripts must include a data availability statement. This statement should provide the following information, where applicable:

- Accession codes, unique identifiers, or web links for publicly available datasets
- A description of any restrictions on data availability
- For clinical datasets or third party data, please ensure that the statement adheres to our policy

The data that support the findings of this study are available from the corresponding author upon reasonable request. RNA-seq data have been deposited at Gene

Expression Omnibus (GEO) and is publicly available as of the data of publication: Fig. 1c, Supplementary Data Fig. 1 (GSE205460) and Extended Data Fig. 8 (GSE205459). Proteomics and Phosphoproteomics dataset have been deposited at the ProteomeXchange Consortium via the PRIDE partner repository and are publicly available from the date of publication: PXD034367 and PXD034542. AlphaFold Protein Structure Database: https://alphafold.com/entry/Q9NNW5.

Human research participants

Policy information about studies involving human research participants and Sex and Gender in Research.

Reporting on sex and gender	To carry out the correlation between the protein levels of WDR6 and FASN/ liver TAG contents, we recruited 20 patient who with hepatobiliary tumors that had undergone liver resection in Shandong Provincial Hospital between 2016 and 2021. A total of 12 female participants and 8 male participants were used for the analysis.
Population characteristics	20 participants aged 55.70±14.63 were Chinese individuals.
Recruitment	Human liver samples were obtained from patients who with hepatobiliary tumors that had undergone liver resection in Shandong Provincial Hospital between 2016 and 2021. All participants took part voluntarily.
Ethics oversight	All patients provided written informed consents before sample collection. The procedures were approved by the Ethics Committee of the Shandong Provincial Hospital (NO. 2019-070).

Note that full information on the approval of the study protocol must also be provided in the manuscript.

Field-specific reporting

Please select the one below that is the best fit for your research. If you are not sure, read the appropriate sections before making your selection.

☒ Life sciences ☐ Behavioural & social sciences ☐ Ecological, evolutionary & environmental sciences

For a reference copy of the document with all sections, see nature.com/documents/nr-reporting-summary-flat.pdf

Life sciences study design

All studies must disclose on these points even when the disclosure is negative.

Sample size	The sample size of our animal experiments was determined based on previous experiments and to minimize the use in accordance with animal care guidelines. Additional details regarding sample size can be found in the corresponding figure legends.<br>1. Guo Y, Zhao M, Bo T, et al. Blocking FSH inhibits hepatic cholesterol biosynthesis and reduces serum cholesterol. Cell Res. 2019;29(2):151-166. doi:10.1038/s41422-018-0123-6. PMID: 30559440.<br>2. Wang X, Du H, Shao S, et al. Cyclophilin D deficiency attenuates mitochondrial perturbation and ameliorates hepatic steatosis. Hepatology. 2018;68(1):62-77. doi:10.1002/hep.29788. PMID: 29356058.<br>3. Yang C, Lu M, Chen W, et al. Thyrotropin aggravates atherosclerosis by promoting macrophage inflammation in plaques [published correction appears in J Exp Med. 2022 Feb 7;219(2):]. J Exp Med. 2019;216(5):1182-1198. doi:10.1084/jem.20181473. PMID: 30940720.
Data exclusions	No samples were excluded in animals study.
Replication	All experiments were performed at least triplicate and represent reproducible findings. Additional details regarding replication and sample size can be found in the corresponding figure legends.
Randomization	For in vitro experiments, all mice were assigned to different groups according to a randomized block experimental design. For in vitro experiments, cells were randomly allocated into different groups.
Blinding	Operators for animal and cell experiments were blinded. Investigators were blinded to groups allocation during data analysis.

Reporting for specific materials, systems and methods

We require information from authors about some types of materials, experimental systems and methods used in many studies. Here, indicate whether each material, system or method listed is relevant to your study. If you are not sure if a list item applies to your research, read the appropriate section before selecting a response.

Materials & experimental systems

n/a	Involved in the study
☐	☒ Antibodies
☐	☒ Eukaryotic cell lines
☒	☐ Palaeontology and archaeology
☐	☒ Animals and other organisms
☒	☐ Clinical data
☒	☐ Dual use research of concern

Methods

n/a	Involved in the study
☒	☐ ChIP-seq
☒	☐ Flow cytometry
☒	☐ MRI-based neuroimaging

Antibodies

Antibodies used

Cell Signaling Technology antibodies: Mouse-anti-phospho-AKT (Ser473) (Cat# 12694); Rabbit-anti-AKT (Cat# 9272); Rabbit-anti-FASN (Cat# 3180); Rabbit-anti-phospho-ACC (Ser79) (Cat# 11818); Rabbit-anti-ACC (Cat# 3676); Rabbit-anti-CPT1A (Cat# 12252); Rabbit-anti-HA (Cat# 3724).
Origene antibodies: Rabbit-anti-WDR6 (Cat# TA322298); Rabbit-anti-USF1 (Cat# TA327163).
Novus antibodies: Mouse-anti-PPP1CA (Cat# NBP1-51600); Rabbit-anti-PPP1CC (Cat# NBP1-32858); Rabbit-anti-phospho-DNA-PK (Thr2609) (Cat# NBP1-02456); Mouse-anti-DNA-PK (Cat# NBP2-22128).
Proteintech antibodies: Mouse-anti-Beta Actin (Cat# CL594-66009); Rabbit-anti-CD36 (Cat# 18836-1-ap); Rabbit-anti-FLAG (Cat# 20543-1-AP); Mouse-anti-GAPDH (Cat# 60004-1-Ig); Mouse-anti-Lamin B1 (Cat# 66095-1-Ig).
Abcam antibodies: Mouse-anti-SREBP1 (Cat# ab3259); Rabbit-anti-PPP1CB (Cat# ab53315); Mouse-anti-αSMA (Cat# ab7817).
Secondary antibodies from Jackson ImmunoResearch Labs: Peroxidase-AffiniPure Goat Anti-Mouse IgG (H + L) antibody (Cat# 115-035-003); Peroxidase-AffiniPure Goat Anti-Rabbit IgG (H+L) antibody (Cat# 111-035-003).

Validation

The antibodies used in the study are validated via manufacturers and the related information are available on the website:
Anti-phospho-AKT (Ser473) was validated for WB in various cell lines (https://www.cellsignal.cn/products/primary-antibodies/phospho-akt-ser473-d9w9u-mouse-mab/12694?site-search-ype=Products&N=4294956287&Ntt=12694&fromPage=plp&_requestid=7053621).
Anti-AKT was validated for WB with knockdown in cell lines (https://www.cellsignal.cn/products/primary-antibodies/akt-antibody/9272?site-search-type=Products&N=4294956287&Ntt=9272&fromPage=plp&_requestid=7049436).
Anti-FASN was validated for WB in various cell lines (https://www.cellsignal.cn/products/primary-antibodies/fatty-acid-synthase-c20g5-rabbit-mab/3180?site-search-type=Products&N=4294956287&Ntt=3180&fromPage=plp&_requestid=7049687).
Anti-phospho-ACC (Ser79) was validated for WB with oligomycin in cell lines (https://www.cellsignal.cn/products/primary-antibodies/phospho-acetyl-coa-carboxylase-ser79-d7d11-rabbit-mab/11818?site-search-type=Products&N=4294956287&Ntt=11818&fromPage=plp&_requestid=7049848).
Anti-ACC was validated for WB in various cell lines (https://www.cellsignal.cn/products/primary-antibodies/acetyl-coa-carboxylase-c83b10-rabbit-mab/3676?site-search-type=Products&N=4294956287&Ntt=3676&fromPage=plp&_requestid=7049984).
Anti-CPT1A was validated for WB in various cell lines (https://www.cellsignal.cn/products/primary-antibodies/cpt1a-d3b3-rabbit-mab/12252?site-search-type=Products&N=4294956287&Ntt=12252&fromPage=plp&_requestid=7050049).
Anti-HA was validated for WB with HA-FoxO4 overexpression in Hela cell (https://www.cellsignal.cn/products/primary-antibodies/ha-tag-c29f4-rabbit-mab/3724?site-search-type=Products&N=4294956287&Ntt=3724&fromPage=plp&_requestid=7050172).
Anti-WDR6 was validated for WB in various cell lines (https://www.origene.com.cn/search?q=TA322298).
Anti-USF1 was validated for WB in various cell lines (https://www.origene.com.cn/search?q=TA327163).
Anti-PPP1CA was validated for WB in various cell lines (PP1 alpha/PPP1A Antibody (6D1) (NBP1-51600): Novus Biologicals).
Anti-PPP1CC was validated for WB in various cell lines (https://www.novusbio.com/products/protein-phosphatase-1c-gamma-antibody_nbp1-32858).
Anti-αSMA was validated for WB in various cell lines (https://www.abcam.cn/products/primary-antibodies/alpha-smooth-muscle-actin-antibody-1a4-ab7817.html).
Anti-phospho-DNA-PK (Thr2609) was validated for WB and IHC in various cell lines (DNA-PKcs [p Thr2609] Antibody - BSA Free (NBP1-02456): Novus Biologicals). Refs. PMID 22666473, 23691119.
Anti-DNA-PK was validated for WB in various cell lines (https://www.novusbio.com/products/dna-pkcs-antibody-3h6_nbp2-22128).
Anti-Beta Actin was validated for WB in various cell lines (https://www.ptgcn.com/products/beta-Actin-Antibody-CL594-66009.htm).
Anti-CD36 was validated for WB in various cell lines (https://www.ptgcn.com/products/CD36-Antibody-18836-1-AP.htm).
Anti-FLAG was validated for WB in various cell lines (https://www.ptgcn.com/products/Flag-Tag-Antibody-20543-1-AP.htm).
Anti-GAPDH was validated for WB in various cell lines (https://www.ptgcn.com/products/GAPDH-Antibody-60004-1-Ig.htm).
Anti-Lamin B1 was validated for WB in various cell lines (https://www.ptgcn.com/products/LMNB1-Antibody-66095-1-Ig.htm).
Anti-SREBP1 was validated for WB in various cell lines (https://www.abcam.cn/srebp1-antibody-2a4-ab3259.html).
Anti-PPP1CB was validated for WB and IHC in various cell lines (https://www.abcam.cn/ppp1cb-antibody-ep1804y-ab53315.html).
Rabbit-anti-phospho-PPP1CB (Thr316) were generated by Shanghai Immune Biotech and has been validated using western blotting of mice liver tissues (Figure 4, 6, 7 and Extended Data Figures 10). The dilution factor was 1000-fold.

Eukaryotic cell lines

Policy information about cell lines and Sex and Gender in Research

Cell line source(s)

HEK293 (#GNHu 43), HepG2 (#SCSP-510) and Hepa1-6 (TCM39) cells were obtained from Cell library of the Chinese Academy of Sciences.

Authentication

HEK293, HepG2 and Hepa1-6 cells were authenticated by growth properties, morphology and STR profiling.

Mycoplasma contamination	All cell lines tested negative for mycoplasma contamination
Commonly misidentified lines (See ICLAC register)	None.

Animals and other research organisms

Policy information about studies involving animals; ARRIVE guidelines recommended for reporting animal research, and Sex and Gender in Research

Laboratory animals	WDR6-WKO (Cyagen Biosciences), Wdr6fl/fl (GemPharmatech), Srebp1c-/- (kindly provided by Prof. Youfei Guan), Usf1-/- (Strain ID: T043964), Albpro::Cre (Strain ID: T003814), Wdr6-ki fl/fl (GemPharmatech) strains used in this study were male on a C57BL/6J background. Mice ranging from 7-24 weeks age were used for all the experiments. The mice were maintained on a 12-h light/dark cycle at 22 ± 0.5°C, 50-60% humidity, with food and water provided ad libitum.
Wild animals	No wild animals were included in this study.
Reporting on sex	Our study utilized both males and females. No sex predilection has been observed.
Field-collected samples	No field-collected samples included.
Ethics oversight	All performed procedures on animals have been approved by the Ethics Committee of the Shandong Provincial Hospital.

Note that full information on the approval of the study protocol must also be provided in the manuscript.
