# Supplementary figures and images for: Upregulation of WDR6 drives hepatic de novo lipogenesis in insulin resistance in mice
Source: Nat Metab. 2023 Sep 21;5(10):1706–25. doi: 10.1038/s42255-023-00896-7 (PMC10590755; doi:10.1038/s42255-023-00896-7)

Supplementary Data 4c

kDa

100  
70  
55

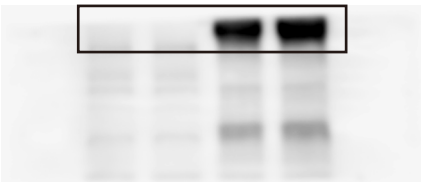

WDR6-FLAG

40

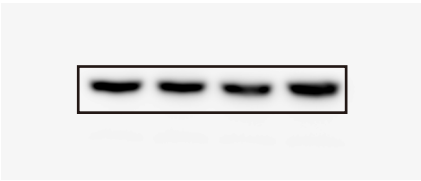

$\beta$ -Actin

Supplement: Supplementary file 4 — Unprocessed western blots. [file 42255_2023_896_MOESM4_ESM.pdf]

**Fig 1b**

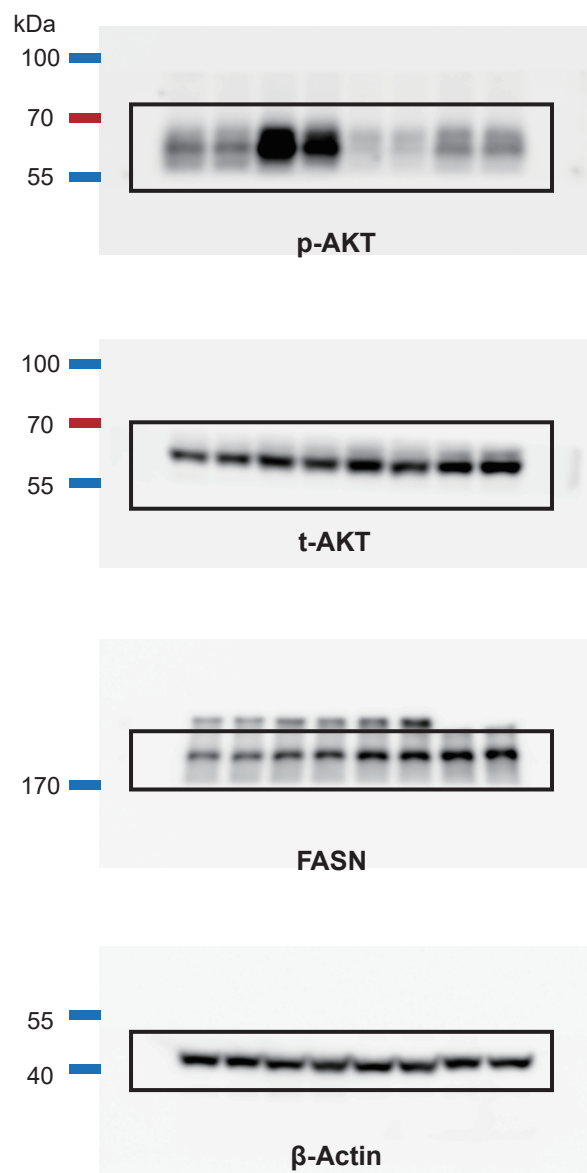

**Fig 1e**

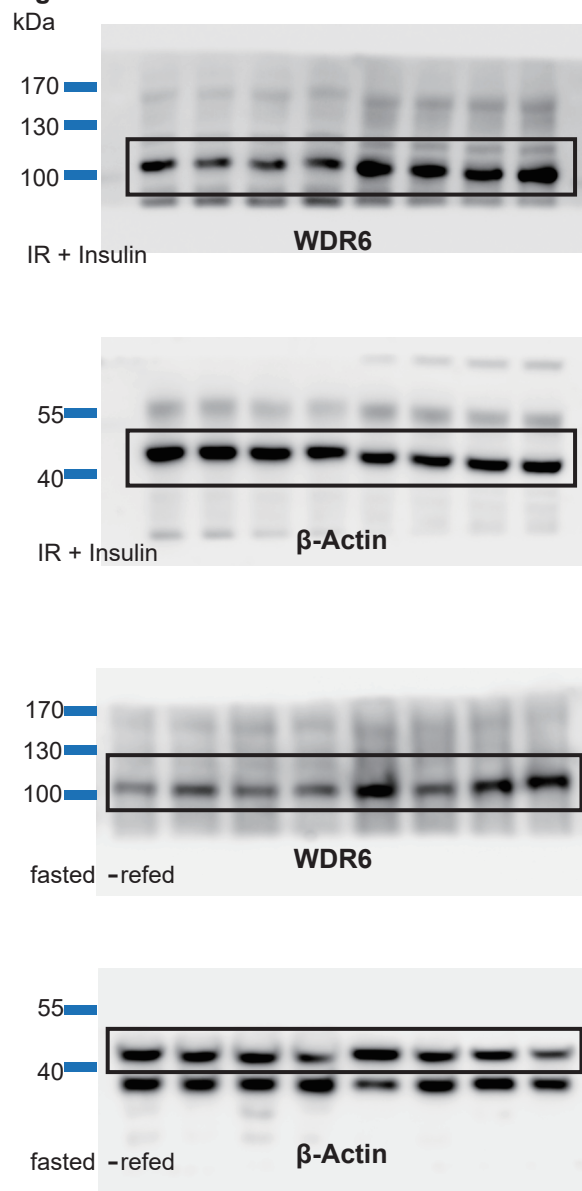

**Fig 1f**

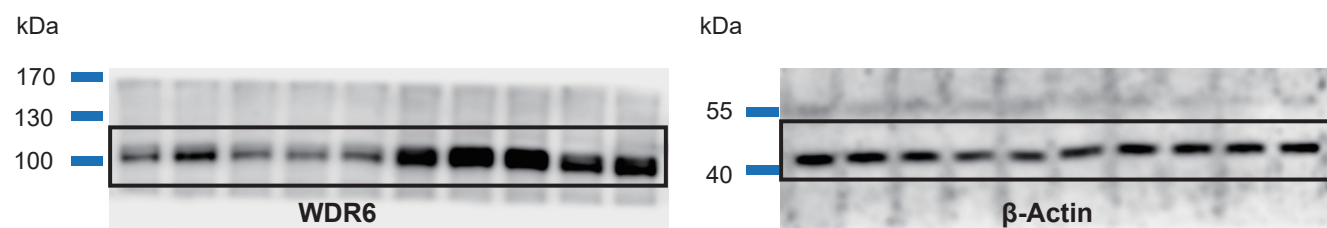

Supplement: Supplementary file 6 — Unprocessed western blots. [file 42255_2023_896_MOESM6_ESM.pdf]

Fig 3g

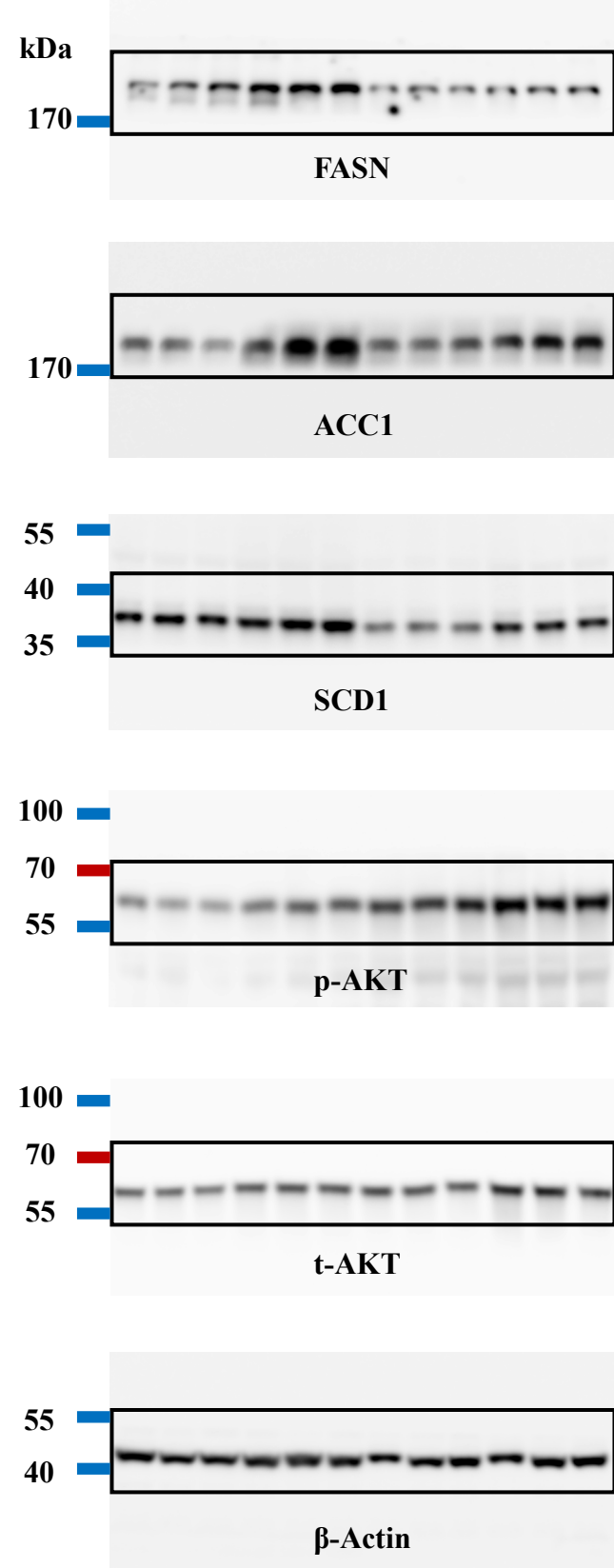

Fig 3j

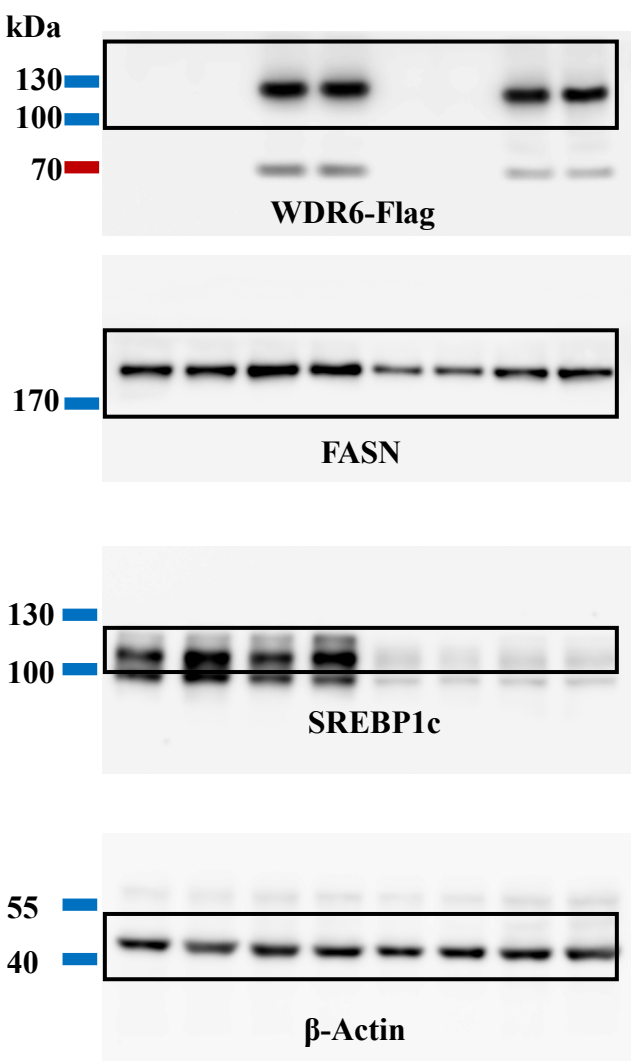

Supplement: Supplementary file 9 — Unprocessed western blots. [file 42255_2023_896_MOESM9_ESM.pdf]

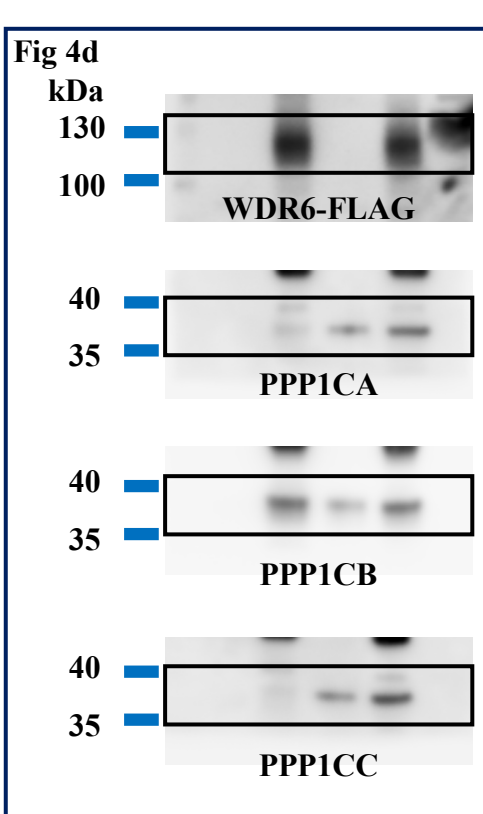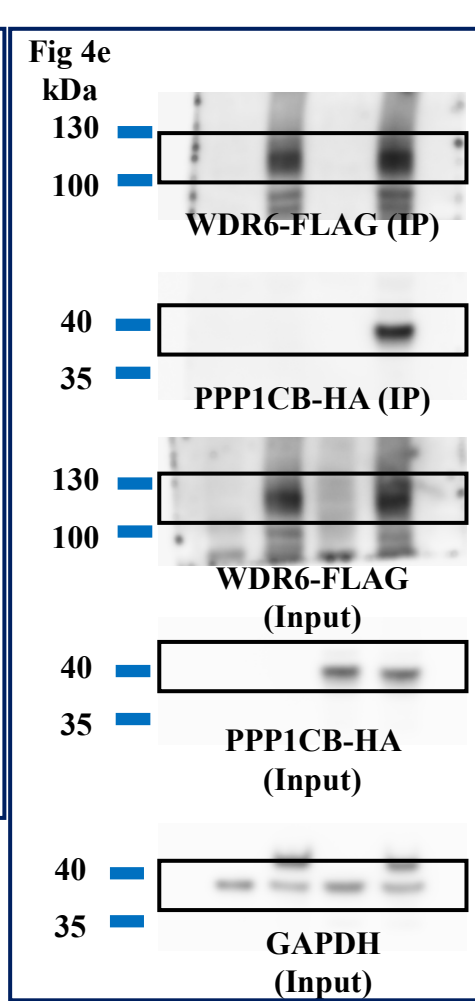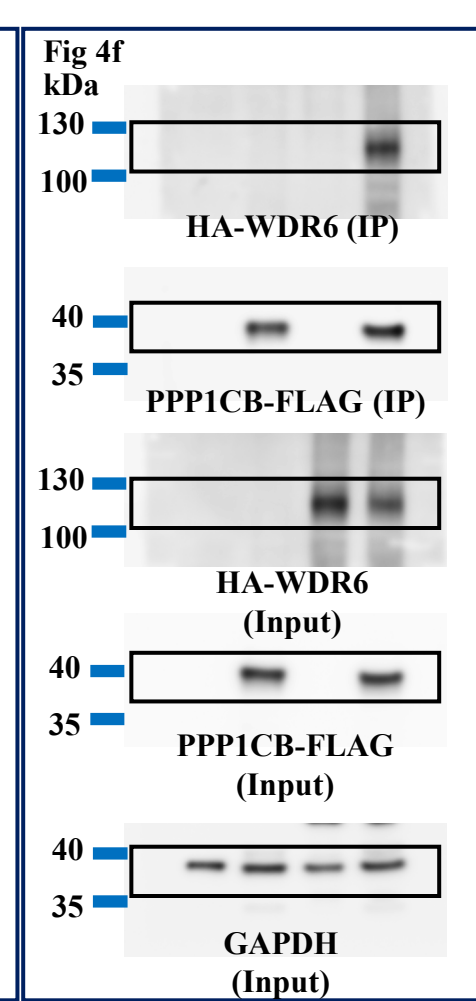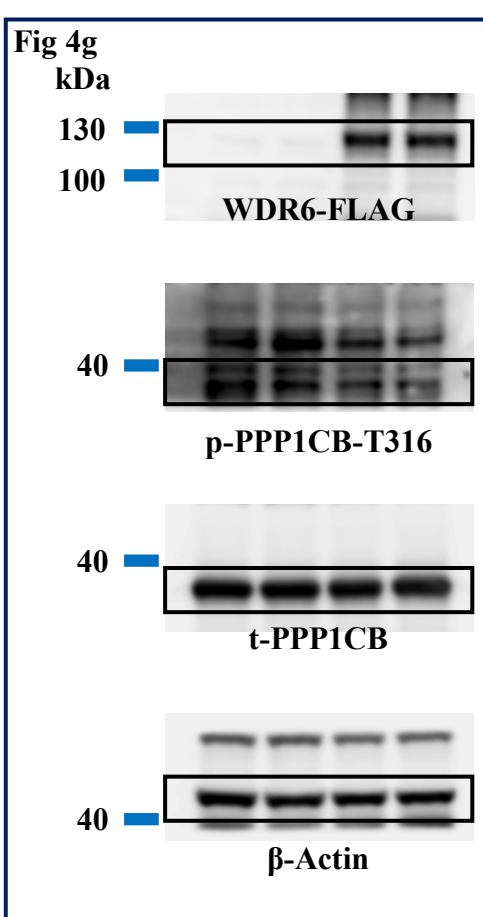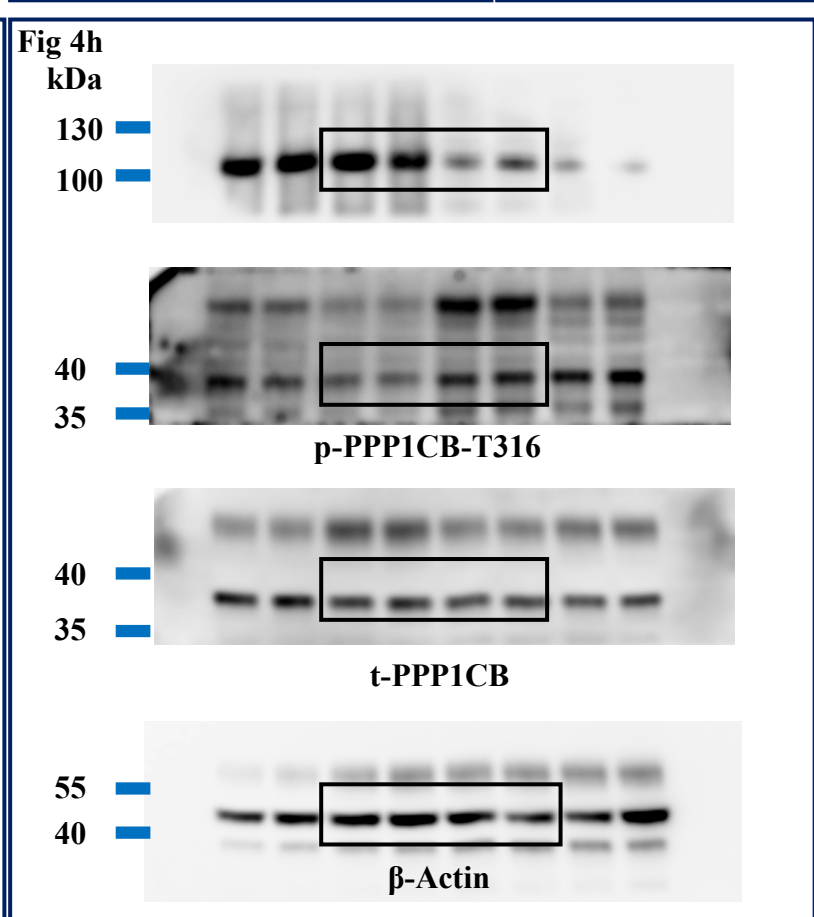

**Fig 4i**

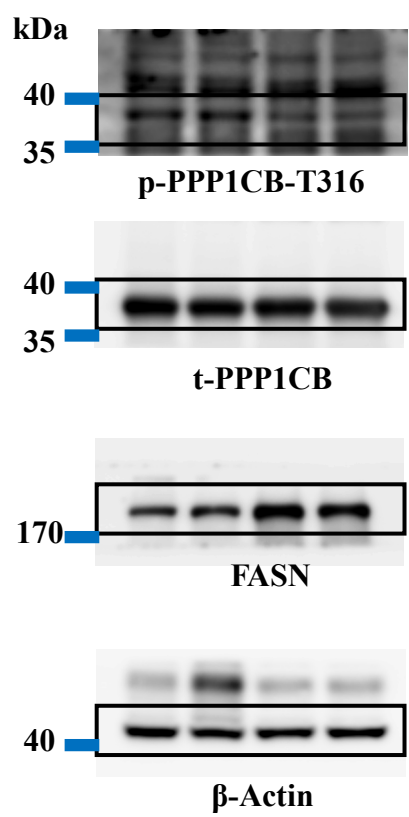

**Fig 4j**

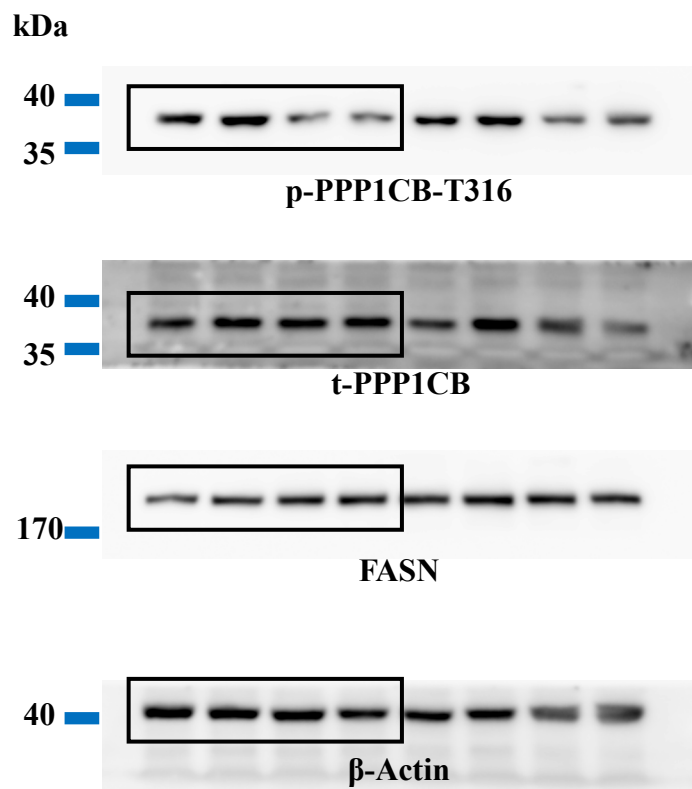

**Fig 4k**

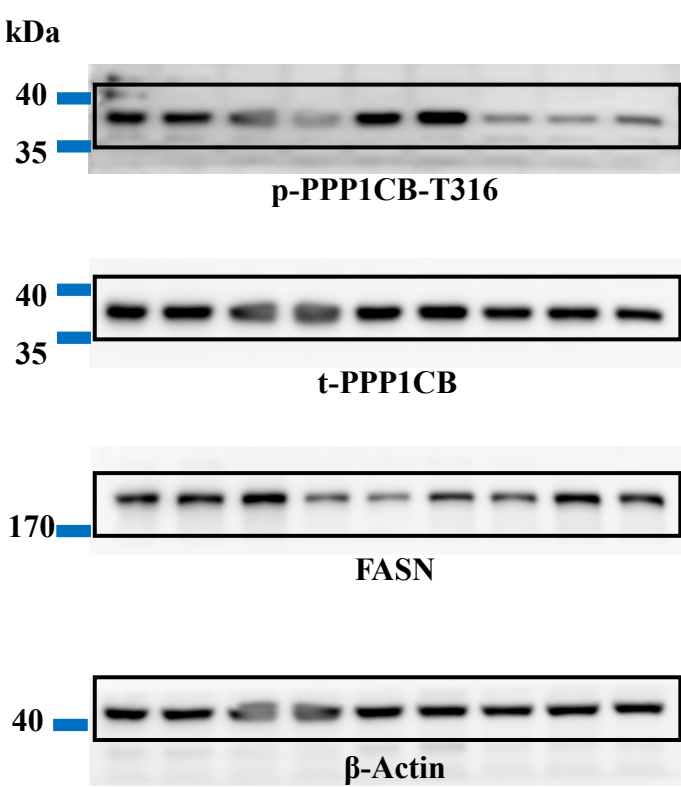

Supplement: Supplementary file 11 — Unprocessed western blots. [file 42255_2023_896_MOESM11_ESM.pdf]

**Fig 5a**

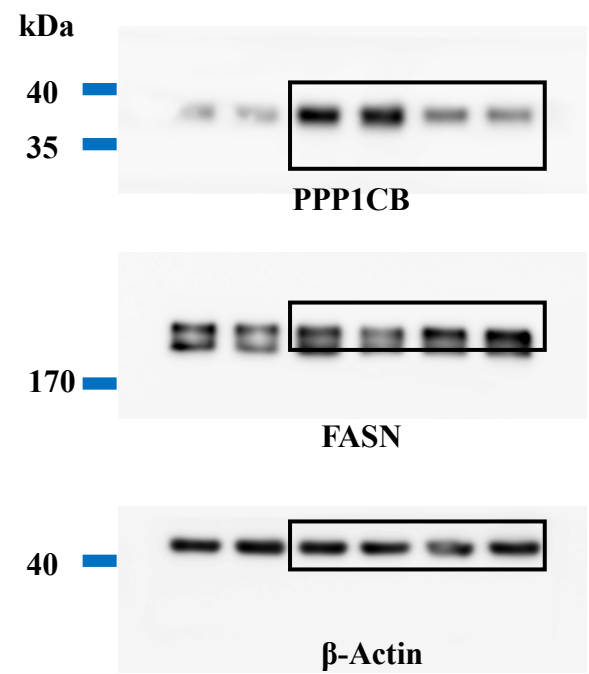

**Fig 5b**

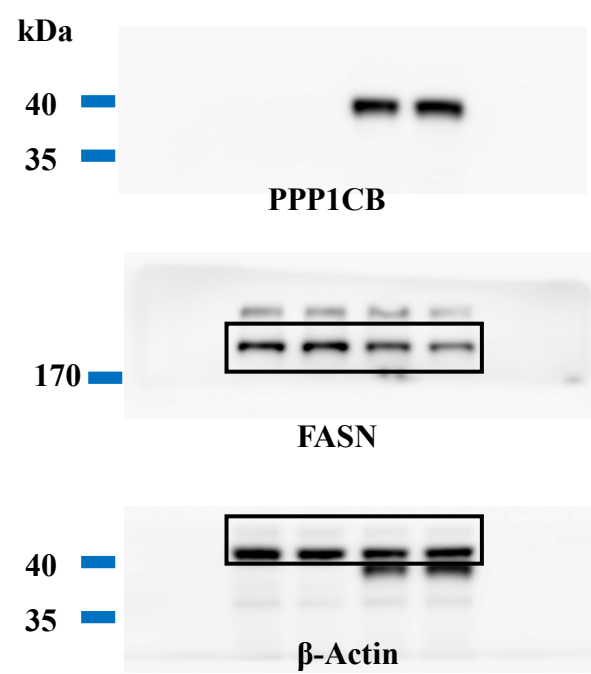

**Fig 5c**

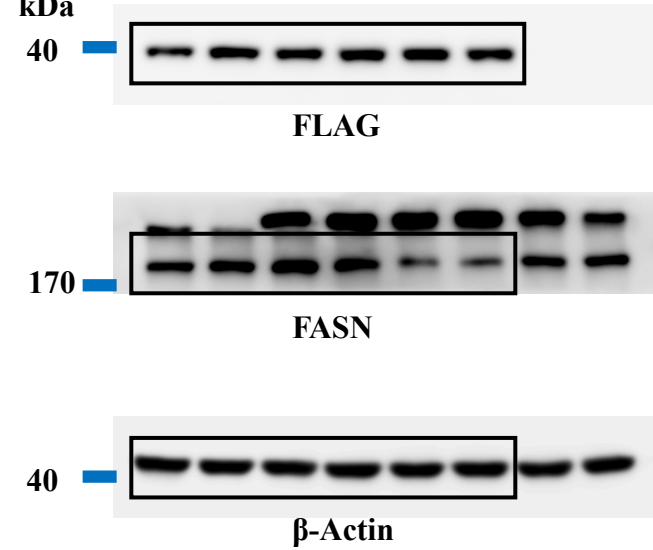

**Fig 5f**

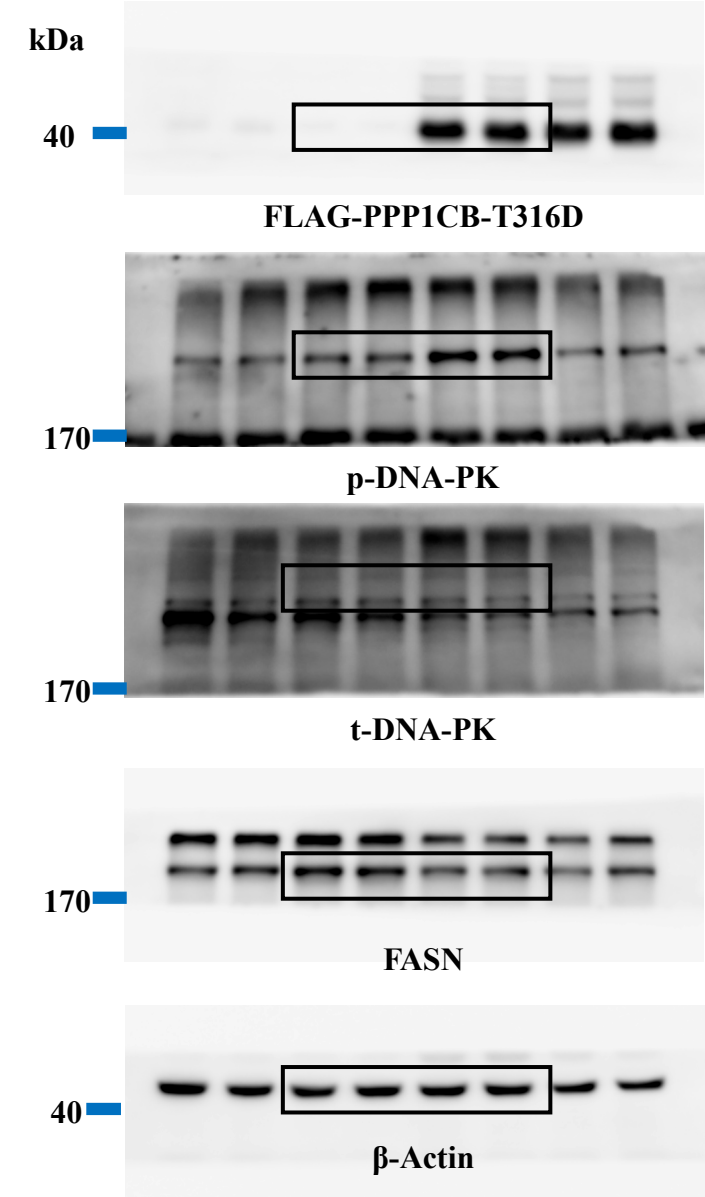

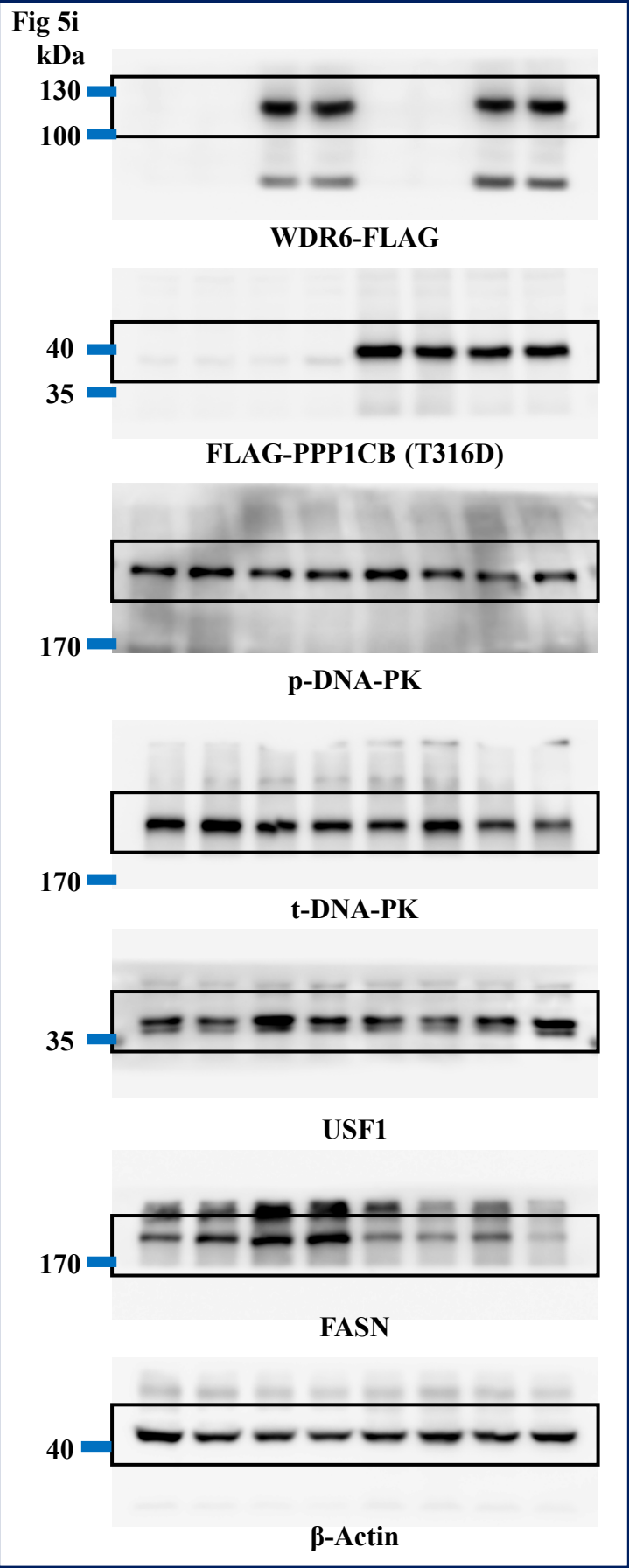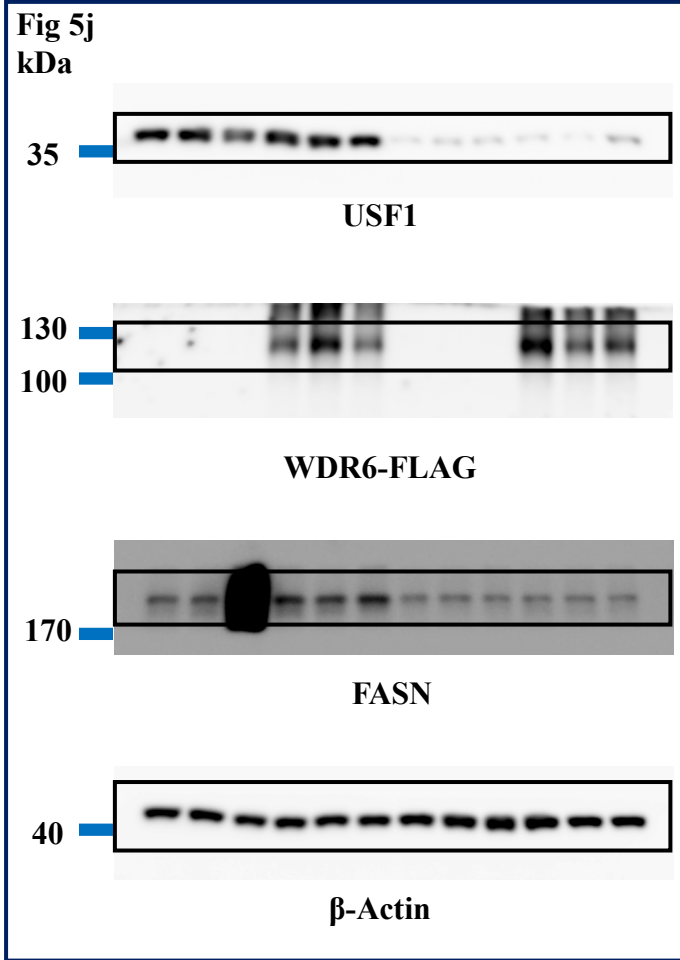

Supplement: Supplementary file 13 — Unprocessed western blots. [file 42255_2023_896_MOESM13_ESM.pdf]

**Fig 6c**

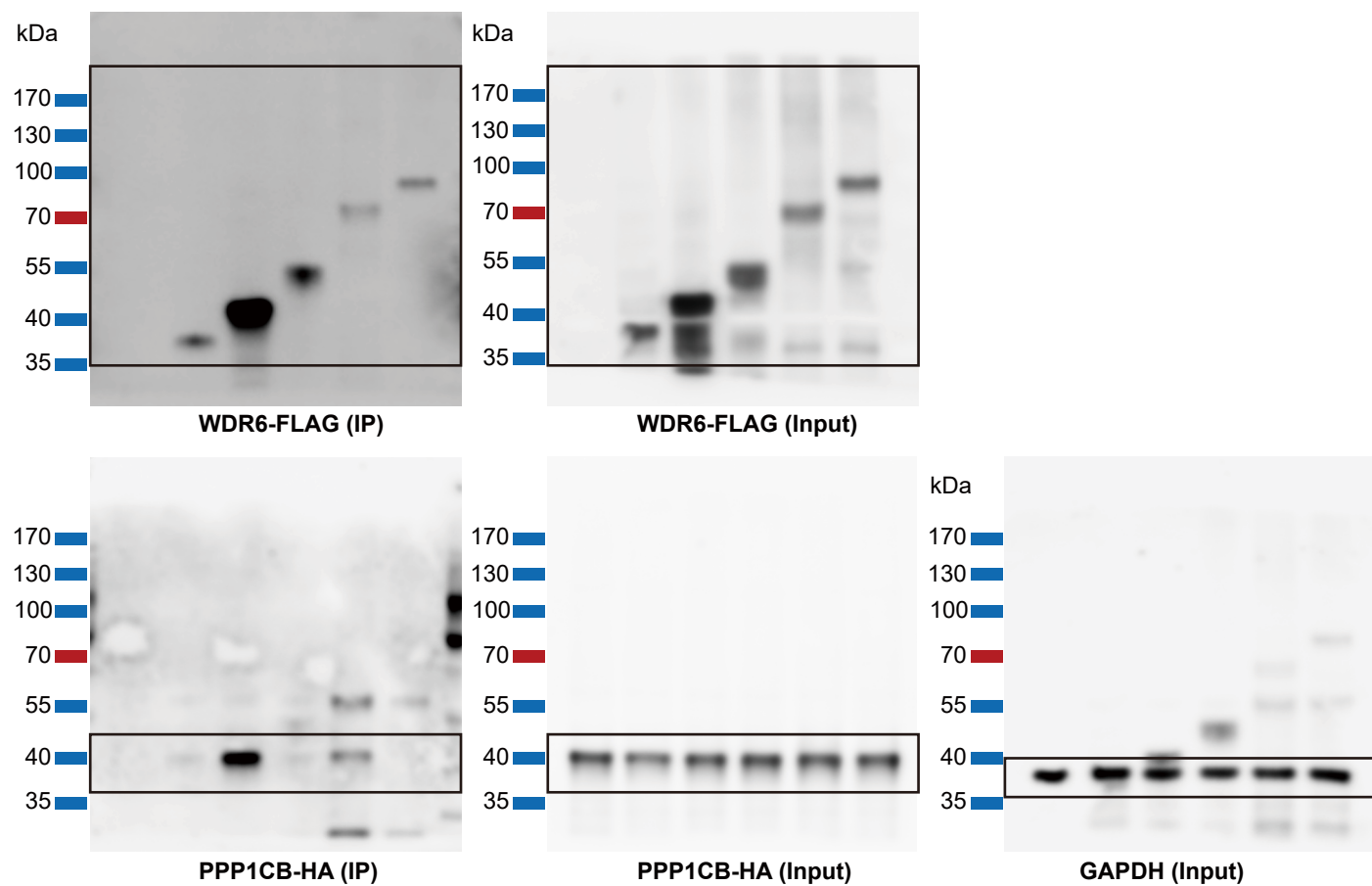

**Fig 6e**

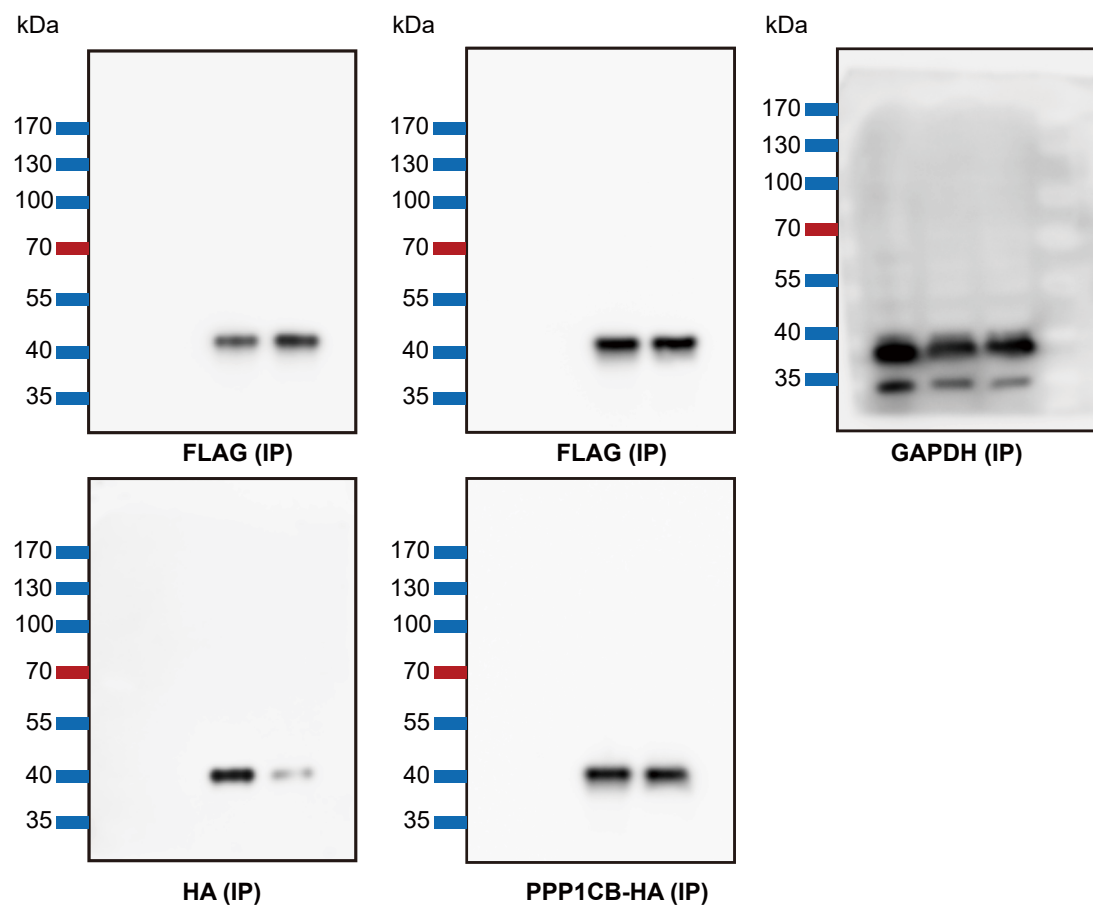

**Fig 6f**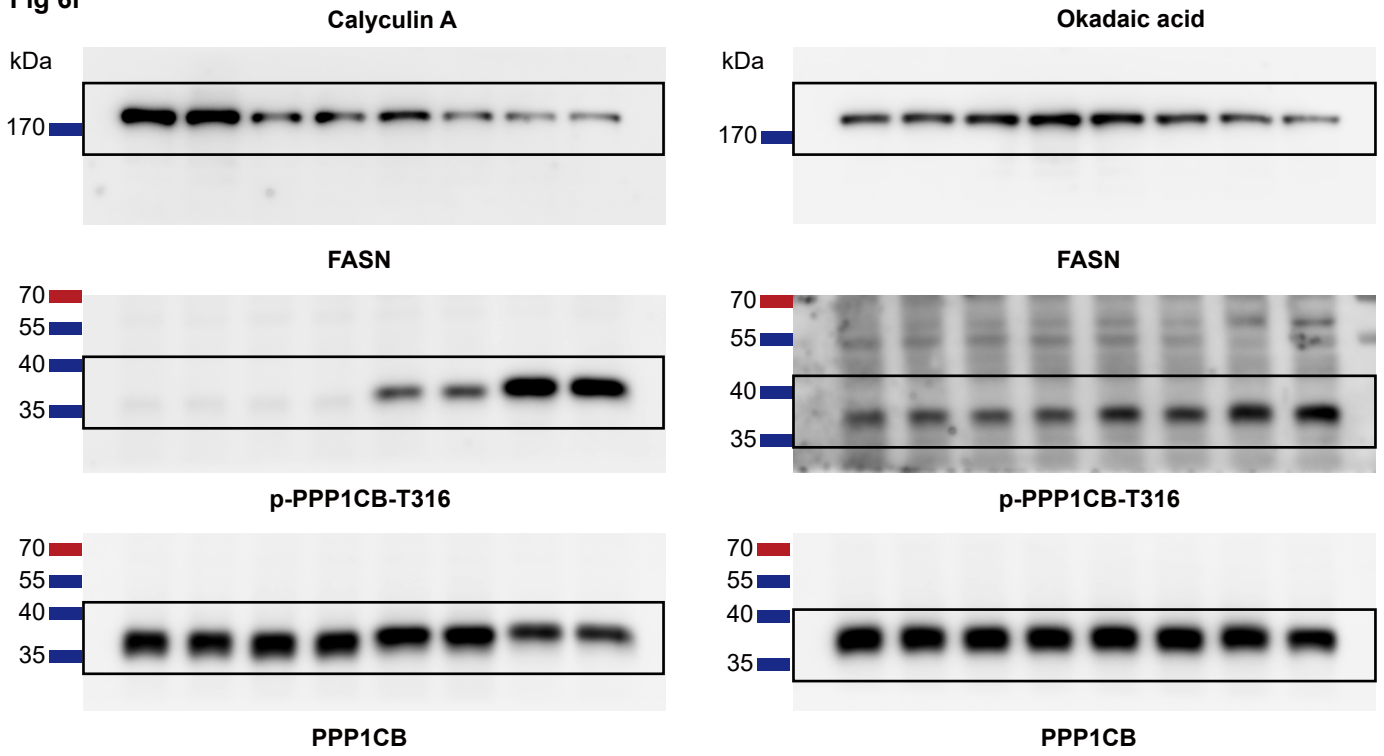**Fig 6g**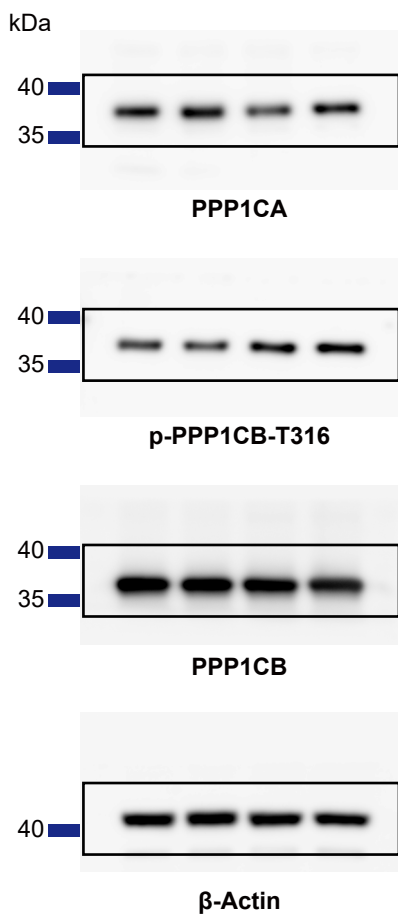**Fig 6h**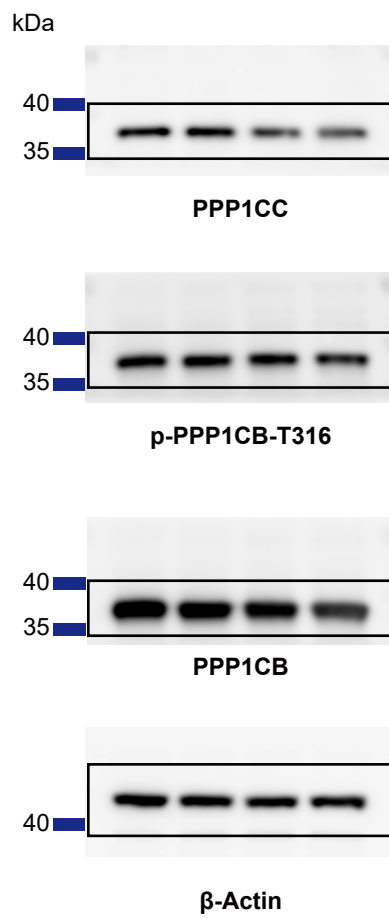**Fig 6i**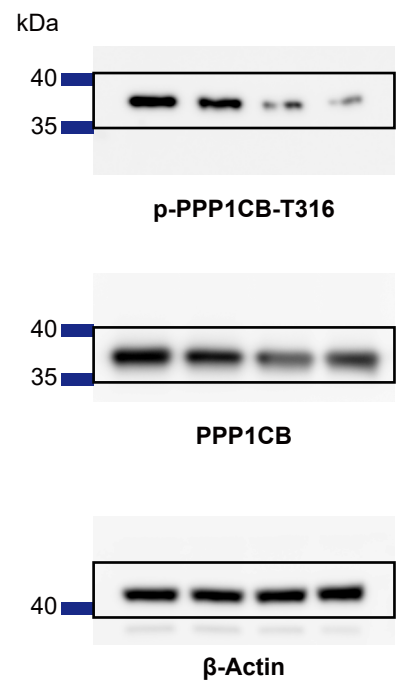

**Fig 6j**

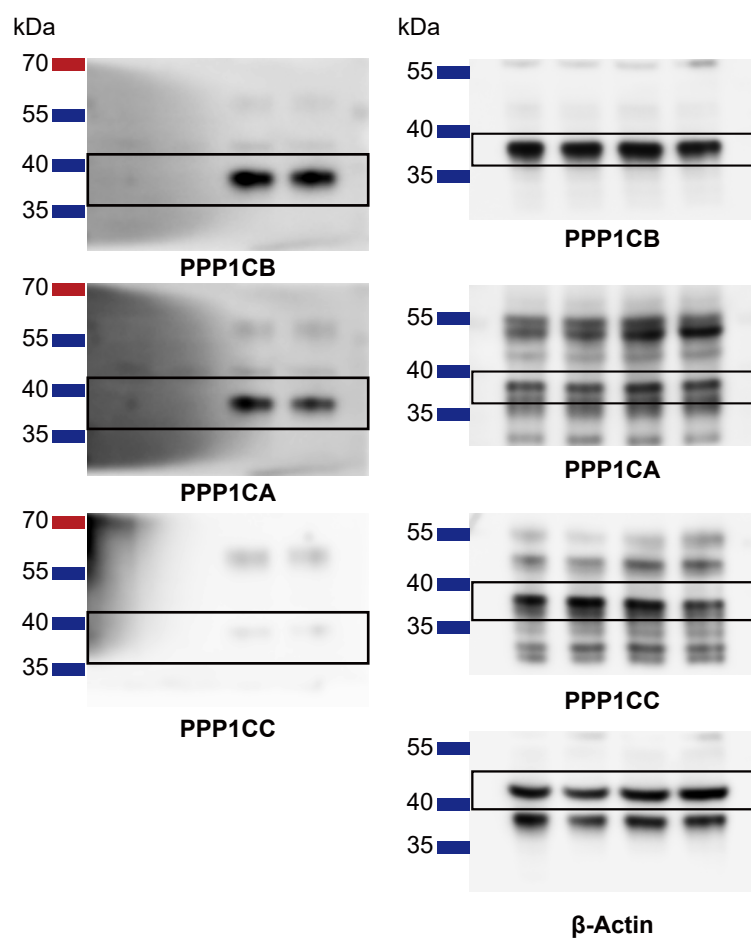

Supplement: Supplementary file 15 — Unprocessed western blots. [file 42255_2023_896_MOESM15_ESM.pdf]

**Fig 7a**

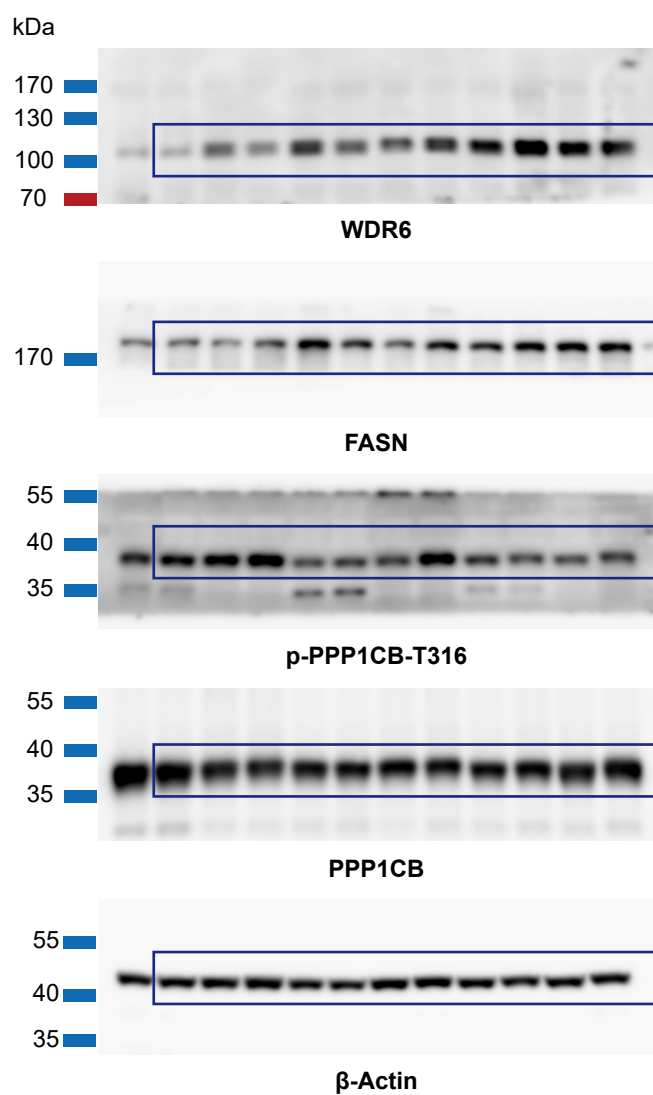

**Fig 7i**

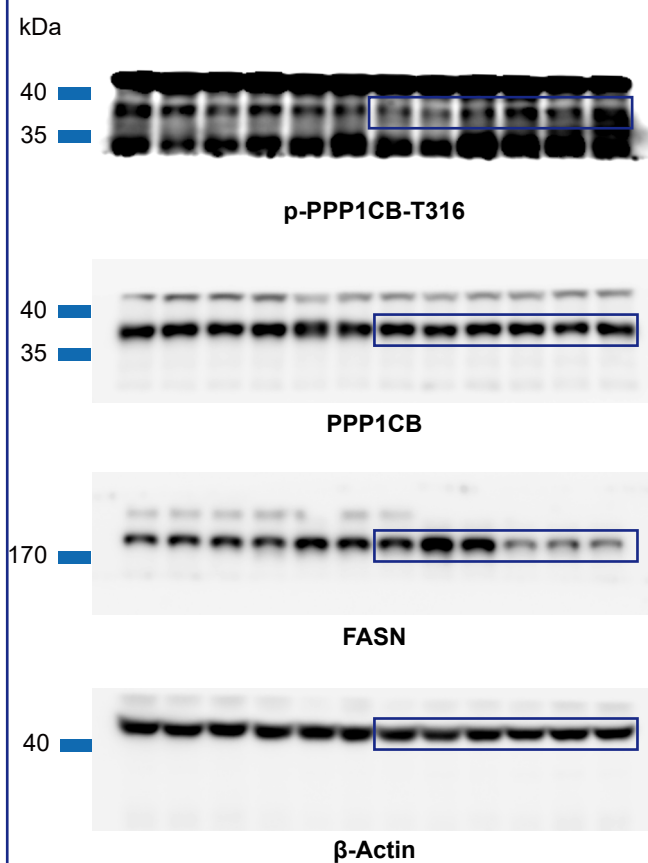

**Fig 7m**

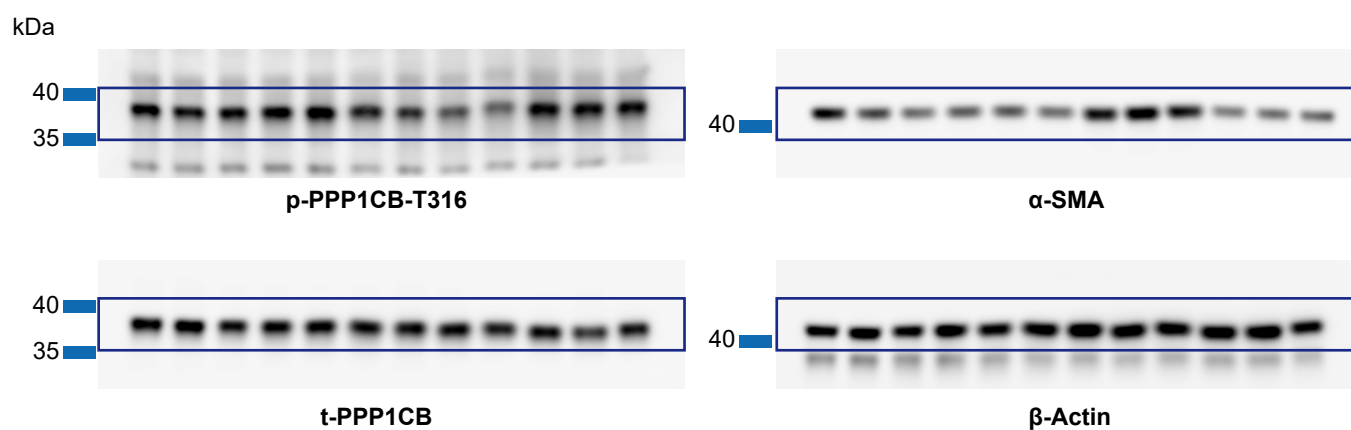

Supplement: Supplementary file 17 — Unprocessed western blots. [file 42255_2023_896_MOESM17_ESM.pdf]

Extended Data Fig. 1b

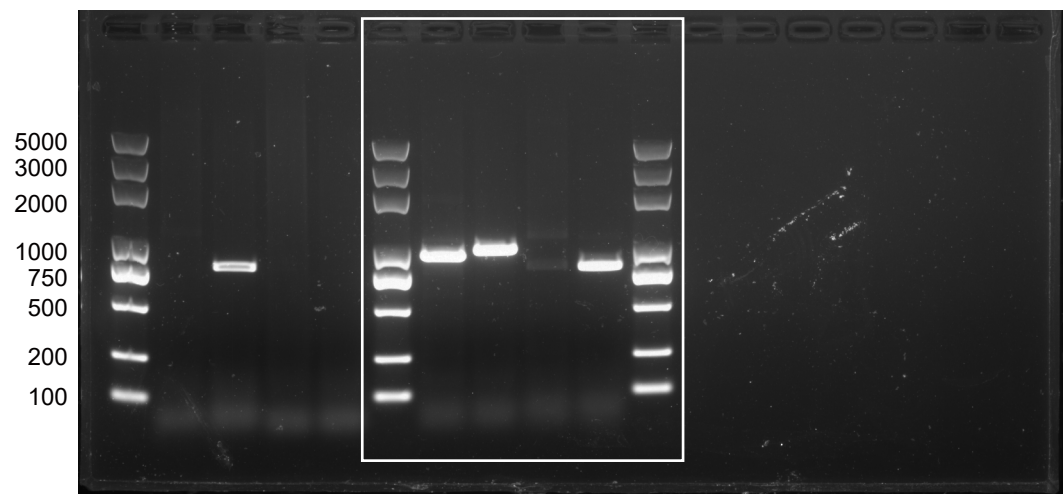

Extended Data Fig. 1c

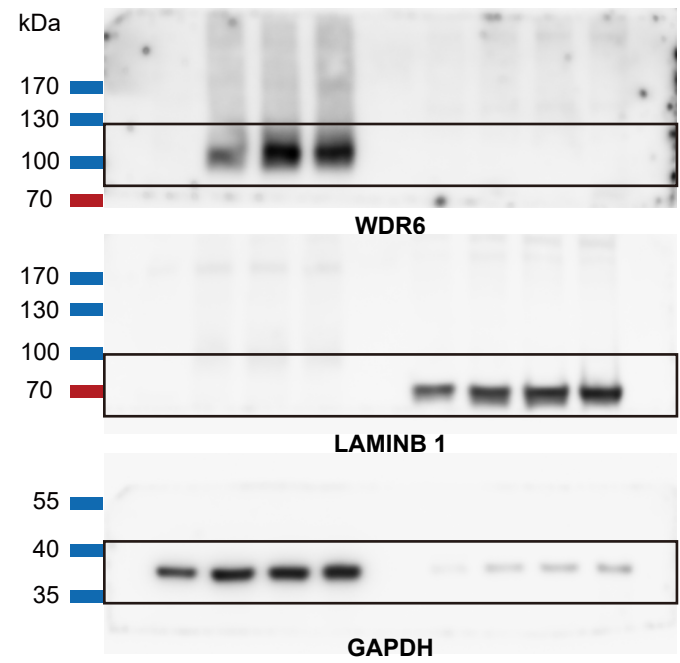

Extended Data Fig. 1e

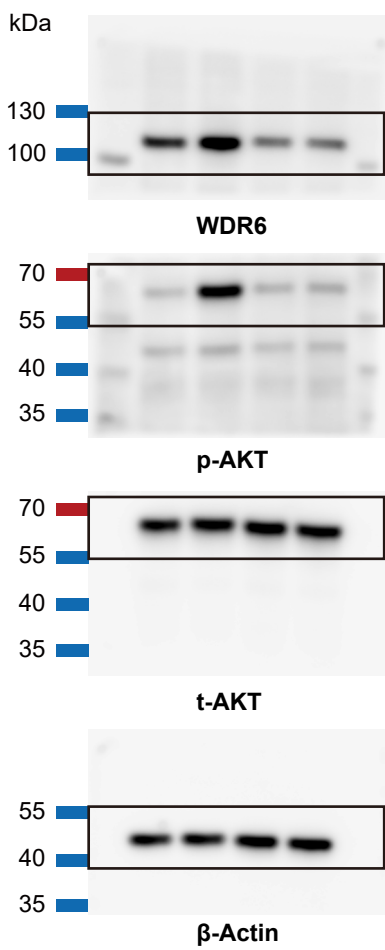

Extended Data Fig. 1f

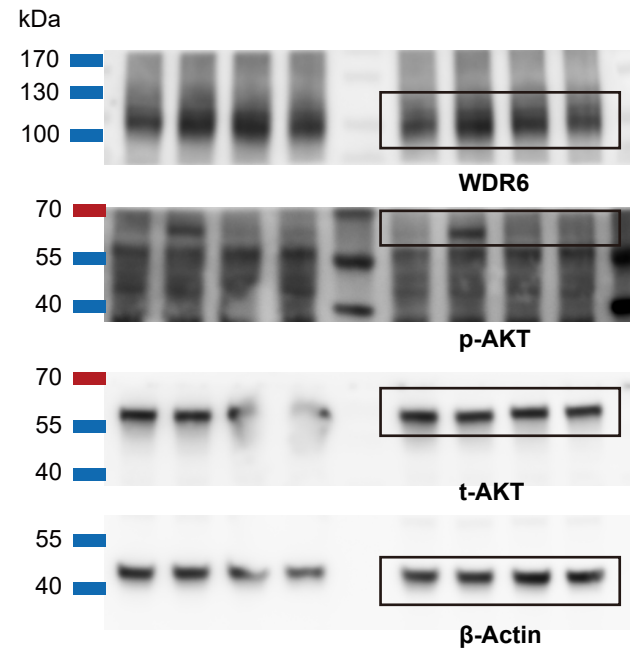

Supplement: Supplementary file 19 — Unprocessed western blots. [file 42255_2023_896_MOESM19_ESM.pdf]

Extended Data Fig. 2b

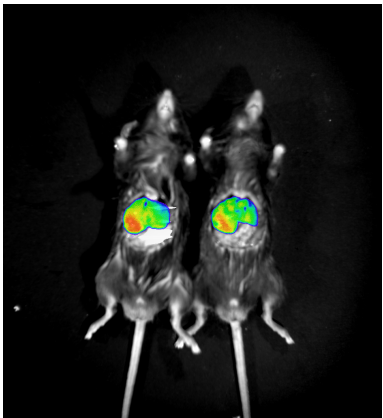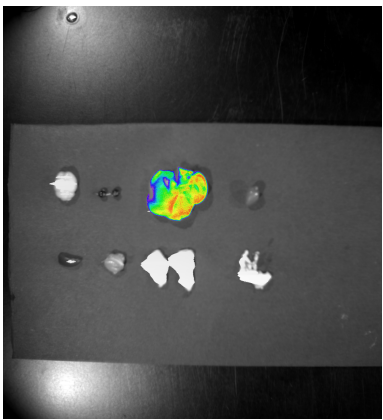

Extended Data Fig. 2d

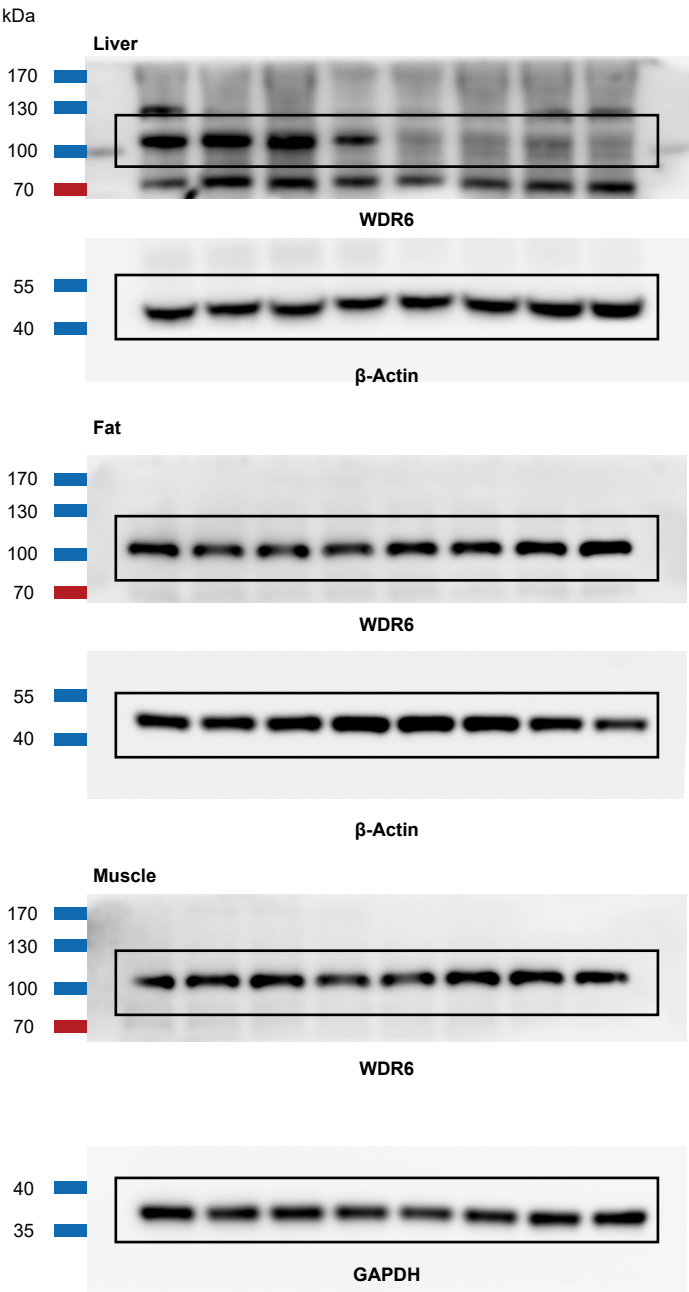

Supplement: Supplementary file 21 — Unprocessed western blots. [file 42255_2023_896_MOESM21_ESM.pdf]

Extended Data Fig. 4b

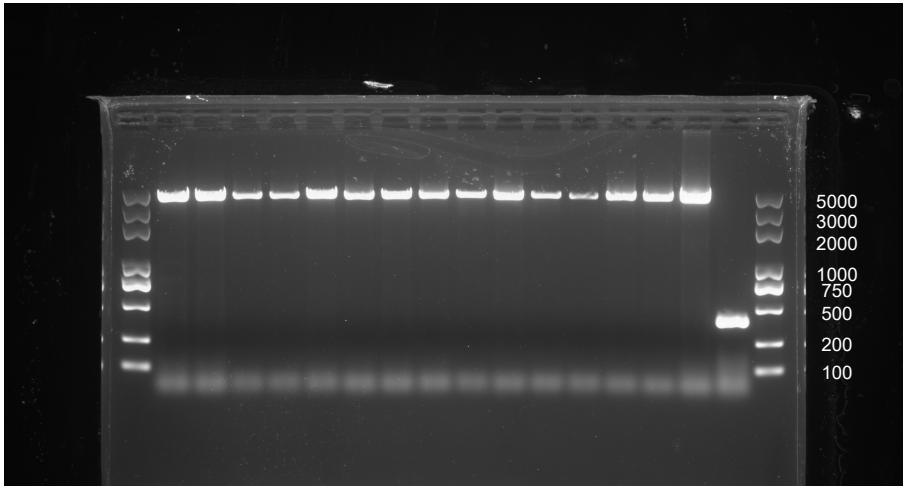

Supplement: Supplementary file 24 — Unprocessed western blots. [file 42255_2023_896_MOESM24_ESM.pdf]

Extended Data Fig. 5a

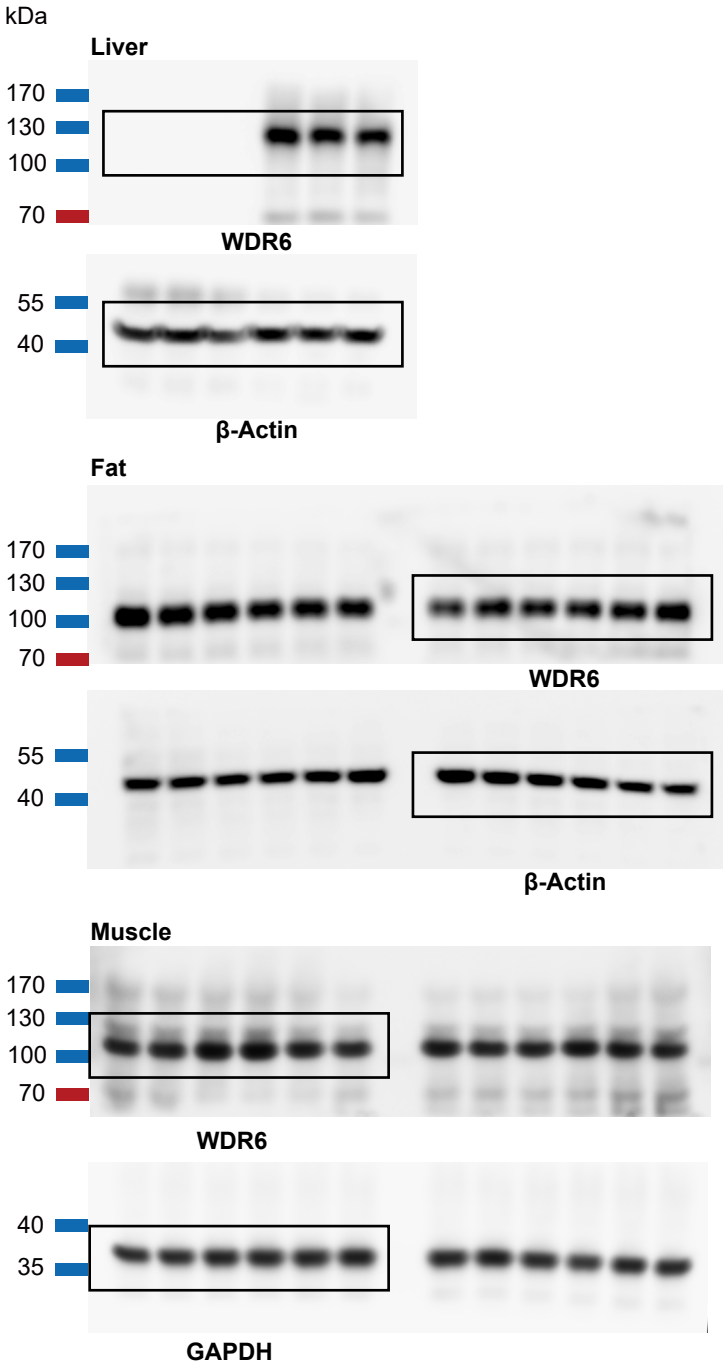

Supplement: Supplementary file 26 — Unprocessed western blots. [file 42255_2023_896_MOESM26_ESM.pdf]

Extended Data Fig. 6

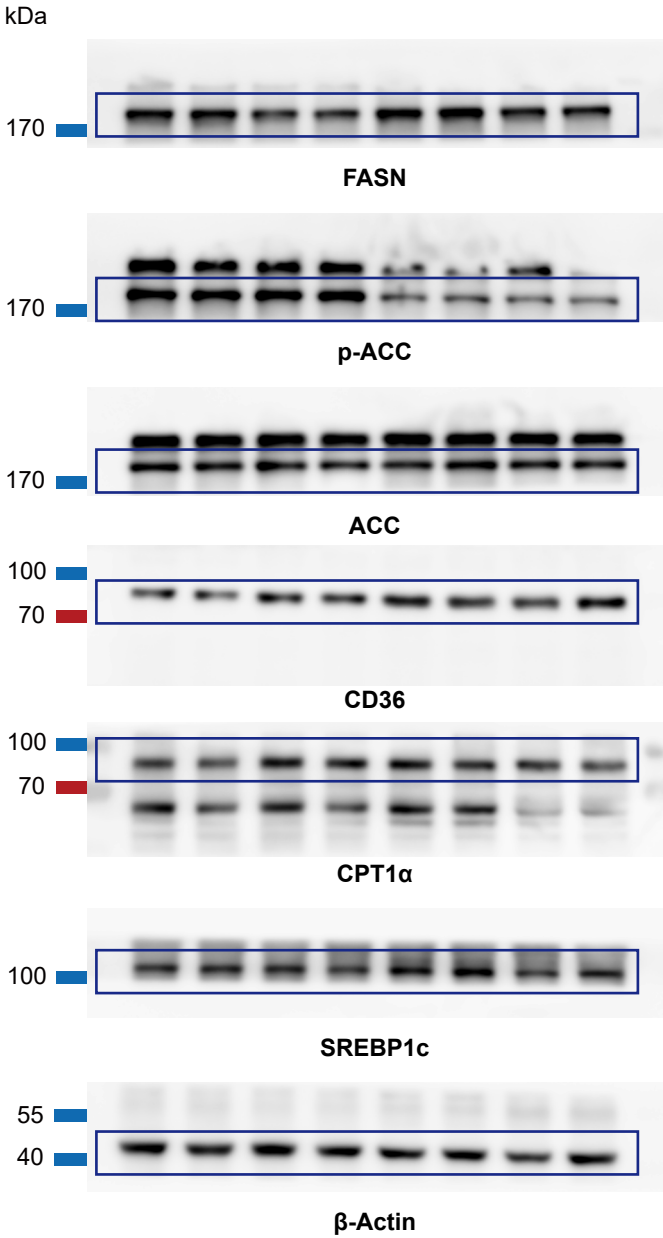

Supplement: Supplementary file 28 — Unprocessed western blots. [file 42255_2023_896_MOESM28_ESM.pdf]

**Extended Data Fig. 7i**

kDa

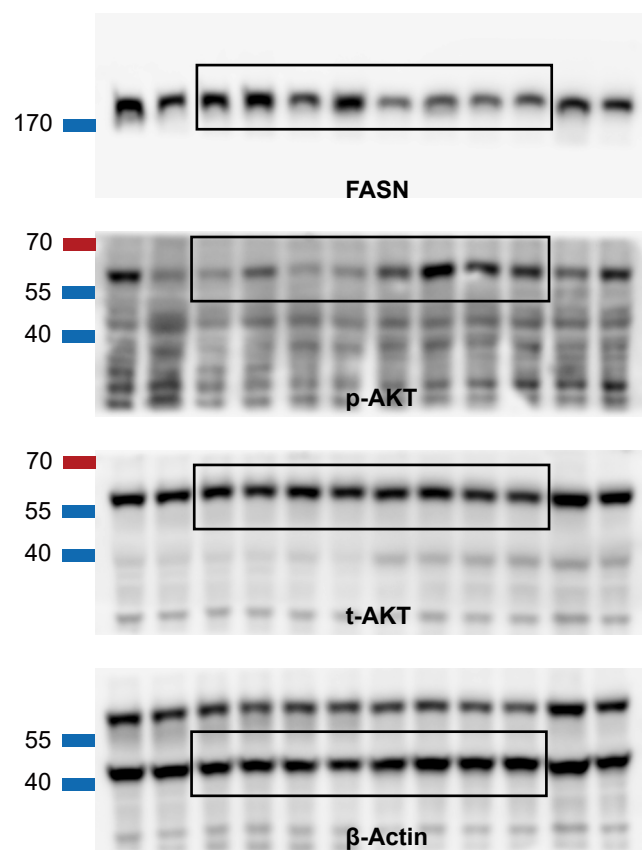

**Extended Data Fig. 7r**

kDa

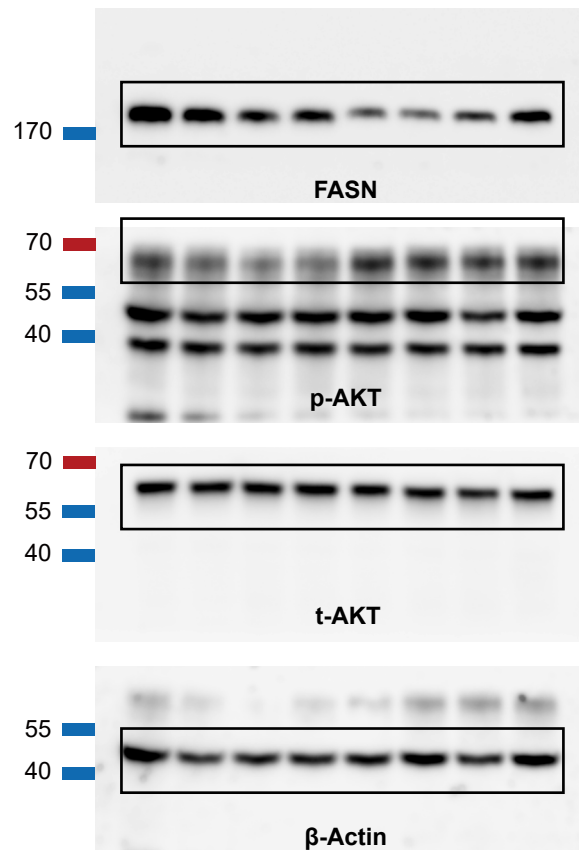

Supplement: Supplementary file 30 — Unprocessed western blots. [file 42255_2023_896_MOESM30_ESM.pdf]

Extended Data Fig. 10b

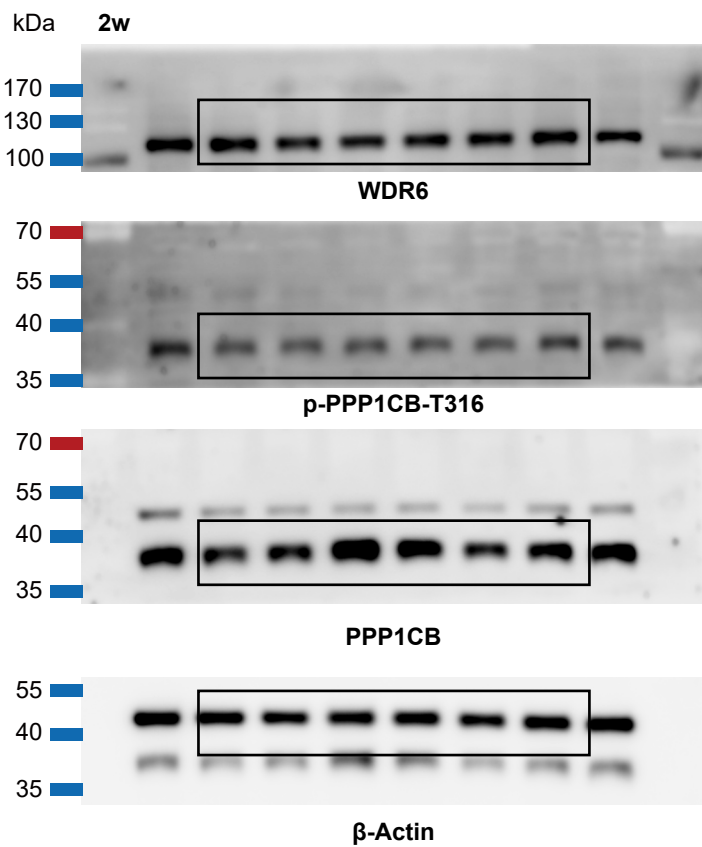

Extended Data Fig. 10b

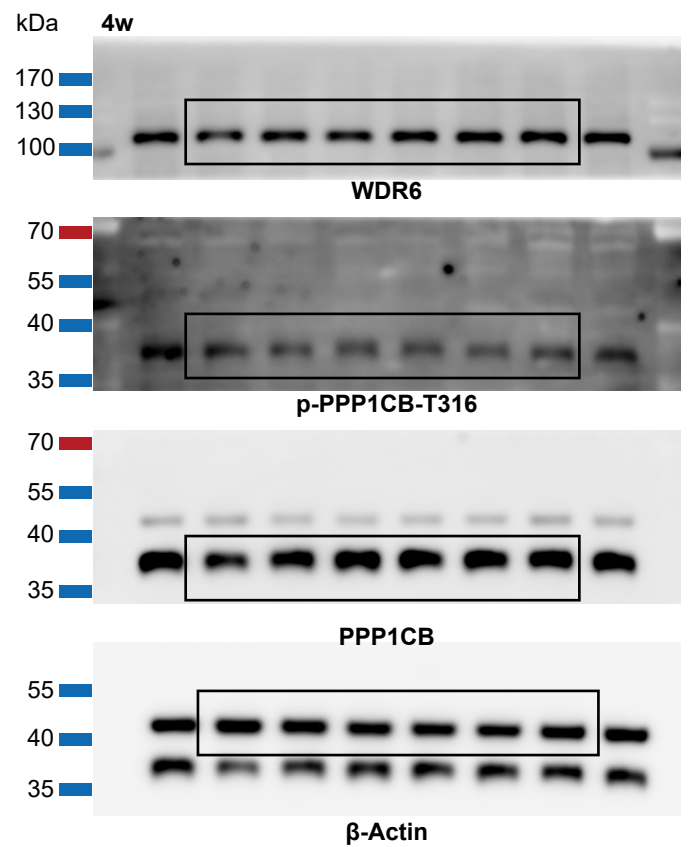

Extended Data Fig. 10b

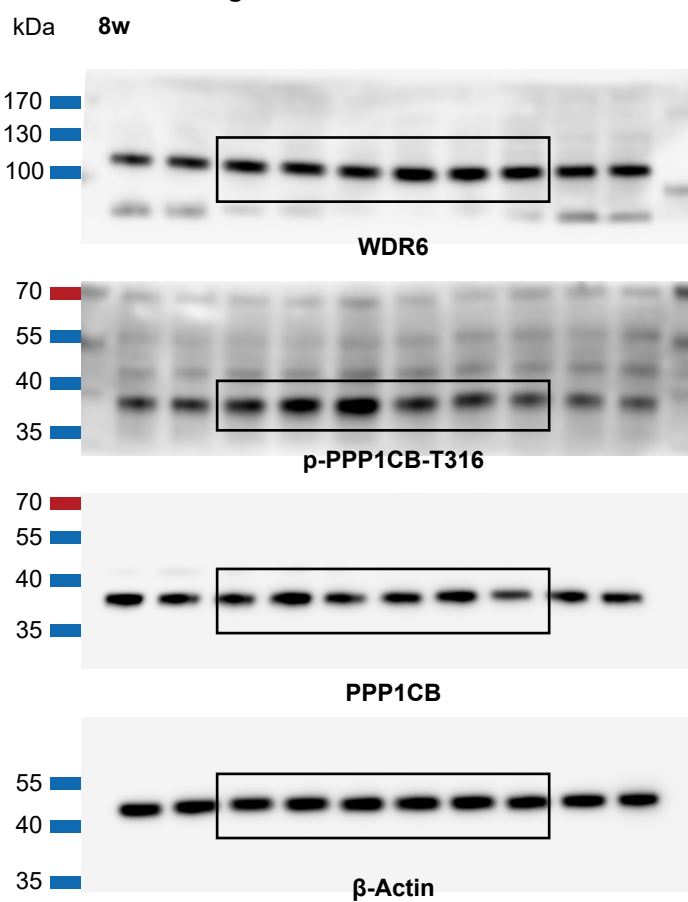

Extended Data Fig. 10b

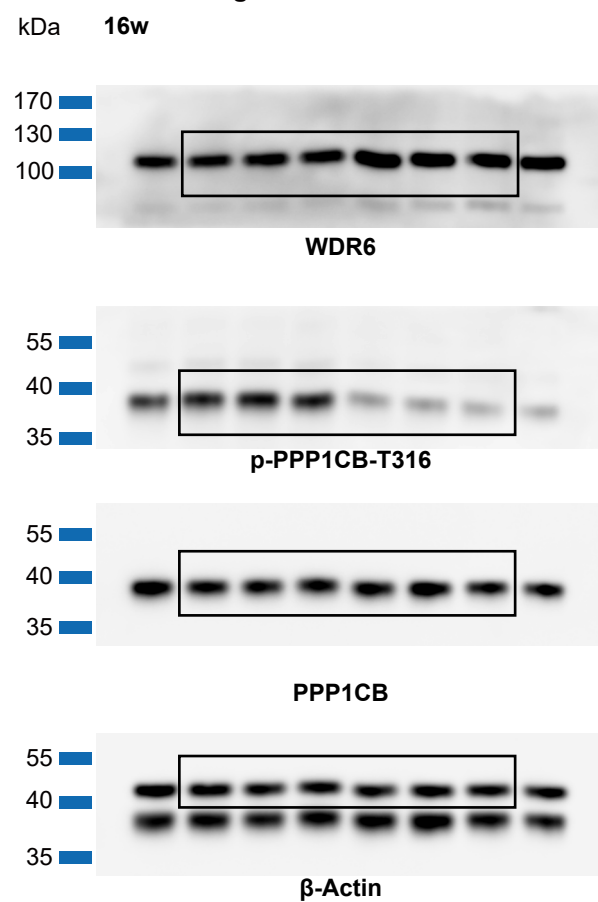

Supplement: Supplementary file 33 — Unprocessed western blots. [file 42255_2023_896_MOESM33_ESM.pdf]
